# Ancestral origins and post-admixture adaptive evolution of highland Tajiks

**DOI:** 10.1093/nsr/nwae284

**Published:** 2024-08-20

**Authors:** Jia Wen, Jiaojiao Liu, Qidi Feng, Yan Lu, Kai Yuan, Xiaoxi Zhang, Chao Zhang, Yang Gao, Xiaoji Wang, Dolikun Mamatyusupu, Shuhua Xu

**Affiliations:** State Key Laboratory of Genetic Engineering, Human Phenome Institute, Zhangjiang Fudan International Innovation Center, Center for Evolutionary Biology, School of Life Sciences, Department of Liver Surgery and Transplantation, Liver Cancer Institute, Zhongshan Hospital, Fudan University, Shanghai 200032, China; State Key Laboratory of Genetic Engineering, Human Phenome Institute, Zhangjiang Fudan International Innovation Center, Center for Evolutionary Biology, School of Life Sciences, Department of Liver Surgery and Transplantation, Liver Cancer Institute, Zhongshan Hospital, Fudan University, Shanghai 200032, China; School of Life Science and Technology, ShanghaiTech University, Shanghai 201210, China; Key Laboratory of Computational Biology, Shanghai Institute of Nutrition and Health, University of Chinese Academy of Sciences, Chinese Academy of Sciences, Shanghai 200031, China; State Key Laboratory of Genetic Engineering, Human Phenome Institute, Zhangjiang Fudan International Innovation Center, Center for Evolutionary Biology, School of Life Sciences, Department of Liver Surgery and Transplantation, Liver Cancer Institute, Zhongshan Hospital, Fudan University, Shanghai 200032, China; Ministry of Education Key Laboratory of Contemporary Anthropology, Fudan University, Shanghai 200438, China; Key Laboratory of Computational Biology, Shanghai Institute of Nutrition and Health, University of Chinese Academy of Sciences, Chinese Academy of Sciences, Shanghai 200031, China; School of Life Science and Technology, ShanghaiTech University, Shanghai 201210, China; Key Laboratory of Computational Biology, Shanghai Institute of Nutrition and Health, University of Chinese Academy of Sciences, Chinese Academy of Sciences, Shanghai 200031, China; Key Laboratory of Computational Biology, Shanghai Institute of Nutrition and Health, University of Chinese Academy of Sciences, Chinese Academy of Sciences, Shanghai 200031, China; State Key Laboratory of Genetic Engineering, Human Phenome Institute, Zhangjiang Fudan International Innovation Center, Center for Evolutionary Biology, School of Life Sciences, Department of Liver Surgery and Transplantation, Liver Cancer Institute, Zhongshan Hospital, Fudan University, Shanghai 200032, China; Key Laboratory of Computational Biology, Shanghai Institute of Nutrition and Health, University of Chinese Academy of Sciences, Chinese Academy of Sciences, Shanghai 200031, China; College of the Life Sciences and Technology, Xinjiang University, Urumqi 830046, China; State Key Laboratory of Genetic Engineering, Human Phenome Institute, Zhangjiang Fudan International Innovation Center, Center for Evolutionary Biology, School of Life Sciences, Department of Liver Surgery and Transplantation, Liver Cancer Institute, Zhongshan Hospital, Fudan University, Shanghai 200032, China; School of Life Science and Technology, ShanghaiTech University, Shanghai 201210, China; Key Laboratory of Computational Biology, Shanghai Institute of Nutrition and Health, University of Chinese Academy of Sciences, Chinese Academy of Sciences, Shanghai 200031, China

**Keywords:** Tajiks, population structure, genetic admixture, highland, local adaptation, epidermis protection, cardiovascular system

## Abstract

It remains debatable how many genes and how various the mechanisms are behind human adaptation to extreme environments, such as high altitudes. Despite extensive studies on Tibetans, Andeans and Ethiopians, new insights are expected to be provided with careful analysis of underrepresented highlanders living in a different geographical region, such as the Tajiks, who reside on the Pamir Plateau at an average altitude exceeding 4000 meters. Moreover, genetic admixture, as we observed in the current whole-genome deep-sequencing study of Xinjiang Tajiks (XJT), offers a unique opportunity to explore how admixture may facilitate adaptation to high-altitude environments. Compared with other extensively studied highlanders, XJT showed pronounced admixture patterns: most of their ancestry are derived from West Eurasians (34.5%–48.3%) and South Asians (21.4%–40.0%), and some minor ancestry from East Asians and Siberians (3.62%–17.5%). The greater genetic diversity in XJT than in their ancestral source populations provides a genetic basis for their adaptation to high-altitude environments. The admixture gain of functional adaptive components from ancestral populations could facilitate adaptation to high-altitude environments. Specifically, admixture-facilitated adaptation was strongly associated with skin-related candidate genes that respond to UV radiation (e.g. *HERC2* and *BNC2*) and cardiovascular-system-related genes (e.g. *MPI* and *BEST1*). Notably, no adaptive variants of genes showing outstanding natural selection signatures in the Tibetan or Andean highlanders were identified in XJT, including *EPAS1* and *EGLN1*, indicating that a different set of genes contributed to XJT's survival on the Pamir Plateau, although some genes underlying natural selection in XJT have been previously reported in other highlanders. Our results highlight the unique genetic adaptations in XJT and propose that admixture may play a vital role in facilitating high-altitude adaptation. By introducing and elevating diversity, admixture likely induces novel genetic factors that contribute to the survival of populations in extreme environments like the highlands.

## INTRODUCTION

Our knowledge of human adaptation to extreme environments, such as high altitudes, is still very much in its infancy and controversial. Recent studies have uncovered different strategies and genes among Tibetan, Andean and Ethiopian populations for adaptation to high-altitude environments, although little is known about the functional variants and genetic mechanisms involved [1–8]. Studying those under-investigated highland groups might provide new insights into the genetic basis of high-altitude adaptation. Tajiks, who speak a language from the Iranian branch of the Indo-European language family, are widely distributed in Tajikistan, Afghanistan, Uzbekistan and Xinjiang, China. Compared with Western Tajiks, Xinjiang Tajiks (XJT) living in the Tashkurgan County on the Pamir Plateau are identified as highland Tajiks, with an average altitude of more than 4000 meters due to the convergence of the Tian Shan, the Hindu Kush and the Kunlun Mountains near the county. Given this high elevation, XJT experience long-term high-altitude hypoxia, low temperatures and overexposure to ultraviolet (UV). Therefore, these individuals have greater vital capacity, blood pressure and heart rate than the national average at the same age. These physiological differences suggest that XJT have undergone physical adaptations to cope with the challenges of high-altitude living. Some studies have demonstrated that XJT show adaptation in hematological parameters including arterial oxygen saturation, red blood cell counts and hemoglobin concentration [9].

In genomic studies, a previous study indicated that *PPARG* could be a candidate for high-altitude adaptation in XJT [10]. In another study, no evidence was found to support selection in the mitochondrial DNA (mtDNA) of Pamir populations compared to that of lowlanders [11]. In contrast, Chen *et al.* revealed that high-altitude Tajiks display patterns of highland adaptation different from those of Tibetan highlanders in genes related to the oxidative phosphorylation pathway encoded by mtDNA [12]. Overall, the genetic basis of the high-altitude adaptations of XJT remains unclear.

Moreover, previous studies of Tajiks largely focused on the population's specific admixture pattern as one of the Central Asian populations [11,13–17]. Tajiks have been reported to be an admixed population with ancestries derived mainly from Eastern and Western Eurasian people, with a greater proportion of ancestry derived from West Eurasia than from other Central Asian populations, such as Uyghurs and Kazakhs [14]. One recent study demonstrated the genetic contribution of ancient DNA to present-day Tajiks and revealed that Tajiks can be traced back to the admixture of the Bronze Age Bactria-Margiana Archaeological Complex (BMAC) and Andronovo-related populations [18]. Additionally, there are differences between highland and lowland Tajiks [18,19]. Located in Xinjiang, a melting pot of human contact, populations such as Uyghurs and Kazakhs have been reported to have experienced multiple waves of admixture [20,21], while detailed studies on XJT remain limited. In addition, XJT represent a unique case for studying high-altitude adaptation due to their pronounced admixture patterns. As an admixed population, XJT provide a valuable opportunity to explore how genetic diversity introduced through admixture may facilitate adaptation to extreme environments. However, the genetic basis of XJT's high-altitude adaptation remains poorly studied. In this study, we sequenced 26 XJT with high coverage (>30 ×) and genotyped 48 XJT using an Affymetrix Genome-Wide Human SNP 6.0 array. We then comprehensively characterized the ancestral makeup of XJT and the footprints of natural selection in their genomes.

## RESULTS

### Genetic affinity and population structure of Xinjiang Tajiks

**Figure 1.**
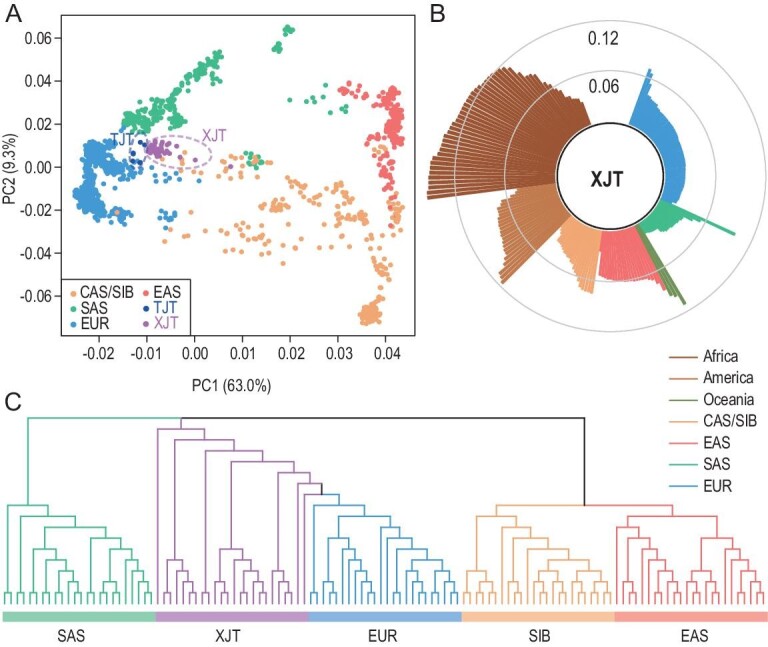
Genetic affinity of Tajiks in the context of global populations. (A) PCA of 46 XJT with reference populations from Central Asia (CAS)/Siberia (SIB), East Asia (EAS), South Asia (SAS) and West Eurasia (EUR). XJT and TJT are highlighted. The number in the bracket represents the variance explained by each PC accounting for the top 10 PCs. XJT: Xinjiang Tajiks, TJT: Tajikistan Tajiks. (B) *F*_ST_ between XJT and other populations worldwide. The length is proportional to the *F*_ST_ value, as indicated by the gray circles. (C) A neighbor-joining tree of XJT, EUR, SAS, EAS and SIB was constructed based on the identical-by-state matrix.

To understand the general patterns of relatedness between XJT and worldwide populations, we analyzed genome-wide data from XJT and 203 worldwide populations from the Human Origins data set [22]. The reference populations were classified into seven groups representing major geographical regions: Africa, America, Oceania, Central Asia (CAS)/Siberia (SIB), East Asia (EAS), South Asia (SAS) and West Eurasia (EUR). Principal component analysis (PCA) revealed that XJT lies along the axis between groups from EUR and EAS (Fig. S1), indicating genetic admixture of the Western and Eastern Eurasian people. After populations outside of Eurasia were removed from the PCA, the XJT samples were surrounded by populations from SIB, EAS, SAS and EUR (Fig. 1A). Among these neighboring populations, XJT had the closest relationship with EUR/SAS, which was confirmed by the *F*_ST_ results (Fig. 1B, Fig. S2) and individual tree analysis (Fig. 1C). The site frequency spectrum (SFS) of XJT is also correlated with that of EUR and SAS (Fig. S3).

We noticed that XJT tended to have a closer relationship with East Asian populations than Tajikistan Tajiks (TJT) (Fig. 1A), suggesting that the gene pool of XJT has been influenced by East Asian ancestry. Interestingly, we found that TJT was not the group with the closest relationship with XJT (*F*_ST_ = 0.0180); instead, Pathan (*F*_ST_ = 0.0135) and Turkish (*F*_ST_ = 0.0136) populations showed much less differentiation from XJT (Fig. S2). In contrast, XJT was most closely related to TJT, followed by Turkish (*F*_ST_ = 0.0211) and Pathan (*F*_ST_ = 0.0221) populations (Fig. S4).

### Functional loci in XJT

Taking advantage of the XJT-sequenced data, we identified ∼2.89 million single-nucleotide polymorphisms (SNPs) at the individual level (Fig. S5) and ∼7.28 million SNPs at the population level, ∼0.19% of which were novel single-nucleotide variants (SNVs) (13 703) showing high pathogenicity, conservation and low allele frequency (Fig. S6), indicating that novel SNVs may play important biological functions.

In addition, we identified 658 loss-of-function (LOF) mutations, all of which are classified as known SNVs and primarily feature low allele frequencies (Fig. S7). These variants are primarily characterized as splicing acceptors/donors, or stop-gain variants according to variant annotation. We identified eight LOF variants (Fig. S8) with high allele frequencies in XJT (>0.1) and low allele frequencies in worldwide populations (<0.1); these variants are located in eight different genes, some of which are involved in blood pressure regulation (*KLK14*), keratinization (*TLN2, KLK14*) and antioxidative reactions (*PXDNL*), potentially favoring high-altitude adaptation for XJT in response to hypoxia and high UV radiation. In addition to these eight LOF variants, another LOF variant (rs78180793, chr10 : 48 359 449: T > C), a splice donor variant, exhibits a high allele-C frequency in XJT (0.46, Fig. S9) but is absent in worldwide populations. This LOF mutation is also in an allele frequency block, implying that the haplotype shaped by these alleles may experience positive selection. The associated gene *ZNF488* encodes a zinc finger protein that promotes the differentiation of adult neural stem progenitor cells (NSPCs) into mature oligodendrocytes and contributes to remyelination following nerve injury. We speculate that this selected haplotype may play an important role in protection against hypoxia.

Furthermore, eight clinically protective variants with a high allele frequency in XJT (>0.1), high conservation (GERP++ score > 2) and high pathogenicity (CADD score > 15) were filtered (Fig. S10). Notably, only these eight variants exhibit these properties, most of which are moderate for allele frequency in XJT compared with in Western and Eastern Eurasian populations. These variants are associated with pigmentation, alcohol metabolism, plasma vitamin B12, and HIV1 resistance protection against several complex diseases (e.g. diastolic hypertension, coronary artery disease, multiple myeloma and metabolic syndrome, Table S1).

In conclusion, we dissected the general pattern of functional loci of XJT (novel SNVs, LOF variants and clinic-protective variants), some XJT-enriched functional loci and their associated traits/diseases. We speculate that some of them are beneficial to the high-altitude adaptation of XJT.

### Genetic composition and admixture of XJT

To unveil the ancestral make-up of XJT and investigate genetic influence from surrounding populations, we performed ADMIXTURE analysis for XJT together with global populations, assuming a different number of ancestral source populations (K from 2 to 20) (Fig. S11).

**Figure 2.**
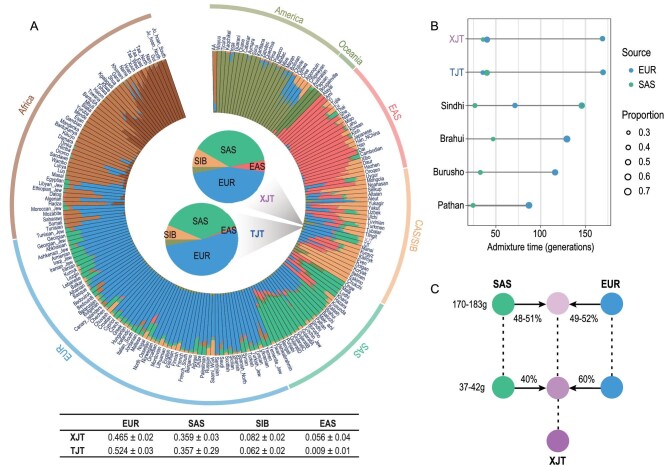
Ancestry make-up and admixture model of XJT. (A) ADMIXTURE results of XJT and TJT with global populations at K = 8. The results of the population-level admixture of XJT and TJT are summarized and displayed in the pie charts in the center of the circle plot. Admixture proportions are denoted with different colors, with the corresponding proportions detailed in the table below. (B) Estimation of admixture events for XJT, TJT and other admixed populations. Sindhi, Brahui, Burusho and Pathan are populations in Pakistan, and their gene pools are similar to those of XJT and TJT and are mostly derived from ancestors in SAS and EUR. (C) Admixture model of XJT.

From the ADMIXTURE results assuming greater Ks, we observed that most Central Asian populations share most of their ancestry with populations from EAS, SIB, EUR and SAS (Fig. S11). Notably, XJT consistently had more ancestry from EUR (46.5%) and SAS (35.9%) and less ancestry from EAS (5.6%) and SIB (8.2%) (Fig. 2A). These findings are consistent with those of previous studies of Central Asian populations, in which Tajiks were found to be most closely related to those in Western Eurasian populations [14,15]. Tajiks are the only population in the region that speaks an Indo-European language, while other Central Asian populations such as the Uyghurs, Kazakhs and Kyrgyz speak Turkic languages. This suggests that the influence on Tajiks of Turkic expansion over the last two millennia might have been less significant than that on other Central Asian populations.

XJT and TJT both speak Iranian; however, they speak distinct languages within this subgroup. XJT predominantly use languages from the Eastern Iranian branch, while TJT generally speak languages from the Western Iranian branch. Compared with XJT, the TJT populations share most of their ancestry with populations from EUR (52.4%) and SAS (35.7%), a smaller contribution from SIB (6.2%) and almost no contribution from EAS (0.9%) (Fig. 2A). This difference in ancestry between XJT and TJT likely resulted from gene flows from neighboring populations after population divergence. This result is consistent with the findings from a previous study based on ancient DNA, which suggested that highland Tajik populations received additional gene flow from an ancient North-Eurasian-related population [18].

As most of the ancestral makeup of XJT was derived from EUR and SAS, we further investigated the admixture parameters using MultiWaver 2.0 [23], assuming that Mala/Vishvabrahmin and Sardinian/Basque are presentative populations of SAS and EUR ancestry because these populations possess a high level of pure SAS or EUR ancestral components (>90%). The results indicated a gradual admixture (GA) model, showing that the first EUR–SAS admixture, half with EUR ancestry and half with SAS ancestry, occurred 170–183 generations ago (Ga, 25 years/generation), and the second occurred at 37–42 Ga with a contribution ratio of 3 : 2 (Fig. 2B and C). Furthermore, the admixture model of TJT was the same as that of XJT (Fig. 2B), consistent with the results of PCA and *F*_ST_, suggesting that XJT and TJT split very recently (<37 Ga). However, as EUR–SAS-admixed people, populations from Pakistan (Sindhi, Brahui, Burusho and Pathan) showed different admixture models from those of XJT, and admixture events were estimated in chronological order (Fig. 2B), indicating that they experienced different admixture histories, possibly due to different geographical distributions. The second admixture event was replicated by ALDER [24] according to admixture-induced linkage disequilibrium (LD) decay (Table S2). Since XJT received a genetic contribution from Eastern Eurasia (∼14%), we also estimated admixture parameters under the east‒west admixture model (Table S3). XJT received a gene flow from East Asians at 20–30 Ga, which could explain why this population shares more pairwise identity-by-descent (IBD) segments with East Asians than other groups (Fig. S13).

### The impact of admixture on genome diversity

**Figure 3.**
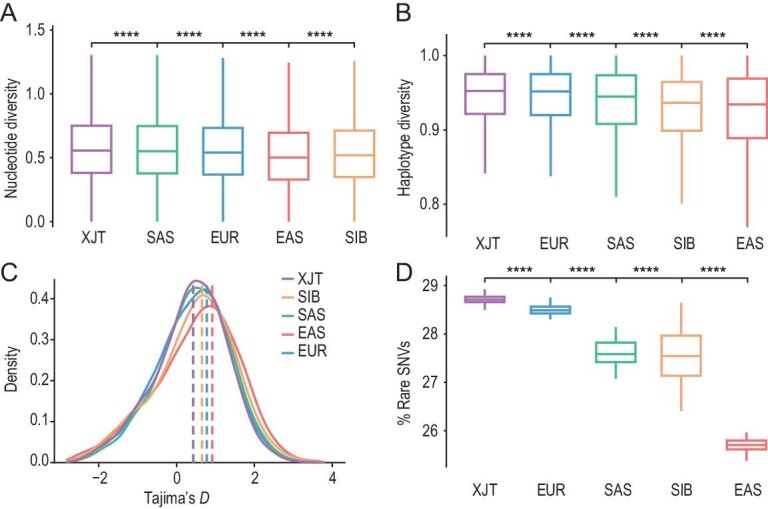
Admixture-driven genetic diversity of XJT. (A) Nucleotide diversity (θ_π_/kb) of XJT, EUR, SAS, EAS and SIB, estimated within 50 kb sliding windows shifted by 25 kb across the genome. Significance determined by the Wilcoxon rank-sum test: ns: *P* > 0.05; ∗: *P* ≤ 0.05; ∗∗: *P* ≤ 0.01; ∗∗∗: *P* ≤ 0.001; ∗∗∗∗: *P* ≤ 0.0001. (B) Haplotype diversity of XJT and the reference populations. (C) Distribution of Tajima's *D* statistics. (D) Rare SNV proportions in XJT and the reference populations. It was calculated as the proportion of rare SNVs (AF < 0.05).

Generally, divergent ancestral components introduced by admixture can result in a high level of genetic diversity in admixed populations. We found that the nucleotide diversity (θ_π_), haplotype diversity (H) and number of segregating sites (θ_k_) were greater in XJT than in the reference populations (*P* value < 0.01) (Fig. 3A and B, Fig. S14). Tajima's *D* statistic showed a negatively skewed distribution (Fig. 3C), and the θ_π_ in XJT was greater than the θ_k_, suggesting that XJT has specific SNVs from diverse ancestral populations. XJT also harbored the greatest proportion of rare SNVs (allele frequency (AF) < 0.05), followed by EUR. This observation lends support to the hypothesis that admixture may contribute to the enrichment of rare variants within populations (Fig. 3D). Previous studies have observed the same phenomenon in typically admixed populations, such as Uyghur and African American populations [25]. We also estimated the heterozygosity of genome-wide SNPs. XJT showed higher heterozygosity than the other populations (*P* value < 0.01) and a similar level to that of SAS (Fig. S15), indicating that genetic admixture enhanced the genetic diversity of XJT and that South Asian ancestries contributed more diversity.

Nevertheless, XJT is relatively isolated on the plateau, prompting us to investigate whether this population experienced inbreeding. Previous studies have reported that Tajiks began practicing endogamy after the admixture of BMAC and Andronovo populations [18]. In this study, we found that the genome of XJT contains more intermediate (2–10 Mb) and long (>10 Mb) runs of homozygosity (ROHs) than the eastern and western Eurasian populations (Fig. S16A). The *N*e of XJT is significantly smaller than that of African, American, East Asian and Western European populations (Fig. S16B), and the inbreeding coefficients of XJT also exceed those of other populations (Fig. S16C), indicating that XJT is a small population and that inbreeding phenomena exist within it. This result is consistent with previous reports [18], and the patterns are in accordance with the endogamous marriage patterns in Tajiks [26]. Vast long ROHs in populations from SAS suggest that cultural factors (e.g. caste system and religion) could influence genetic diversity [27,28].

### Post-admixture adaptive evolution in XJT

**Figure 4.**
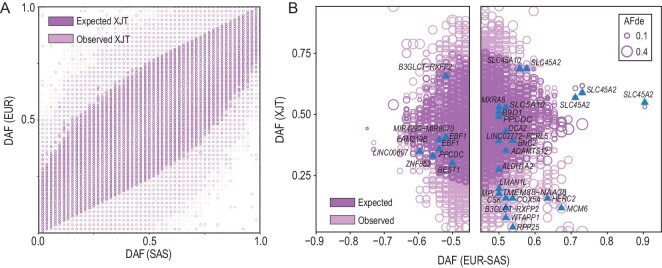
The post-admixture genome-wide frequency profile of XJT. (A) Site frequency spectrum of the frequency profiles among EUR, SAS, XJT, and the expected XJT genome. (B) Derived allele frequency (DAF) of SNVs showing extreme frequency differences between EUR and SAS, which are also recorded in the GWAS catalog (v1.0.2). Genes related to the cardiovascular system or skin protection are labeled in the figure.

Several studies have indicated that admixed populations, such as African Americans [29] and Latin Americans [30], have undergone rapid evolution after admixture. The high genetic diversity of XJT may also have influenced adaptive evolution to some extent. The AF of XJT is expected to be the average of those in the ancestral source populations weighted by the global admixture proportions. Analysis of the SFS of the XJT, EUR and SAS populations showed that XJT had an overall frequency profile close to the expected distribution (Fig. 4A); this phenomenon is consistent with the ‘rules of admixture’ proposed by Pan *et al.* [25]. Nonetheless, the frequencies of several local genetic components deviated significantly from the expected values. These deviations are likely attributable to the adaptive pressures exerted by high-altitude environments. Furthermore, we calculated the AF deviation from expectation (AFd_e_) and performed an enrichment analysis. We estimated the proportion of SNVs with a significant AFd_e_ in each gene region and ranked these genes accordingly. Modified gene set enrichment analysis (mGSEA) [25,31] was used for the pathway analysis to determine genes enriched in a significant AFd_e_, using the KEGG pathway data set as the reference [32]. A total of 182 pathways (Table S4) were enriched in genes with high AFd_e_. For example, genes in pathways including the ‘nervous system’, ‘cardiovascular system’, ‘immune system’, ‘thermogenesis’ and ‘melanogenesis’ were identified with high AFd_e_ across the genome. This phenomenon may be related to the high-altitude adaptation of XJT.

Analysis of the ancestry-divergent SNVs recorded in the Genome-Wide Association Studies (GWAS) Catalog also revealed that the majority of the functionally important genetic components followed the ‘rules of admixture’, whereas some demonstrated unexpected frequencies in XJT, including *B3GLCT, RPP25, CSK, BNC2, HERC2* and *SLC45A2*. Notably, several of these SNVs have been previously reported to be associated with skin diseases and the cardiovascular system (Fig. 4B).

Genetic variations in the *HERC2* gene are associated with skin pigmentation variability. The variant rs12916300, located within *HERC2*, has been reported to be associated with the development of keratinocyte cancer and squamous cell carcinoma [33,34], with allele-T identified as the risk allele. In XJT, the frequency of allele-T (0.192) was significantly lower than that of allele-C (0.808), suggesting that this allele plays a protective role against the development of skin cancer in XJT. Interestingly, the observed frequency of allele-T was lower than the expected frequency (0.402) post-admixture, indicating an AF deviation that reduces the probability of skin cancer occurrence in high-altitude environments, where there is a high intensity of UV radiation. Several loci on *HERC2* have also been reported to be associated with traits such as skin sensitivity to sun and sunburn [35–38].

Mutations in *BNC2* are related to facial pigmented spots, and this gene is likely a transcription factor specific for skin keratinocytes. Diseases associated with *BNC2* include keratinocyte cancer [33], non-melanoma skin cancer [39] and skin cancer [40], all of which are related to exposure to intense sunlight. The variant rs2026805 in *BNC2* has been reported to be associated with keratinocyte carcinoma [33]. The risk allele is allele-A, and the observed frequency (0.403) of allele-A was lower than the expected frequency (0.584) post-admixture, indicating an AF deviation in high-altitude environments. This deviation may decrease the probability of developing skin cancer under high UV radiation conditions.

Moreover, post-admixture adaptive evolution was also observed in pathways related to the cardiovascular system. For instance, rs1127796, a 3 prime UTR variant of the *MPI* gene, has been reported to be associated with red cell distribution width (RDW) [41]. The observed frequency of rs1127796-T in XJT (0.818) was greater than its expected post-admixture frequency (0.561). This AF deviation significantly reduces the expression of *MPI* in different tissues (Fig. S17), consequently affecting RDW in XJT. This reduced expression may contribute to the adaptation of XJT to hypoxic conditions in high-altitude environments. Adjacent to *MPI*, the *FAM219B* gene also harbors several functional loci with AF deviations. For instance, rs6495128, previously reported to be associated with hemoglobin levels [36,41,42], showed an increase in the AF of allele-T post-admixture. Additionally, rs10628234 and rs7497026 in this gene are associated with blood pressure [43,44]. We also observed that rs1109748, located in the *BEST1* gene, is associated with plasma omega-3 polyunsaturated fatty acid levels [45], which are related to cardiovascular disease risk and cognitive function in the brain. This gene is expressed at high levels in the brain, nerves and whole blood (Fig. S18). The observed frequency of allele-A (0.434) on rs1109748 exceeded its expected frequency (0.301), with an increase in allele-A frequency potentially enhancing *BEST1* gene expression (Fig. S19). Interestingly, two variants (rs2736600 and rs2736598) located in the genomic region of *BEST1* are in complete linkage disequilibrium with two dominant CT/TC haplotypes (r2 = 1, D′ = 1), which also exhibit AF deviation (AFd_e_ = 0.181). These variants were previously reported to be associated with lung function [36,46]. We speculate that this variation may offer protective advantages for XJT in hypoxic environments. Overall, the deviation in AFs post-admixture benefits the survival of XJT in high-altitude environments.

To broaden our understanding, we considered both within-population and between-multiple-population comparisons and performed a genome-wide scan to detect strong signals of selective sweeps in XJT using composite of multiple signals (CMS) statistics [47], assuming that a candidate genomic region should have a significantly higher prioritization rank in several scanning methods simultaneously. For statistical analysis of within-population (integrated haplotype score, iHS) genomes, only 24 sequenced genomes of XJT were included; otherwise, we randomly selected 24 published genomes of reference populations [48] as a reference (AFd_e_; *F*_ST_; cross-population extended haplotype homozygosity (XP-EHH). In total, we identified 923 significant gene regions by the CMS statistic, with a *P* value < 0.05 according to the empirical distribution of gene-level CMS values from genes with similar SNP numbers (Table S5, Fig. S20).

**Figure 5.**
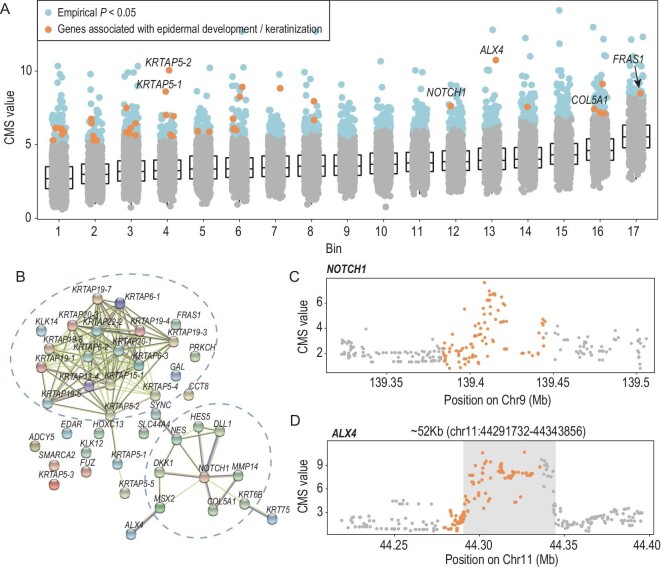
The candidate genes associated with epidermal development and keratinization. (A) Candidate genes involved in skin development (43 genes). X-axis: bins clustered by gene length; Y-axis: gene-level CMS value (MaxCMS), the maximum CMS value of SNPs in each gene. (B) Protein‒protein interaction network of significant gene regions related to skin development. (C) CMS values of SNPs located in the *NOTCH1* gene region. *NOTCH1* was the central gene of the PPI subnetwork. (D) CMS values of SNPs located in the *ALX4* gene region.

A gene set enrichment analysis of the 923 gene regions was performed to associate the gene regions with biological functions. We identified multiple biological processes highly related to highland adaptation (Fig. S21), including skin development and sprouting angiogenesis, embryonic organ development and skeletal system morphogenesis. In particular, 43 gene regions associated with epidermis development and keratinization were enriched in the intermediate filament cytoskeleton and intermediate filament of Gene Ontology (GO) Cellular Component (CC) terms (Fig. 5A, Fig. S22, Table S6). We speculate that epidermal protection against UV radiation is beneficial for the survival of XJT in the highlands, through epidermal thickness [49,50]. The protein‒protein interaction network showed two clusters: the KRTAPs gene family and a subnetwork centered on *NOTCH1* (Fig. 5B and C). In addition, a region with a length of 52 kb (chr11 : 44291732–44343856), located in *ALX4*, was expected to be under positive selection (Fig. 5D), with an obvious peak in the CMS values. The *ALX4* gene encodes a member of the homeobox protein family. Homeobox proteins direct the formation of body structures during early embryonic development. And this protein is involved in the formation of skin layers, according to MedlinePlus (https://medlineplus.gov/). We also detected strong selection signals for other skin-related genes (e.g. *COL5A1* and *FRAS1*) in all methods (Fig. S23). We hypothesize that these genes may play an important role in skin protection in XJT. Moreover, previous studies revealed that quantitative trait loci, such as rs11037965 [51] on *ALX4* and rs186222325 [42] on *FRAS1*, are related to the cardiovascular system.

Finally, to characterize the polygenic effects of genetic adaptation in XJT, mGSEA was performed on genes ranked in descending order of CMS, and 19 biological pathways were identified (Table S7). The pathways involved included the immune system, signal transduction, metabolism and others. This finding suggests that the immune and nervous systems play important roles in the adaptation of XJT to extreme environments.

### Archaic ancestry in the XJT

Previous studies have indicated that modern humans acquired beneficial genetic components for environmental adaptation, such as adaptation to high-altitude conditions, through gene flow with archaic humans ∼40 000 years ago [52,53]. We thus further detected archaic segments locally in the genome of XJT using ArchaicSeeker 2.0 [54]. Globally, the proportion of Denisovan/Neanderthal ancestry in XJT is intermediate in its ancestral populations (Fig. S24), consistent with its admixture pattern. In detail, the Denisovan-like sequence proportion in XJT (mean: 0.10%, range: 0.08%–0.12%) was significantly greater than that in Europeans (mean: 0.06%, range: 0.05%–0.09%) (*P* value = 9.266 × 10^–15^) and less than that in South Asians (mean: 0.12%, range: 0.08%–0.16%) (*P* value = 1.43 × 10^–11^) and East Asians (mean: 0.14%, range: 0.10%–0.18%) (*P* value = 1.585 × 10^–14^). We also found that the proportion of Neanderthal-like sequences in XJT (mean: 1.20%, range: 1.11%–1.25%) was significantly greater than that in Europeans (mean: 1.16%, range: 1.07%–1.30%) (*P* value = 6.085 × 10^–5^), whereas it was significantly lower than that in South Asians (mean: 1.23%, range: 1.13%–1.34%) (*P* value = 0.0005383) and East Asians (mean: 1.49%, range: 1.38%–1.61%) (*P* value = 5.152 × 10^–15^).

Next, we filtrated archaic introgressed segments by overlaps between ArchaicSeeker 2.0 and Sprime [55] and estimated the expected archaic-like haplotype frequency for XJT according to the archaic-like haplotype frequencies and admixture proportions of its major ancestors. Then, we obtained one introgressed segment derived from Denisovan that was significantly enriched in XJT (Table S8). This ∼33 kb segment in *LRRC2* (a tumor suppressor gene) contained seven significant variants, and these variants were in absolute LD, showing the same archaic AFs in XJT (∼0.32) and other worldwide populations (<0.15) (Fig. S25). *LRRC2* encodes a member of the leucine-rich repeat-containing family of proteins that functions in diverse biological pathways and may be a tumor suppressor. *LRRC2* was found to be localized to the mitochondria in human cells and a regulator of the cardiac hypertrophic response [56]. Several SNVs in this gene have also been reported to be associated with blood protein levels (rs939421, rs4682823) [57,58] or coronary artery disease (rs112043140) [59,60].

In addition, we identified two Neanderthal-like segments that are enriched in XJT (∼0.44). These segments contain two genes, *SLC35B3* and *ZNF385D*, which were significant in the whole-genome scan based on gene-level CMS values and include notable variants (Table S9). *SLC35B3* (CMS value: 6.26) is a member of the solute carrier family. The encoded protein is involved in the transport of 3-prime phosphoadenosine 5-prime phosphosulfate (PAPS) from the nucleus or the cytosol to the Golgi lumen. Previous studies have revealed that SNVs in this gene are associated with the cardiovascular system [61] or lung function [36,62]. Another gene, *ZNF385D* (CMS value: 8.88), enables sequence-specific double-stranded DNA binding activity and is related to nucleic acid binding. Interestingly, we also found SNVs in this gene that have been reported to be related to lung function [36,62] or the cardiovascular system [63–65].

We also examined the joint distribution of the U and Q95 statistics [66], identifying the *NXNL2* region as the strongest candidate region. This region is in the 99.9% quantiles of the genome-wide distributions of both statistics, with Denisova being the closest source. *NXNL2* has been associated with various traits, including the respiratory system phenotype [67], cardiovascular system phenotype [68], integument or pigmentation [68], immune system phenotype [68] and nervous system phenotype [67]. We speculate that these archaic introgression segments may be beneficial for the adaptation of XJT to high-altitude environments.

### Shared selection signals between XJT and other highlanders or highland animals

Previous studies of high-altitude adaptation, mostly focused on Tibetans, Ethiopians and Andeans, have identified several candidate genes [69–77]. Thus, we investigated whether there are shared mechanisms for high-altitude adaptation between XJT and other highlanders. We summarized the candidate genes involved in high-altitude adaptation previously reported in Ethiopians, Andeans, Tibetans and Tibetan animals (Table S10). Among the candidate genes, we found that some genes previously reported also had selection signals in XJT (Tables S11–S12). These genes are assumed to be involved in different biological processes in response to hypoxia, high UV radiation and low temperature in highlands (Fig. S26). Compared to other genes, genes related to the cardiovascular system are the most numerous. A genetic region with a length of ∼100 kb in *ANK1* showed a significant high CMS value (Fig. S27). Three variants (rs4737009, rs4737010, rs1819953) located in the region were GWAS loci involved in mean corpuscular-hemoglobin concentration (MCHC) or mean red cell distribution width (MRDW). Therefore, we speculated that this gene underwent positive selection to protect XJT against hypoxia. Moreover, we found that the AF of SNPs in this region in XJT was strongly correlated with that in East Asia (Fig. S28), suggesting that genetic contributions from East Asia play an important role in the high-altitude adaptation of XJT, though the genetic contribution is very minor. Nevertheless, *EPAS1* (CMS value: 1.73) and *EGLN1* (CMS value: 2.49), which are famous adaptive genes in high-altitude adaptation studies, were not identified as significant genes. Analysis of the allele spectrum indicated that the genetic component within the two gene regions was in a state of expected admixture, with almost no AF deviation post-admixture (Fig. S29). This finding suggested that, unlike in Tibetans, *EPAS1* and *EGLN1* did not experience strong selection in the XJT response to environmental pressures. Key genetic variants did not accumulate further during the post-admixture evolutionary process. The AF of the tag SNPs in XJT was lower than that in Tibetans (Fig. S30), indicating that XJT may have a different adaptive genetic mechanism than other highlanders despite convergent evolution.

## DISCUSSION

We comprehensively characterized the genetic variation in 26 XJT samples. This valuable data set revealed the genetic origins and admixture history of XJT. In contrast to Turkic-speaking populations (e.g. Xinjiang Kazakhs and Xinjiang Uyghurs) [20,21], Tajiks in Xinjiang and Tajikistan are genetically close to West Eurasians, with minor ancestral components derived from East Eurasians. This finding suggests that Tajiks are significantly more distantly related to Eastern Eurasian populations than Turkic speakers are. These data suggest that language transmission involves not only cultural exchange but also genetic admixture and gene flow. Additionally, differences in language may act as barriers to gene flow. The genetic diversity within Central Asian populations correlates with linguistic classifications. Specifically, Indo-European-speaking Tajiks from Xinjiang and Tajikistan exhibit genetic homogeneity and are less influenced by East Asian Turkic-speaking populations.

When recombined in new environments, the genetic components derived from divergent ancestral populations may generate new biological functions and influence adaptive evolution post-admixture. Therefore, the XJT, as an admixed population residing in the highlands, plays a crucial role in research on admixture-driven adaptation in high-altitude environments. According to our data, the overall frequency profile of the XJT genome aligns with the ‘rules of admixture’ proposed by Pan *et al.* [25], with some of the genetic components undergoing adaptive evolution under the pressure of a high-altitude environment. The genetic components introduced by admixture have helped XJT survive better in specific environments.

We systematically searched for local adaptation signals and archaic introgression in XJT to determine their high-altitude adaptation mechanism. Population admixture can be considered a mechanism of adaptation to high-altitude environments, whereby different genetic components on the genomes on admixed populations are influenced by factors such as their ancestral origin and biological function and subsequently evolve in a more favorable direction. For example, several functional SNVs in the *HERC2* and *BNC2* genes were found to deviate from the expected AFs post-admixture, and genetic mutations in both genes are strongly associated with skin phenotypes [33–40]. According to previous studies, the direction of their allelic deviation can decrease the risk of skin cancers (e.g. melanoma and squamous cell carcinoma) in a high-UV environment. The integration of several different analyses (CMS method) also revealed several strongly selected skin-related genes, such as *NOTCH1* and *ALX4*, indicating that skin-phenotype-related pathways/variants are important for the adaptation of XJT to high-altitude environments. In addition to skin-related adaptations, we discovered several genes associated with the cardiovascular system (e.g. *MPI* and *BEST1*) showing post-admixture adaptation in XJT, consistent with our general knowledge that the cardiovascular system of highland populations is more adapted to their specific survival environment.

Among the genes showing strong selection signals in XJT, some have been previously reported in other highlanders or highland animals (Tables S9 and S10). Interestingly, XJT showed no obvious signals on star genes of high-altitude adaptation (e.g. *EPAS1* and *EGLN1*). This finding suggests that XJT only partially shares the genetic basis for high-altitude adaptation with other highlanders, although convergent adaptation might be prevalent.

In conclusion, the analysis of whole-genome deep sequencing and microarray data of the XJT in this study provides a comprehensive overview of the population's genetic structure, genetic diversity and admixture history at the genomic level, improving our understanding of the genetic origin and admixture history of the genome of ethnic minorities in Northwest China. Further breakthroughs may be possible in the future if a combination of ancient DNA from different periods in neighboring regions can be added for analysis. Additionally, population admixture has been proposed as a mechanism of adaptation to a new environment. Among the several highland populations, XJT represents a valuable case of an admixed population adapting to marginal and stressful environments through admixture. We expect that our findings will advance the understanding of the adaptive genetic mechanisms of the Pamir Plateau population and contribute to a deeper understanding of the adaptations of other populations living in extreme environments.

## MATERIALS AND METHODS

### Populations and samples

Peripheral blood samples were collected from Tajik individuals living in Tashkurgan Village, Xinjiang Uyghur Autonomous Region, China. Each individual was the offspring of a non-consanguineous marriage of members of the same nationality within three generations. Informed consent was obtained from all individual participants included in the study. The personal identifiers of all samples, if present, were removed before sequencing and analysis. TJT samples were obtained from the Human Origin data set [22]. All procedures performed were in accordance with the ethical standards of the Responsible Committee on Human Experimentation, and have been approved by the Biomedical Research Ethics Committee of Shanghai Institutes for Biological Sciences (ER-sIBS-261408) and the Helsinki Declaration of 1975 (revised in 2000).

### Genotyping, SNP calling and quality control

We genotyped 48 Tajik samples via Affymetrix Genome-Wide Human SNP Array 6.0 and sequenced 26 Tajik samples via Illumina. Reads were merged, adaptor-trimmed and mapped to the human reference genome (GRCh37) using BWA-MEM v0.7.10 [78]. We executed duplicate mark and base quality recalibration using GATK v3.8 [79], and the missing rate of each sequenced sample was <10%. SNP genotypes from the Affymetrix Genome-Wide Human SNP array were called with ‘apt-probeset-genotype’ from Affymetrix Power Tools 1.10.2 (Affymetrix, Inc). SNP calling yielding a confidence value of <0.1 were considered missing data. We examined the potential batch effects using PCA [80,81]. To improve the power for analysis of natural selection, we performed genotype imputation using IMPUTE2 [82] with 2 phased reference panels, 1000 Genome Project reference panels [48] and our XJT-sequenced samples.

### Statistical analyses

ADMIXTURE [83] was applied to the merged data set of Human Origins [22] and XJT data. We used HAPMIX [84] to infer ancestral tracks of target populations with Mala/Vishvabrahmin and Sardinian/Basque as proxies of SAS and EUR ancestries, respectively. We further inferred the admixture time and proportion by MultiWaver 2.0 [23]. The genetic diversity was estimated using various statistics, including (θ_π_), (H), (θ_K_), proportions of rare SNVs (AF < 0.05) and Tajima's *D* statistics. The AFd_e_ from the genome-wide variation of XJT was quantified according to the absolute difference between the observed AFs and those expected in XJT. iHS was applied to detect the putative selective sweeps within XJT using Selscan (v1.3.0) [85] for the genome-wide scanning, with all the default parameters. XP-EHH analysis was performed to investigate the cross-population selective sweeps between XJT and the reference populations. SNV-level differentiation between XJT and the reference populations was measured using the *F*_ST_ following Weir and Cockerham, by VCFtools (0.1.15) [86,87], using the data set used for the XP-EHH analysis. CMS statistics [47] were constructed using four statistics, AFd_e_, iHS, XP-EHH and *F_ST_*. We identified archaic segments using ArchaicSeeker2 [54] and Sprime [55]. Archaic genomes included Altai Neanderthal and Denisovan populations. Detailed descriptions of the methods are available in the supplementary data.

## Supplementary Material

nwae284_Supplemental_File

## Data Availability

All data are available in the main text or the supplementary data. The variants of 74 XJT samples have been deposited in the Genome Variation Map (GVM, https://ngdc.cncb.ac.cn/gvm) in the National Genomics Data Center, Beijing Institute of Genomics, Chinese Academy of Sciences and China National Center for Bioinformation, under accession number GVM000554.
