# Supplementary material for: Ancestral origins and post-admixture adaptive evolution of highland Tajiks
Source: Natl Sci Rev. 2024 Aug 20;11(9):nwae284. doi: 10.1093/nsr/nwae284 (PMC11879426; doi:10.1093/nsr/nwae284)
Supplement: nwae284_Supplemental_File [file nwae284_supplemental_file.docx]

Supplementary Materials

**Ancestral origins and post-admixture adaptive evolution of highland Tajiks**

Jia Wen^1, †^, Jiaojiao Liu^1,2, †^, Qidi Feng^3, †^, Yan Lu^1,4^, Kai Yuan^3^, Xiaoxi Zhang^2,3^, Chao Zhang^3^, Yang Gao^1^, Xiaoji Wang^3^, Dolikun Mamatyusupu^5^, Shuhua Xu^1,2,3*^

^1^ State Key Laboratory of Genetic Engineering, Human Phenome Institute, Zhangjiang Fudan International Innovation Center, Center for Evolutionary Biology, School of Life Sciences, Department of Liver Surgery and Transplantation, Liver Cancer Institute, Zhongshan Hospital, Fudan University, Shanghai 200032, China;

^2^ School of Life Science and Technology, ShanghaiTech University, Shanghai 201210, China;

^3^ Key Laboratory of Computational Biology, Shanghai Institute of Nutrition and Health, University of Chinese Academy of Sciences, Chinese Academy of Sciences, Shanghai 200031, China;

^4^ Ministry of Education Key Laboratory of Contemporary Anthropology, Fudan University, Shanghai 200438, China;

^5^ College of the Life Sciences and Technology, Xinjiang University, Urumqi 830046, China

*Correspondence and requests for materials should be addressed to S.X. (Email: [xushua@fudan.edu.cn](mailto:xushua@fudan.edu.cn))

Contents

[Supplementary Materials 1](#_Toc174476856)

[Detailed Methods 4](#_Toc174476857)

[Text. S1 | Populations and samples 5](#_Toc174476858)

[Text. S2 | Genotyping, SNP calling, and quality control 5](#_Toc174476859)

[Text. S3 | Genotype imputation 5](#_Toc174476860)

[Text. S4 | Public and published data 6](#_Toc174476861)

[Text. S5 | Calculation of *F*_ST_ 6](#_Toc174476862)

[Text. S6 | PCA 7](#_Toc174476863)

[Text. S7 | Phylogenetic tree construction 7](#_Toc174476864)

[Text. S8 | Global ancestry inferred with ADMIXTURE 7](#_Toc174476865)

[Text. S9 | Detection of the admixture parameter 8](#_Toc174476866)

[Text. S10 | Post-admixture genetic diversity 8](#_Toc174476867)

[Text. S11 | Allele frequency spectrum 8](#_Toc174476868)

[Text. S12 | Genome-wide allele frequency deviation 9](#_Toc174476869)

[Text. S13 | iHS analysis of XJT 10](#_Toc174476870)

[Text. S14 | XP-EHH analysis between XJT and the reference populations 10](#_Toc174476871)

[Text. S15 | *F*_ST_ among XJT and the reference populations 10](#_Toc174476872)

[Text. S16 | CMS statistics for the detection of strong selective sweeps 10](#_Toc174476873)

[Text. S17 | Variant annotation 11](#_Toc174476874)

[Text. S18 | Gene set enrichment analysis 11](#_Toc174476875)

[Text. S19 | Detecting the archaic sequence in XJT 11](#_Toc174476876)

[Supplementary Figures 13](#_Toc174476877)

[Fig. S1 | PCA of XJT and global reference populations. 14](#_Toc174476878)

[Fig. S2 | Genetic affinities of XJT in the context of worldwide populations. 15](#_Toc174476879)

[Fig. S3 | Derived allele frequency spectrum of XJT, EUR, SAS, and EAS. 16](#_Toc174476880)

[Fig. S4 | Genetic affinities of TJT in the context of worldwide populations 17](#_Toc174476881)

[Fig. S5 | Number of SNPs at the individual level of XJT, where each point in the graph represents an individual. 18](#_Toc174476882)

[Fig. S6 | Novel SNVs in XJT. 19](#_Toc174476883)

[Fig. S7 | Allele frequency distribution of LoF mutations in XJT 20](#_Toc174476884)

[Fig. S8 | The distribution of XJT-enriched LoF variants in the worldwide populations 21](#_Toc174476885)

[Fig. S9 | The allele-C frequency of loci around rs78180793 (LoF) in XJT 22](#_Toc174476886)

[Fig. S10 | The allele frequency of protective variants in XJT and KGP dataset 23](#_Toc174476887)

[Fig. S11 | ADMIXTURE results of XJT and other worldwide populations when K is from 2 to 20. 24](#_Toc174476888)

[Fig. S12 | ADMIXTURE results of XJT with Eurasian populations when K=4 25](#_Toc174476889)

[Fig. S13 | The IBD sharing between XJT and the populations in KGP 26](#_Toc174476890)

[Fig. S14 | The number of segregating sites (/Kb) of XJT and reference populations. 27](#_Toc174476891)

[Fig. S15 | Genetic polymorphism (heterozygosity) of XJT and worldwide populations. 28](#_Toc174476892)

[Fig. S16 | Distribution of intermediate- and long Runs of homozygosity (ROH) across worldwide populations. 29](#_Toc174476893)

[Fig. S17 | Gene expression analysis of rs1127796 on *MPI* in different tissues. 30](#_Toc174476894)

[Fig. S18 | Bulk tissue gene expression for *BEST1* 31](#_Toc174476895)

[Fig. S19 | Gene expression analysis of rs1109748 on *BEST1* in different tissues. 32](#_Toc174476896)

[Fig. S20 | The correlation between gene-level CMS value and SNP number of genes. 33](#_Toc174476897)

[Fig. S21 | Gene set enrichment analysis in GO BP sub-ontology 34](#_Toc174476898)

[Fig. S22 | Gene set enrichment analysis in GO CC sub-ontology 35](#_Toc174476899)

[Fig. S23 | The candidate region (*FRAS1* and *COL5A1*) associated with skin protection 36](#_Toc174476900)

[Fig. S24 | Proportion of archaic introgression 37](#_Toc174476901)

[Fig. S25 | The allele frequency of rs79556692 (*LRRC2*) in XJT and KGP dataset 38](#_Toc174476902)

[Fig. S26 | Annotation of genes previously reported in high-altitude adaptation studies and also identified in XJT 39](#_Toc174476903)

[Fig. S27 | A candidate region associated with mean corpuscular-hemoglobin concentration (MCHC) or mean red cell distribution width (MRDW) in *ANK1* 40](#_Toc174476904)

[Fig. S28 | The frequency of ALT alleles in SNPs located within the candidate region of the *ANK1* 41](#_Toc174476905)

[Fig. S29 | Allele frequency deviation of tag SNPs in *EPAS1*, *EGLN1* for XJT. 42](#_Toc174476906)

[Fig. S30 | Allele frequency of tag SNPs in *EPAS1*, *EGLN1* for XJT, Tibetans (TBN), and other reference populations. 43](#_Toc174476907)

[Fig. S31 | PCA of sequencing and microarray data of XJT 44](#_Toc174476908)

[Supplementary Tables 45](#_Toc174476909)

[Table. S1 | Clinic-protective variants enriched in XJT. 46](#_Toc174476910)

[Table. S2 | The admixture time of Western ancestries of XJT. 47](#_Toc174476911)

[Table. S3 | The admixture time of Eastern and Western ancestries of XJT. 48](#_Toc174476912)

[Table. S4 | Pathways enriched for genes of high AFd_e_ in analysis of mGSEA. 49](#_Toc174476913)

[Table. S5 | Assignment of genes to bins. 54](#_Toc174476914)

[Table. S6 | Significant genes involved in epidermis development. 55](#_Toc174476915)

[Table. S7 | Pathways enriched for genes of high CMS value in analysis of mGSEA. 57](#_Toc174476916)

[Table. S8 | The Denisovan introgressed haplotypes significantly enriched in XJT. 58](#_Toc174476917)

[Table. S9 | The Neanderthal introgressed haplotypes significantly enriched in XJT. 59](#_Toc174476918)

[Table. S10 | The candidate genes involved in high-altitude adaptation previously reported in Ethiopians, Andeans, Tibetans, and Tibetan animals. 60](#_Toc174476919)

[Table. S11 | Significant genes in AFd_e_ analysis that are reported previously. 62](#_Toc174476920)

[Table. S12 | Significant genes in CMS analysis that are reported previously. 64](#_Toc174476921)

[Reference 65](#_Toc174476922)

# Detailed Methods

## Text. S1 | Populations and samples

Peripheral blood samples were collected from Tajik individuals (XJT) living Tashkurgan Village, Xinjiang Uyghur Autonomous Region, China. Each individual was the offspring of a non-consanguineous marriage of members of the same nationality within 3 generations. Informed consent was obtained from all individual participants included in the study. The personal identifiers of all samples, if present, were removed before sequencing and analysis. All procedures performed were in accordance with the ethical standards of the Responsible Committee on Human Experimentation, and have been approved by the Biomedical Research Ethics Committee of Shanghai Institutes for Biological Sciences (ER-sIBS-261408) and the Helsinki Declaration of 1975 (revised in 2000).

## Text. S2 | Genotyping, SNP calling, and quality control

We genotyped 48 Tajik samples via Affymetrix Genome-Wide Human SNP Array 6.0 and sequenced 26 Tajik samples via Illumina. 25 out of 26 sequenced Tajik samples were duplicated in microarray data. SNP genotypes from the Affymetrix Genome-Wide Human SNP array were called with "apt-probeset-genotype" from Affymetrix Power Tools 1.10.2 (Affymetrix, Inc). SNP calling yielding a confidence value < 0.1 was considered missing data. After the initial quality control, 46 genotyped individuals and 25 sequenced individuals were retained for further analysis. The missing rate of each sample was less than 10%. Only autosomal SNPs were used for subsequent analyses, and replicate SNPs with lower quality were removed, leaving 890,969 loci. No batch effects were observed in the principal component analysis (PCA)[1, 2] (Fig. S31). Repeat samples from genotype and sequence platforms overlapped in the PCA plot. One sequenced sample and two genotyped samples were discarded because they had 1st-degree relationships with other samples inferred by KING. Finally, 44 genotyped and 24 sequenced samples remained for further analysis, and 23 out of the 24 sequenced samples were duplicated among the genotyped samples.

## Text. S3 | Genotype imputation

To improve the power of the analysis of natural selection, we performed genotype imputation by using IMPUTE2[3] with 2 phased reference panels, 1000 Genome Project reference panels[4], and our XJT-sequenced samples. First, in the prephase study, the strains were genotyped to produce the best-guess haplotypes by Shapeit4[5]. Then impute absent genotypes with parameters: -filt_rules_l "TYPE!=Biallelic_SNP", -use_prephased_g, -merge_ref_panels, -known_haps_g, -int 5e6, -Ne 20000, -k_hap 3000 50, -buffer 250, -phase. Finally, imputed genotypes and haplotypes were produced after removing variants with reference allele frequencies of 1, and 10,756,917 SNPs remained. To ensure the quality of the imputed genotypes, we retained only loci for which the imputed and sequenced genotypes were identical in all 23 XJT samples. Therefore, 7,285,098 SNVs were retained for the subsequent analyses.

## Text. S4 | Public and published data

The Affymetrix Human Origins genotyping dataset[6] for 2,367 human samples was obtained with a signed letter permitting full data access, and was used for comparison with XJT under a global context. We used the "Simple population ID" instead of the "Verbose population ID" to assign the population identification for each individual. Overall, 49 populations from Africa (AFR), 60 from West Eurasia (EUR), 22 from South Asia (SAS), 23 from Central Asia (CAS)/Siberia (SIB), 22 from East Asia (EAS), 3 from Oceania, and 24 from America (AMR) resulted in a total of 2,345 individuals from 203 populations for use in the following analyses: ADMIXTURE, PCA, phylogenetic tree, and admixture history analyses. For the purposes of this study, only SNPs with reference sequence numbers and vendor-specified strands were used to combine the data. For analysis requiring high SNP density, sequencing data from the 1,000 Genomes Project phase 3 (KGP) dataset[4] and the Simons Genome Diversity Project (SGDP) dataset[7] were used as references in the analysis: post-admixture genetic diversity, allele frequency spectrum, allele frequency deviation, and local adaptation analysis.

## Text. S5 | Calculation of *F*_ST_

The genetic differences between populations were measured with *F*_ST_ values according to Weir and Cockerham's approximate formula[8]. SNP-specific *F*_ST_ values were calculated by VCFtools[9].

## Text. S6 | PCA

PCA was performed at the individual level using EIGENSOFT v4.2[1]. To investigate fine-scale population structure and individual genetic affinities, we performed a series of PCA by gradually removing outliers based on a plot of the first and second principal components (PCs) and re-analyzing the remaining samples based on the same set of SNPs.

## Text. S7 | Phylogenetic tree construction

We constructed a neighbor-joining tree based on an identity-by-state matrix calculated using PLINK[10]. The R function "hclust" was used for clustering, and a tree plot was constructed with the R package ggtree[11].

## Text. S8 | Global ancestry inferred with ADMIXTURE

ADMIXTURE[12] was applied to the merged dataset of Human Origins[6] and XJT data, which consisted of 2,391 (2,345 + 46) samples representing 204 populations. We used PLINK 1.07[10] to prune the original dataset with dense SNPs. After assigning an r^2^ threshold of 0.4 in every continuous window of 200 SNPs advanced by 25 SNPs (--indep-pairwise 200 25 0.4), 57,480 SNPs remained for ADMIXTURE analysis. We ran ADMIXTURE with random seeds for the merged dataset, assuming that the number of ancestral clusters (K) ranged from 2 to 20.

Since the clustering algorithm implemented in ADMIXTURE may incorporate stochastic simulation as part of the inference, independent analyses of the same data may result in slightly different results[13]. To obtain more reliable results, we replicated 10 times with different seeds for each run of ADMIXTURE assuming the same K.

To resolve the admixture ancestry of XJT, for each K, we identified the major clusters (with proportions > 5%) in XJT. For each major cluster, we identified its representative reference populations. When K ≥ 8, the major components of XJT are stabilized, and the proportions of the other components are less than 5%. To confirm the results, we ran ADMIXTURE, assuming K to be the number of major clusters, with only representative populations included in the analysis (Fig. S12).

## Text. S9 | Detection of the admixture parameter

We used HAPMIX[14] to infer ancestral tracks of target populations with Mala/Vishvabrahmin and Sardinian/Basque as proxies of SAS and EUR ancestries, respectively. Before performing this analysis, we tested a single chromosome by setting lambda (admixture time) from 20 to 200 with a step of 20, and the lambda parameter was determined when the largest log-likelihood appeared, indicating that it fit the data best. The theta parameter (admixture proportion) was set according to the relative scale of two ancestral components from the ADMIXTURE results. If two adjacent segments were derived from the same ancestry, we combined them into one segment; otherwise, the gap segment was distributed to two ancestries by the theta parameter.

MultWaver 2.0[15] can infer admixture parameters (e.g., admixture time, admixture waves, and admixture model) using the information on the length distribution of the ancestral tracks. We performed the analysis with the default parameters of MultiWaver 2.0.

## Text. S10 | Post-admixture genetic diversity

We analyzed the genetic diversity of XJT and the reference populations to understand the genetic diversity of the admixed population. Estimators, including nucleotide diversity (θ_π_), haplotype diversity (H), normalized numbers of segregating sites (θ_K_), proportions of rare SNVs (AF < 0.05), and Tajima’s D statistics were employed. An equal number of samples were chosen from each population to avoid bias caused by the sample size. We divided the whole genome into 50 kb windows and steps of 25 kb. The statistics θ_π_, H and θ_K_ were computed by the software “Theta_D_H.Est"[16].

## Text. S11 | Allele frequency spectrum

We investigated the site-frequency-spectrum (SFS) of XJT and other reference populations. The frequency profiles were further compared based on the 2-dimensional (2D) SFS. We estimated the expected allele frequencies (AF_exp_) of XJT by averaging those in the surrogate ancestral populations, weighted by the global admixture proportions. The results showed that the observed frequency profile of XJT was similar to that expected and closely related to that of the reference populations.

## Text. S12 | Genome-wide allele frequency deviation

We calculated the allele frequency deviation from expectation (AFd_e_) for genome-wide SNVs of XJT as the absolute difference between the observed allele frequency (AF_obs_) and expected allele frequency (AF_exp_). That is, AFd_e_ = | AF_obs_–AF_exp_ |. The expected allele frequency was estimated as the admixture-proportion-weighted average of the frequencies in ancestral populations. The weights were assigned as the global admixture proportions estimated by ADMIXTURE analysis. The significance of AFd_e_ for each SNV was further investigated according to the AFd_e_ rank. To eliminate the potential effect of the minor allele frequency (MAF), we grouped all the variants according to their expected MAF into bins of size 0.01. Moreover, the potential influence of the allele frequency difference between the reference populations (AF_EUR-SAS_) was also under control. Variants were binned into ranges of AF_EUR-SAS_ [0.0,0.01), [0.01,0.02), [0.02,0.03), [0.03,0.04), [0.04,0.05), [0.05,0.06), [0.06,0.10), [0.10,0.15), [0.15,0.20), [0.20,0.30), and [0.30,1.0], which also ensured sufficient variants within each bin. Next, the empirical *P*-value for each SNV was estimated within the corresponding bin as the percentage of SNVs with a larger AFd_e_ value. Furthermore, the autosomal region of the merged dataset was scanned using a 50 kb sliding window with a step size of 25 kb, and the proportions of SNVs with large AFd_e_ (empirical *P* < 0.01) in each window were calculated. Then, we grouped all the windows by variant counts into bins of size 10. Windows with < 60 SNVs were grouped together, as were those with > 220 SNVs, to ensure sufficient windows within each bin. We obtained 18 bins with variant counts ranging from 60 to 220 advanced by 10. The empirical *P*-value for each window was estimated within the corresponding bin as the percentage of windows with a larger proportion of large AFd_e_ SNVs. Gene annotations, including the gene boundary, were accessed from Ensembl (v96) using the R package “biomaRt”[17]; for genes with multiple windows, we retained only the one with the smallest empirical *P*-value. Genome-wide genes were ranked according to their AFd_e_ quantiles. We then used the mGSEA method[16, 18] to investigate the AFd_e_ of pathway genes.

## Text. S13 | iHS analysis of XJT

iHS was applied to detect putative selective sweeps within XJT (24 samples, 4,782,812 SNVs after filtration with MAF 0.05) by using Selscan (v1.3.0)[19] for genome-wide scanning with all the default parameters. SNVs with uncertain ancestral alleles were removed, and 4,782,811 SNVs were left for this analysis.

## Text. S14 | XP-EHH analysis between XJT and the reference populations

XP-EHH analysis was performed to investigate cross-population selective sweeps between XJT and the reference populations. We randomly selected 24 samples from each reference population, the same sample size as that for XJT, to remove the potential sample size bias. The MAF threshold was set to 0.05 for all samples, and 9,890,996 SNVs retained. Selescan (v1.3.0) was used with all default parameters to calculate the XP-EHH score of SNVs between XJT and the reference populations. Then, we weighted the XP-EHH score by the admixture proportion estimated by the ADMIXTURE analysis. XP_XJT-REF_ was obtained for 5,020,919 SNVs in total.

## Text. S15 | *F*_ST_ among XJT and the reference populations

SNV-level differentiation between XJT and reference populations was measured using the *F*_ST_ following Weir and Cockerham by VCFtools (0.1.15)[8, 9] using the dataset used for the XP-EHH analysis (9,890,996 SNVs).

## Text. S16 | CMS statistics for the detection of strong selective sweeps

Composite of multiple signals (CMS) statistics[20] were constructed using four statistics, AFd_e_, iHS, XP-EHH, and *F*_ST_. For each SNV, the following calculation was performed: CMS = –log10($\prod_{i=1}^{n} Pi$), where $Pi$ is the rank of the variant using method *i* sorted by increasing value divided by the total number of variants in method *i*.

As protein-coding genes associated with selected sweeps were a substantial focus of this analysis, we aimed to identify significant genes. First, gene-level CMS value was defined as the highest single-SNP CMS value from each gene region (including the coding region and 5 kb up/downstream of a gene). Second, to correct for potential bias caused by differences in SNP number and gene length (gene length is highly related to SNP number), the significance of each gene was assessed by comparing the gene-level CMS value with the empirical distribution of CMS values from genes with similar SNP numbers. Therefore, 18,356 protein-coding genes were assigned to 17 bins with a similar number of genes (~1,000 genes per bin), and a total of 923 significant genes were obtained (empirical *P*-value < 0.05 per bin).

## Text. S17 | Variant annotation

The variant effect was predicted by the Perl script of Ensemble Variant Effect Predictor (VEP) v87 offline with default parameters. In addition, the Combined Annotation Dependent Depletion (CADD) score[21], PhastCons score[22] and GERP++ (Genomic Evolutionary Rate Profiling) score[23] were obtained by searching downloaded files from the corresponding website with the "--custom" parameter in the VEP script. The Plugin modules, LoFtool, LoF, and Condel were also inserted to annotate SNVs by using the "--plugin" parameter.

## Text. S18 | Gene set enrichment analysis

To analyze the functional profiles (KEGG and GO) and disease associations of genes potentially subjected to selective constraints, we used clusterProfiler[24] and DOSE[25] to perform enrichment and annotation analysis in R (3.4.1) by enrichGO, enrichKEGG, and enrichDO functions, in which "pvalueCutoff" and "pAdjustMethod" were 0.05 and “BH”, respectively.

Additionally, to detect small-effect allele contributions, we also ranked genome-wide genes according to their AFd_e_/CMS value quantiles. We then used the mGSEA[16, 18] method to identify significant pathways and associated genes.

## Text. S19 | Detecting the archaic sequence in XJT

We identified archaic segments using ArchaicSeeker2[26] and Sprime[27] in this study. Archaic genomes included Altai Neanderthal and Denisovan populations. We estimated the total content of the two archaic sequences globally and searched for any archaic segments locally. Next, we designed a method to construct an expected archaic segment frequency spectrum by using the formula $AFexpect=\sum_{ref=i}^{n} Pi\cdot AFi$ for each archaic segment. *Pi* refers to the proportion of reference population *i* in XJT estimated by ADMIXTURE analysis. *AFi* represents the allele frequency of reference population *i*. We compared the differences between the expected and observed frequencies per segment in XJT and calculated the statistic D = (AF_expect_ – AF_observed_)/AF_expect_. Next, four filters were applied to obtain significant Neanderthal/Denisovan segments: 1) the archaic segment > 15 kb; 2) the segment that could be mapped to the gene; 3) the D value of the segment in the top 1% of the whole genome; and 4) the frequency of the segment in XJT ≥ 10/52 (sample size *2).

We also detected adaptive archaic introgression by calculating U and Q95[28]. U_AFR,XJT,Denisova,Neanderthal_(1%, 20%, *x*, *y*) were calculated as the number of sites where Denisova has a particular allele at frequency *x* and Altai Neanderthal has that allele at frequency *y*. In addition, the same allele should be at a frequency smaller than 1% in the AFR and larger than 20% in XJT. Then, Q95 was calculated as the 95^th^ percentile of derived frequencies in XJT of all SNPs with a derived allele frequency *x* in Denisova and derived allele frequency *y* in Altai Neanderthal, but where the derived allele is at a frequency smaller than 1% in AFR. We computed U and Q95 in 40 kb nonoverlapping windows along the genome to determine the adaptive pressure behind the putative archaic adaptive introgression. We analyzed the above statistics in three ways: 1) to identify Denisova-specific introgression, we set x = 100% and y = 0%; 2) to identify Altai Neanderthal-specific introgression, we set x = 0% and y = 100%; and 3) to identify introgressed tracts matching both Denisova and Altai Neanderthal, we set x = 100% and y = 100%.

# Supplementary Figures

**Fig. S1 | PCA of XJT and global reference populations**. Geographical regions where the individuals are located are indicated with colors, as shown in the legend. The number in the bracket represents the variance explained by each PC accounting for the top 10 PCs.


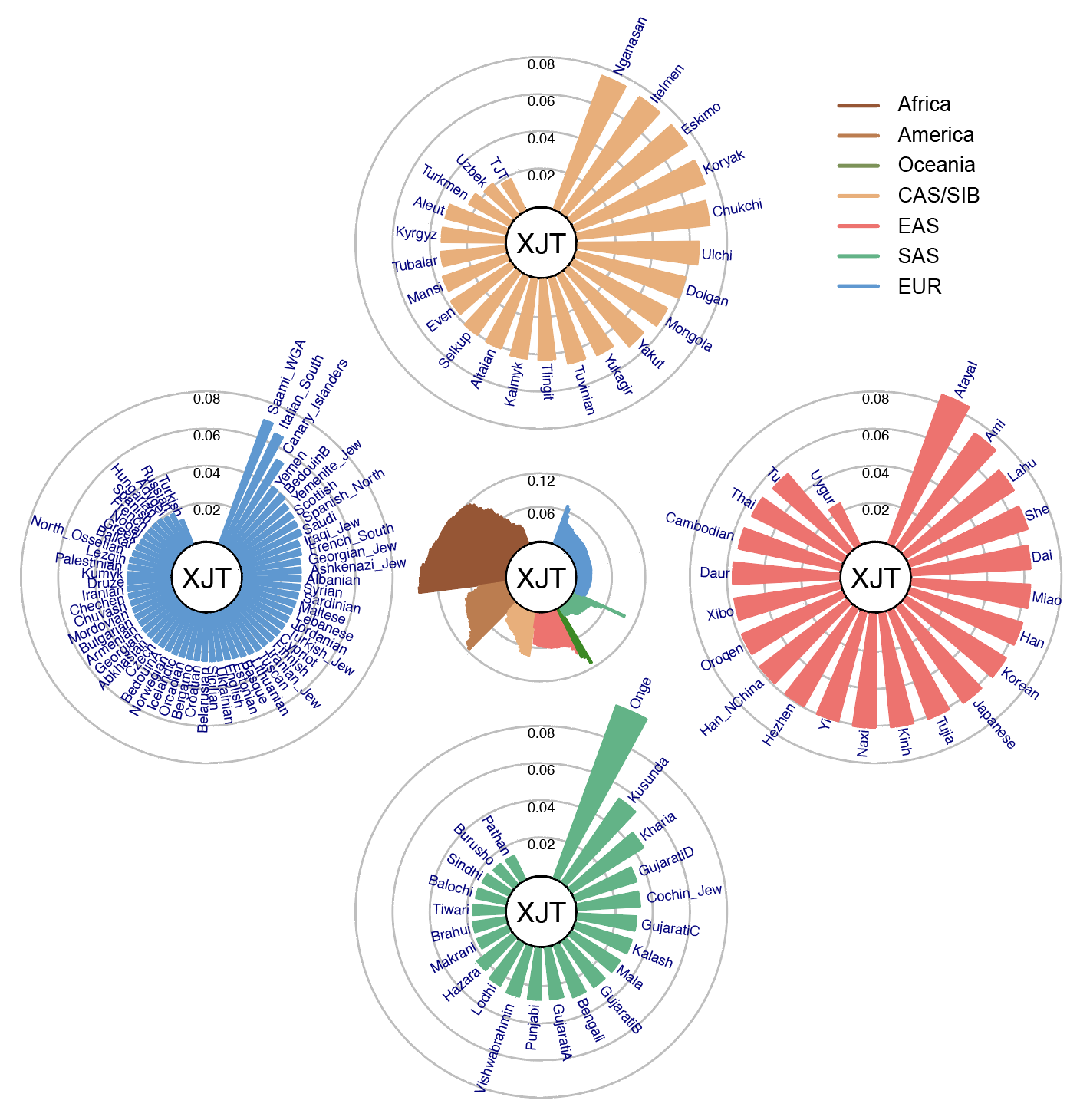


## Fig. S2 | Genetic affinities of XJT in the context of worldwide populations.

In the middle, a fan-like chart shows genetic differences (*F*_ST_) between XJT and worldwide populations. Each branch represents a comparison between XJT and 1 of the 203 populations, and the length is proportional to the *F*_ST_ value as indicated by gray circles. The area of a bar is for presentation purpose, which is not linearly proportional to genetic distance. The populations are classified by geographical regions and indicated with colors, as shown in the legend. On the top, a fan-like chart showing *F*_ST_ between XJT and Central Asian/Siberian populations. To the left, a fan-like chart showing *F*_ST_ between XJT and western Eurasian populations. At the bottom, a fan-like chart showing *F*_ST_ between XJT and South Asian populations. To the right, a fan-like chart showing *F*_ST_ between XJT and East Asian populations.


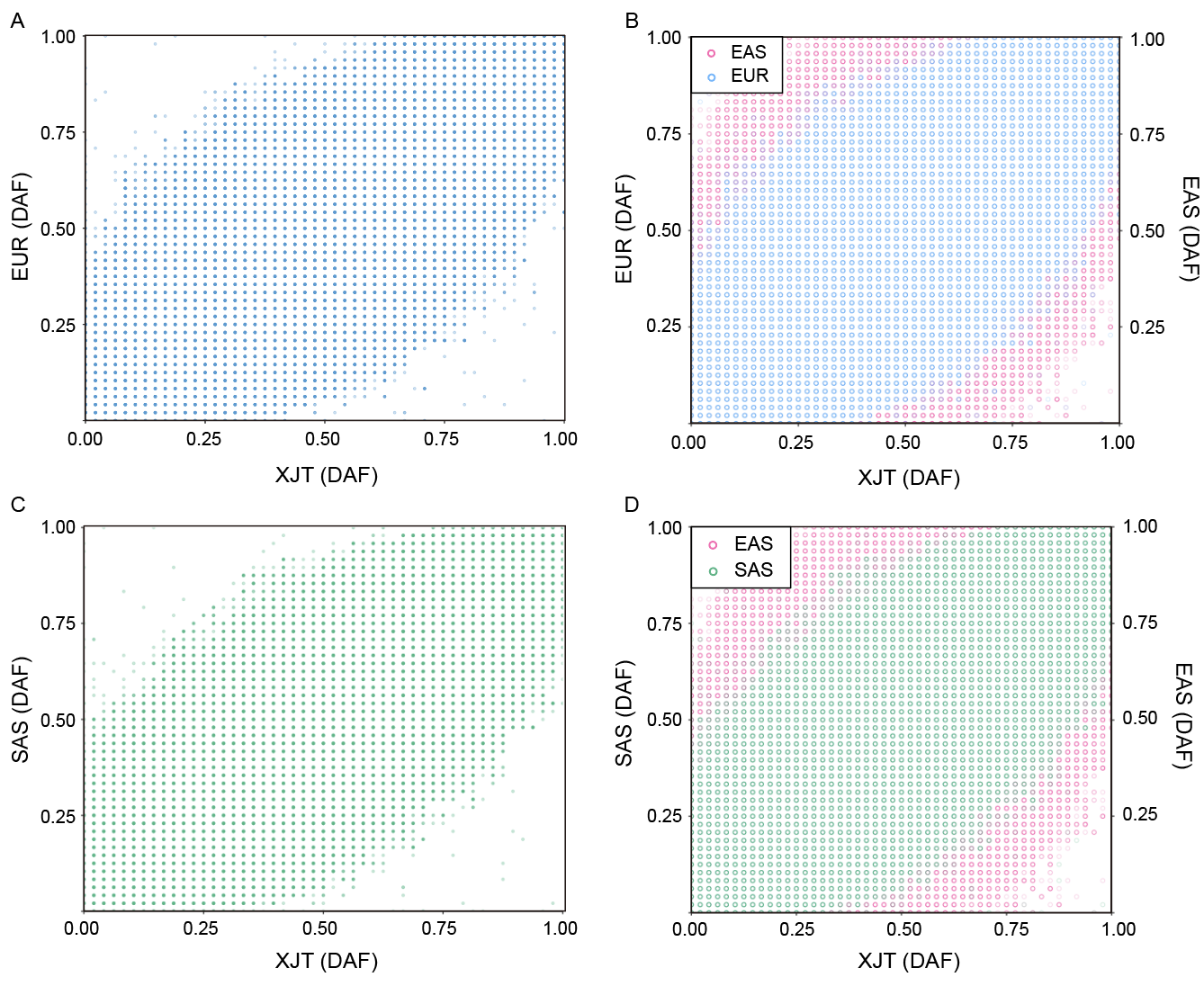


## Fig. S3 | Derived allele frequency spectrum of XJT, EUR, SAS, and EAS.


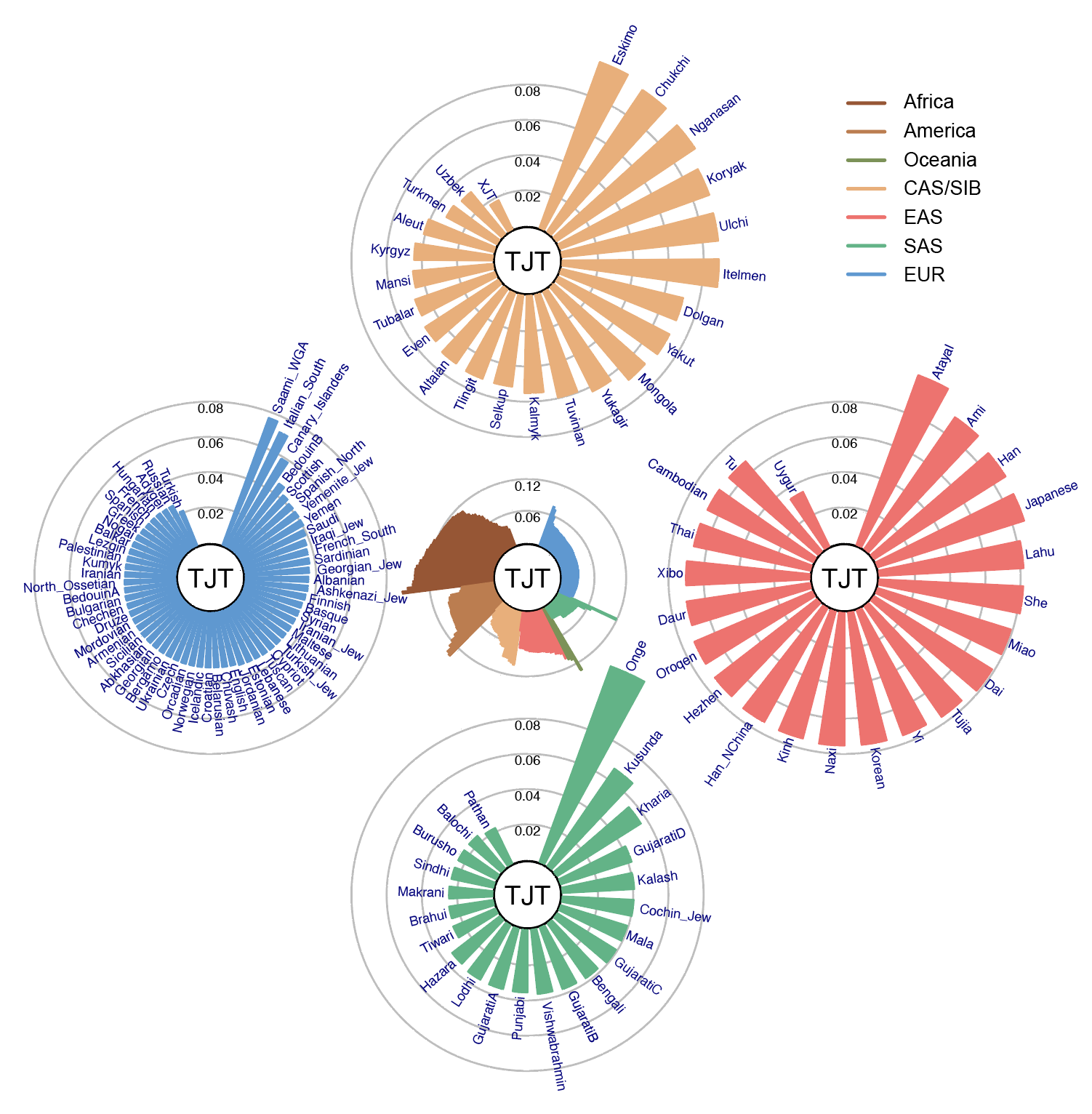


**Fig. S4 | Genetic affinities of TJT in the context of worldwide populations**.

In the middle, a fan-like chart shows genetic differences (*F*_ST_) between TJT and worldwide populations. Each branch represents a comparison between TJT and other populations, and the length is proportional to the *F*_ST_ value as indicated by gray circles. The populations are classified by geographical regions and indicated with colors, as shown in the legend. On the top, a fan-like chart showing *F*_ST_ between TJT and Central Asian/Siberian populations. To the left, a fan-like chart showing *F*_ST_ between TJT and western Eurasian populations. At the bottom, a fan-like chart showing *F*_ST_ between TJT and South Asian populations. To the right, a fan-like chart showing *F*_ST_ between TJT and East Asian populations.


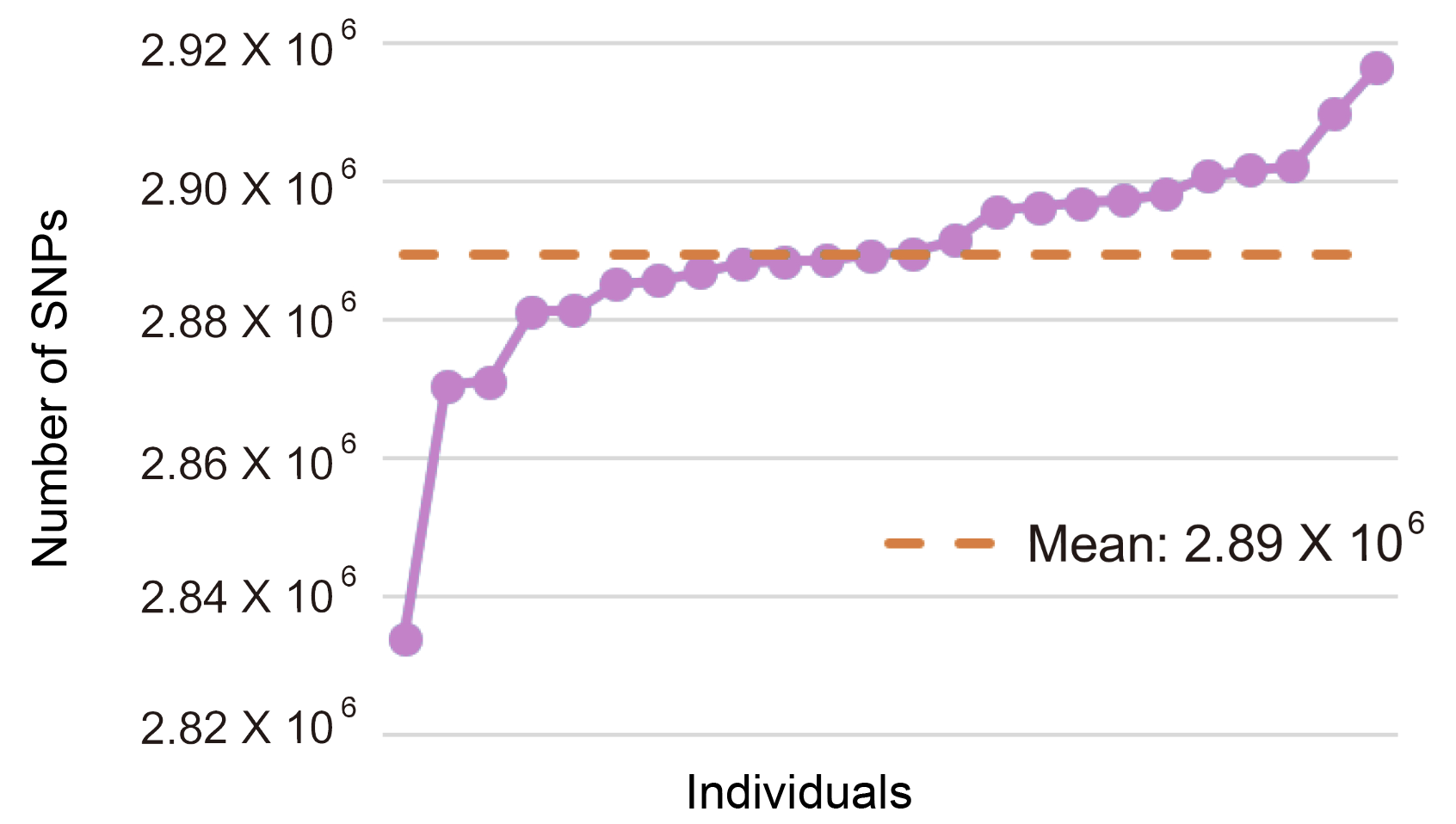


## Fig. S5 | Number of SNPs at the individual level of XJT, where each point in the graph represents an individual.


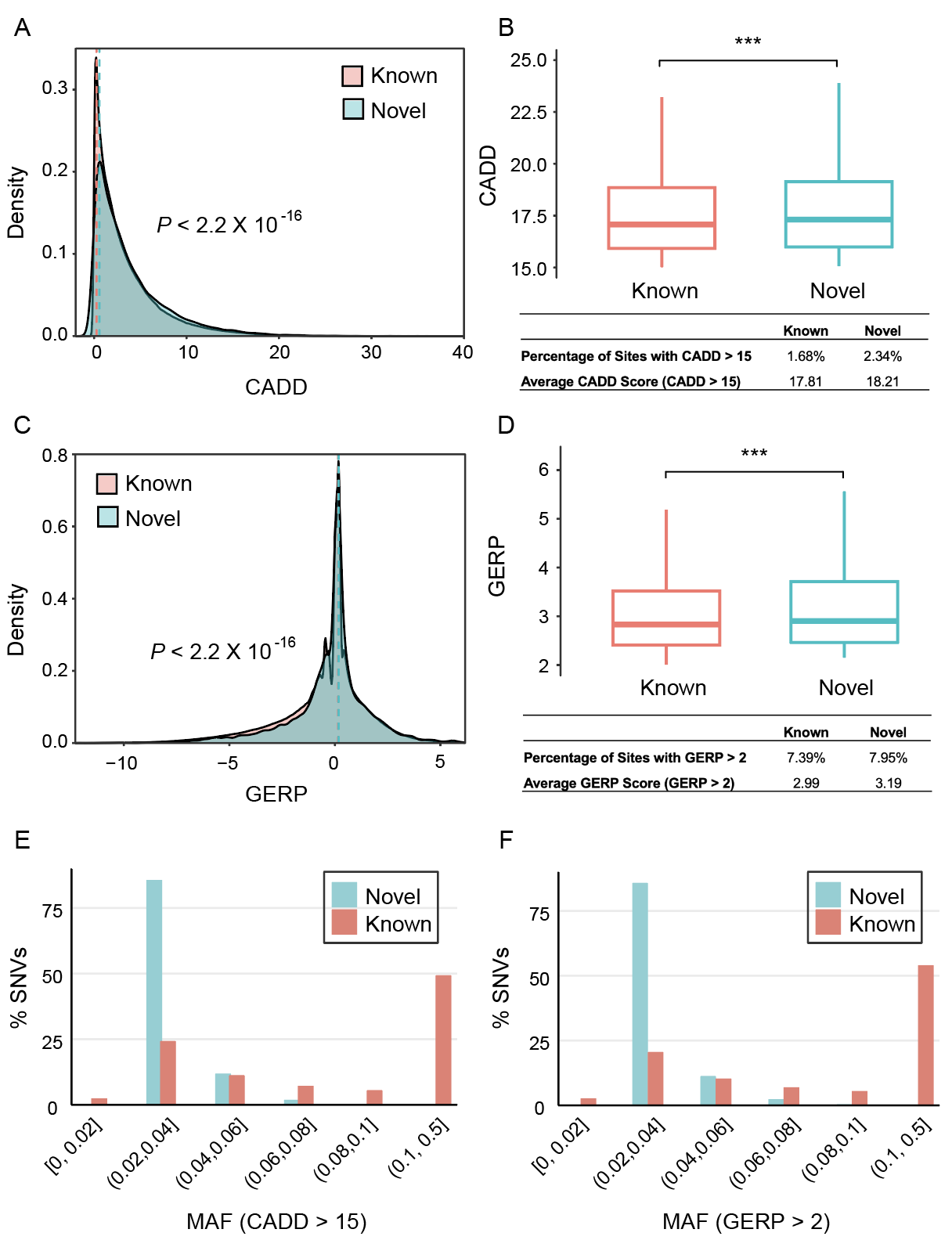


## Fig. S6 | Novel SNVs in XJT.

A. CADD score density of the known (dbSNP 155) and novel SNVs; B. CADD score (CADD > 15) distribution and statistics of the known and novel SNVs. ***：*P* < 0.001. C. GERP score density of the known and novel SNVs. D. GERP score (GERP > 2) distribution and statistics of the known and novel SNVs. ***：*P* < 0.001. MAF spectrum for E. SNVs with CADD score > 15, and F. SNVs with GERP score > 2. Proportions were estimated within MAF bins of size 0.02.

CADD > 15 and GERP > 2 respresent functionally important sits.


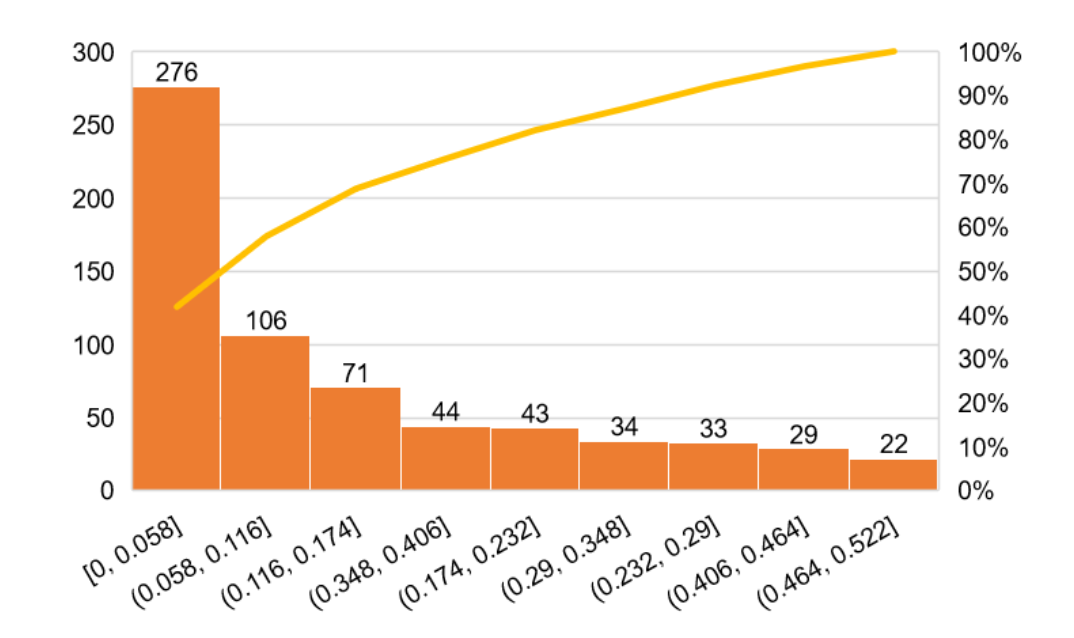


**Fig. S7 | Allele frequency distribution of LoF mutations in XJT**.


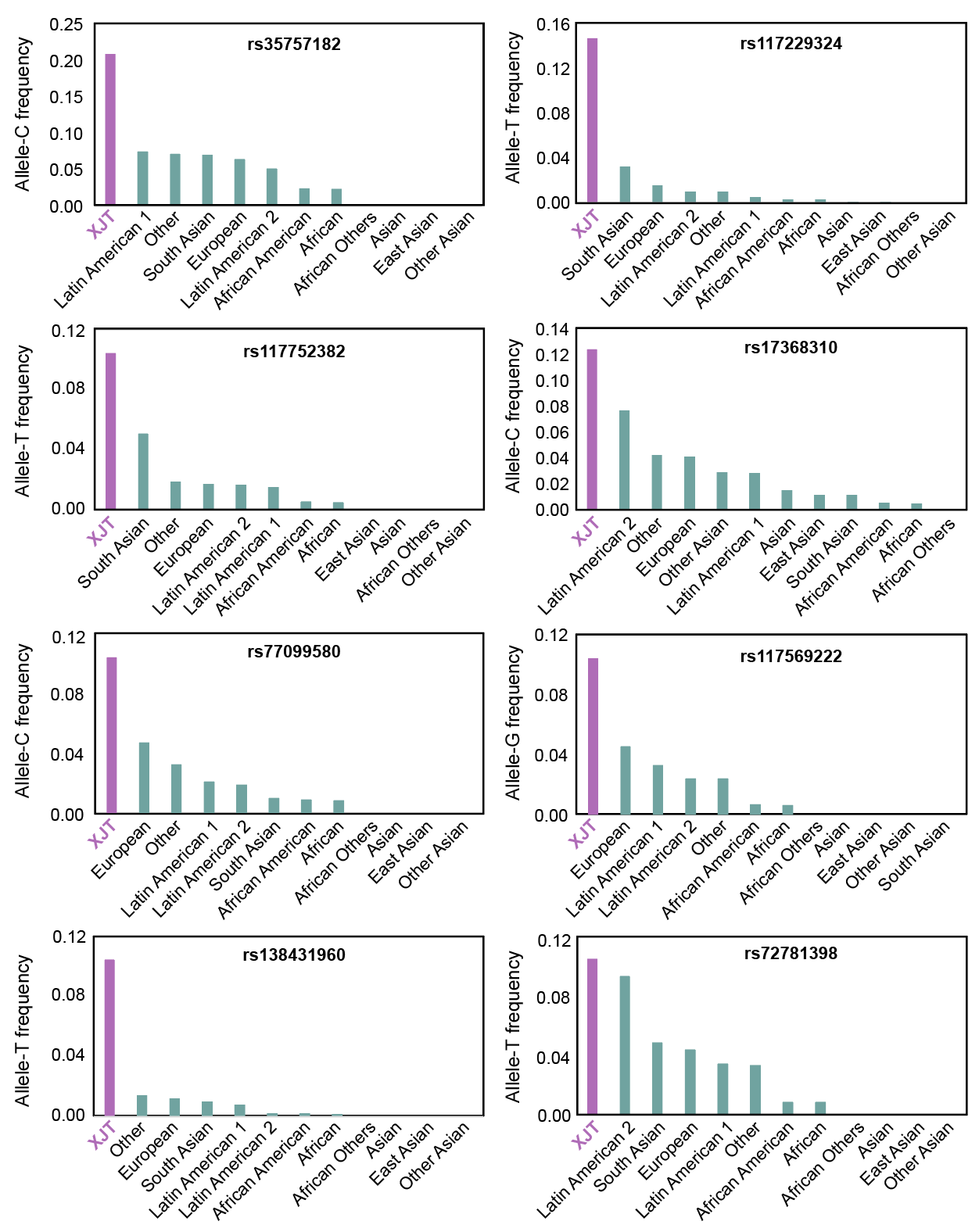


**Fig. S8 | The distribution of XJT-enriched LoF variants in the worldwide populations**.

The allele frequency was obtained via dbSNP (<https://www.ncbi.nlm.nih.gov/snp/>).


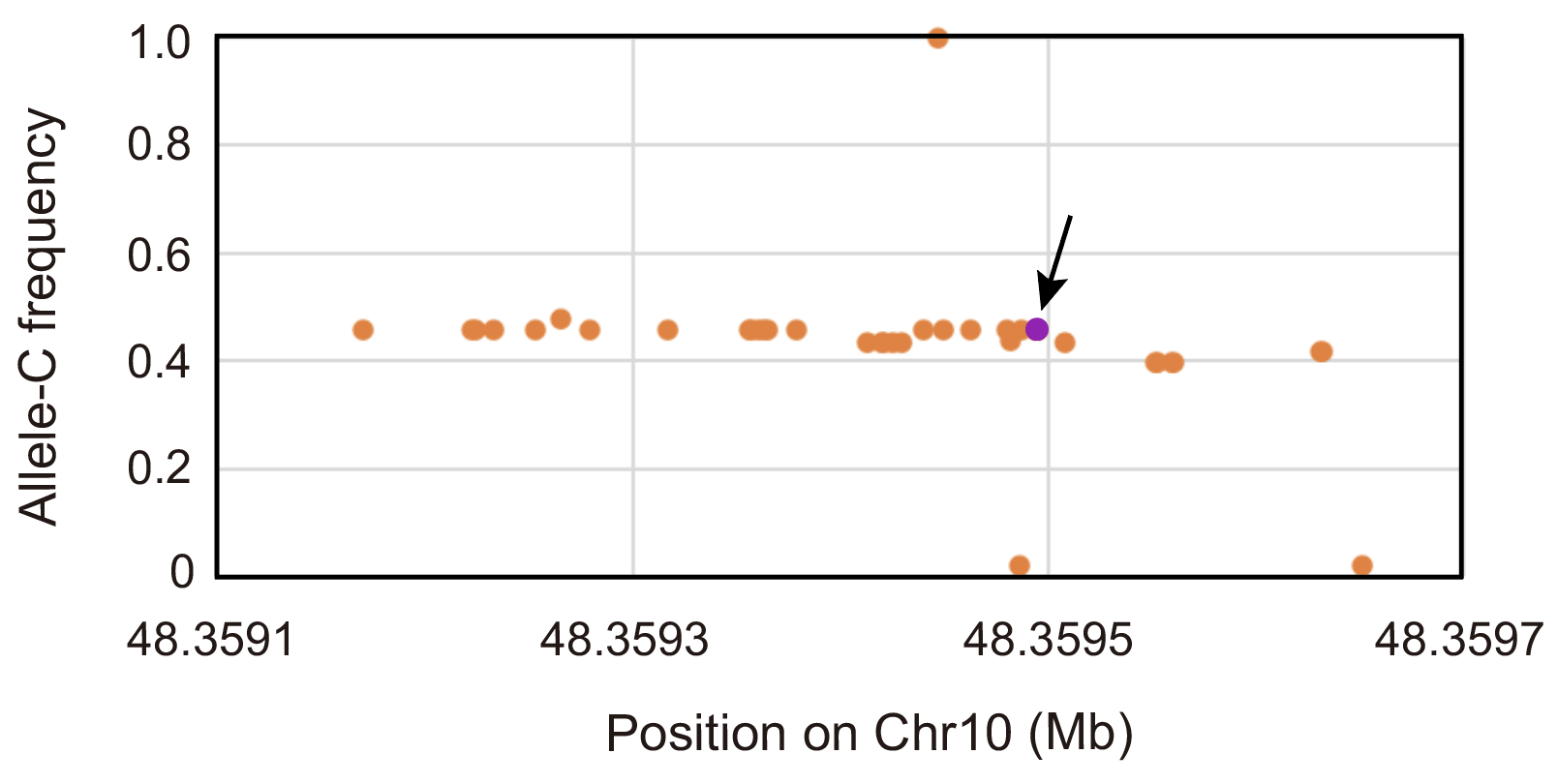


**Fig. S9 | The allele-C frequency of loci around rs78180793 (LoF) in XJT**.

The purple dot indicates rs78180793.


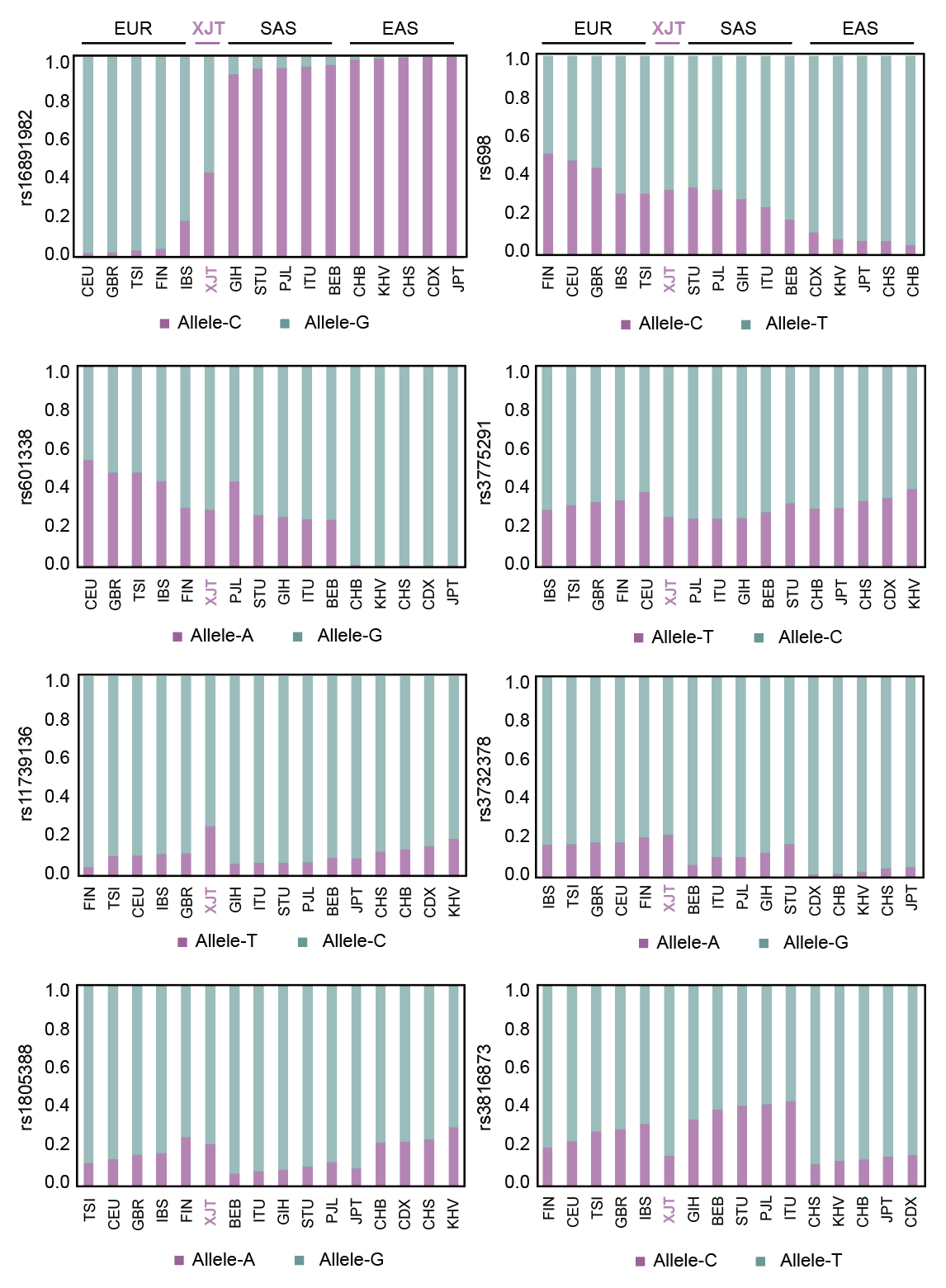


**Fig. S10 | The allele frequency of protective variants in XJT and KGP dataset**.

_­­
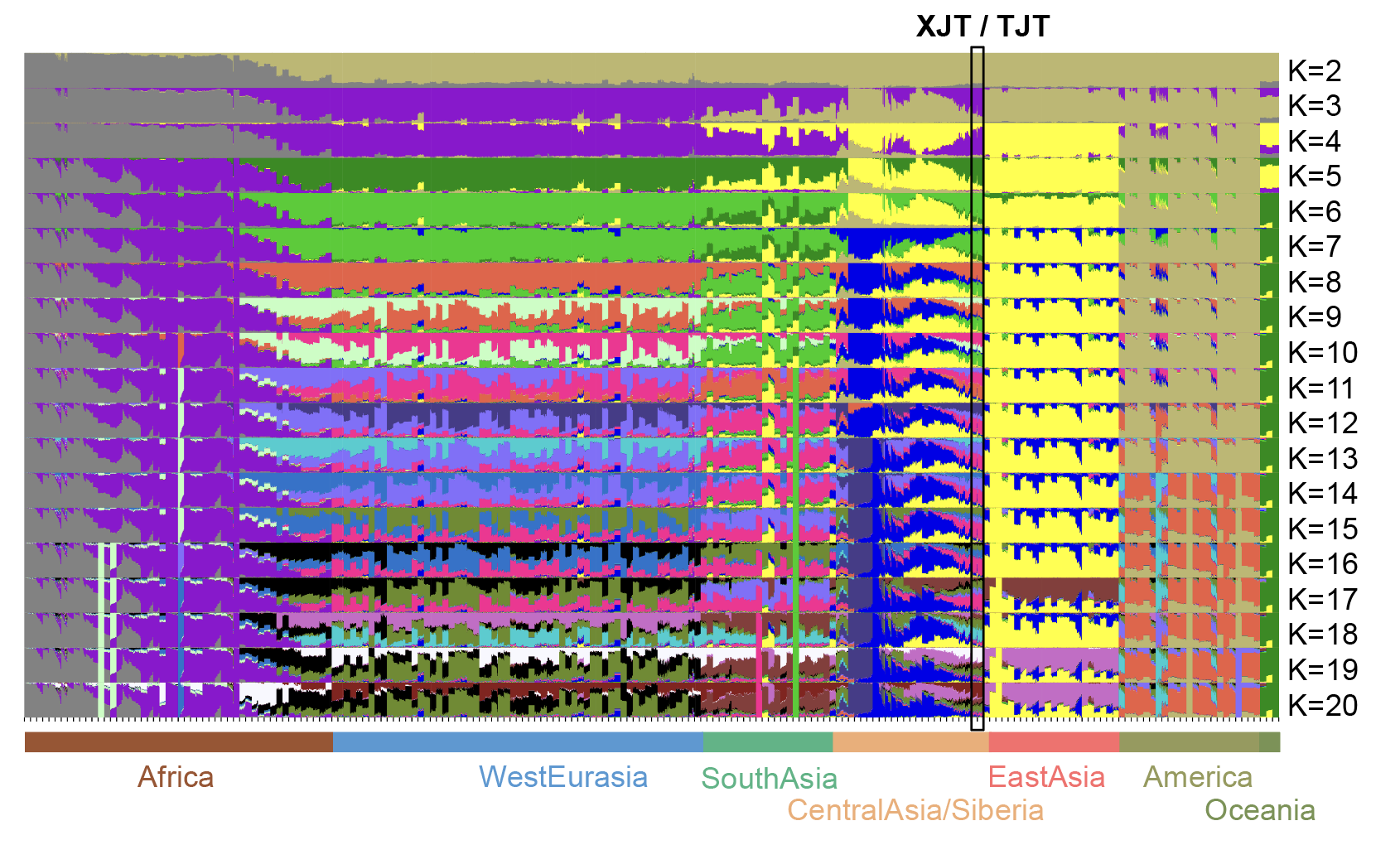
_

**Fig. S11 | ADMIXTURE results of XJT and other worldwide populations when K is from 2 to 20.**


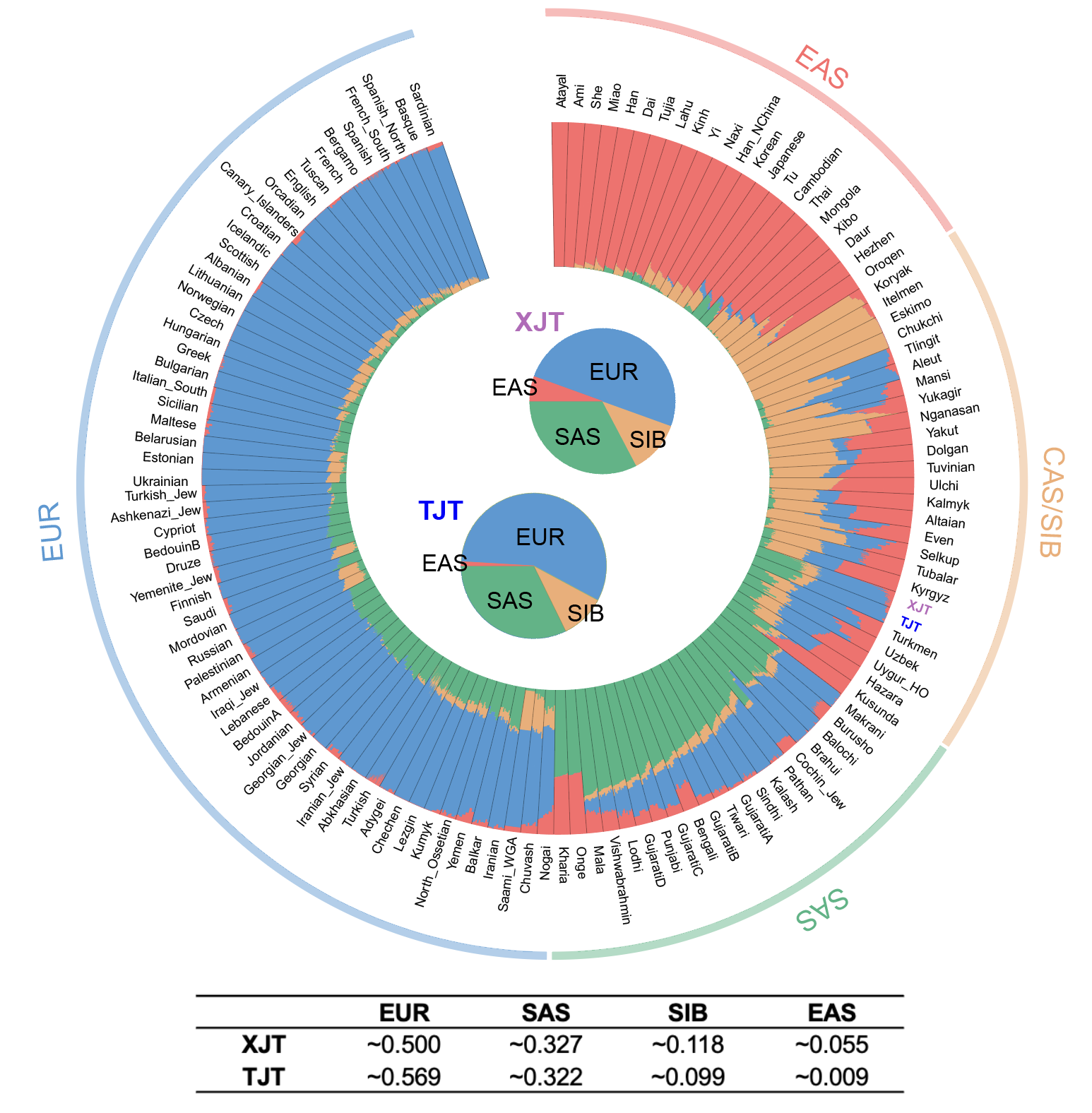


**Fig. S12 | ADMIXTURE results of XJT with** **Eurasian populations when K=4**.

The proportion of each ancestry in XJT and TJT was highlighted with a pie chart.


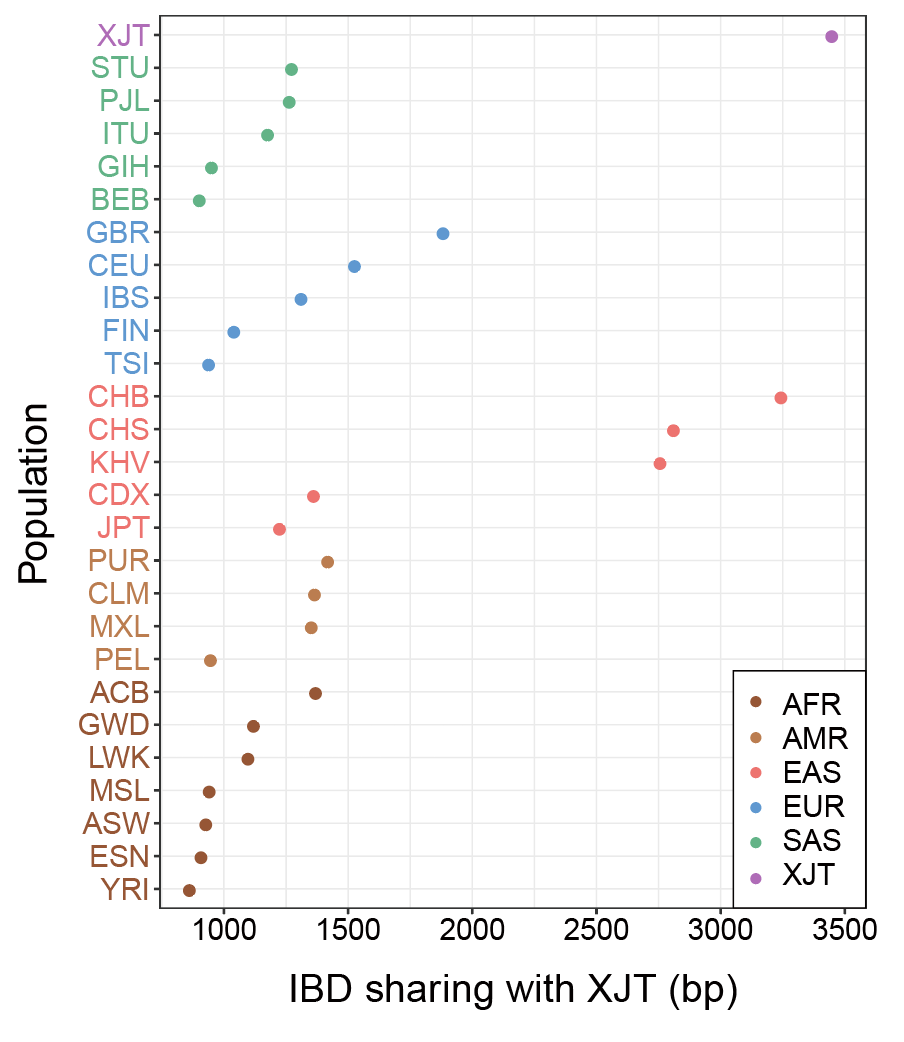


**Fig. S13 | The IBD sharing between XJT and the populations in KGP**.

Admixture model of western ancestries of XJT. X-axis indicates an average of IBD sharing per pair of samples. Populations in different regions were shown in different colors and the average IBD sharing per pair within XJT was shown by a purple dot.

﻿
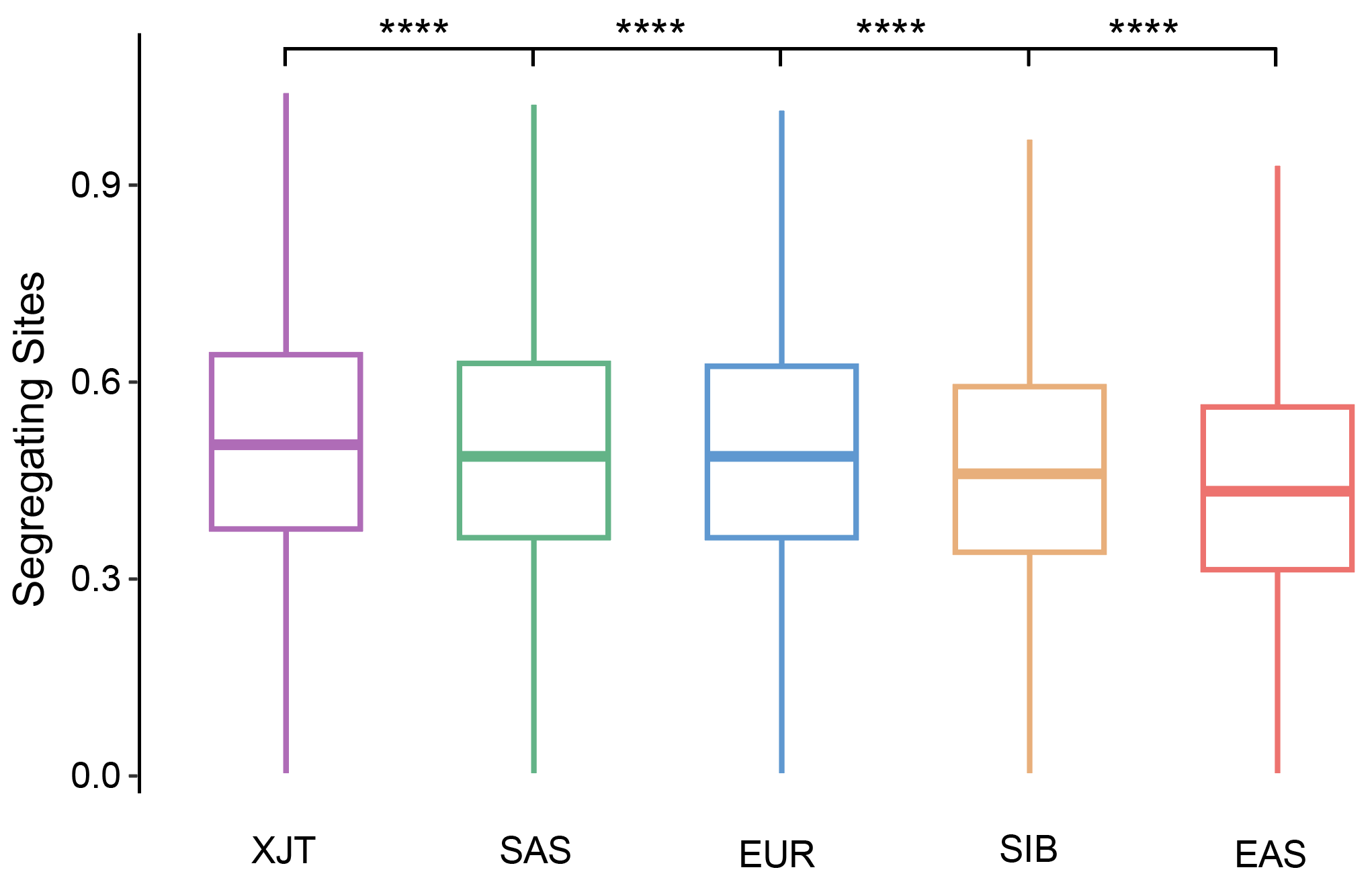


## Fig. S14 | The number of segregating sites (/Kb) of XJT and reference populations.


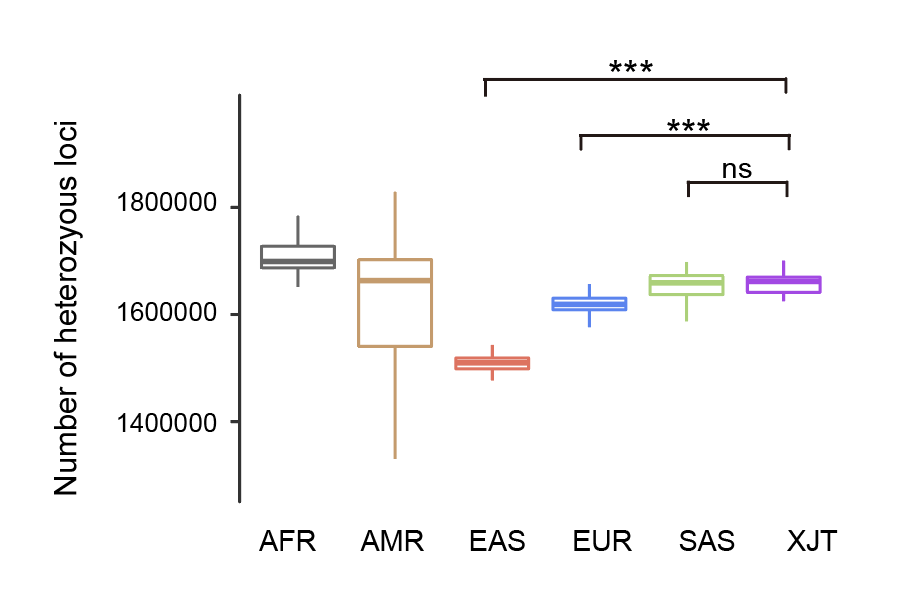


## Fig. S15 | Genetic polymorphism (heterozygosity) of XJT and worldwide populations.


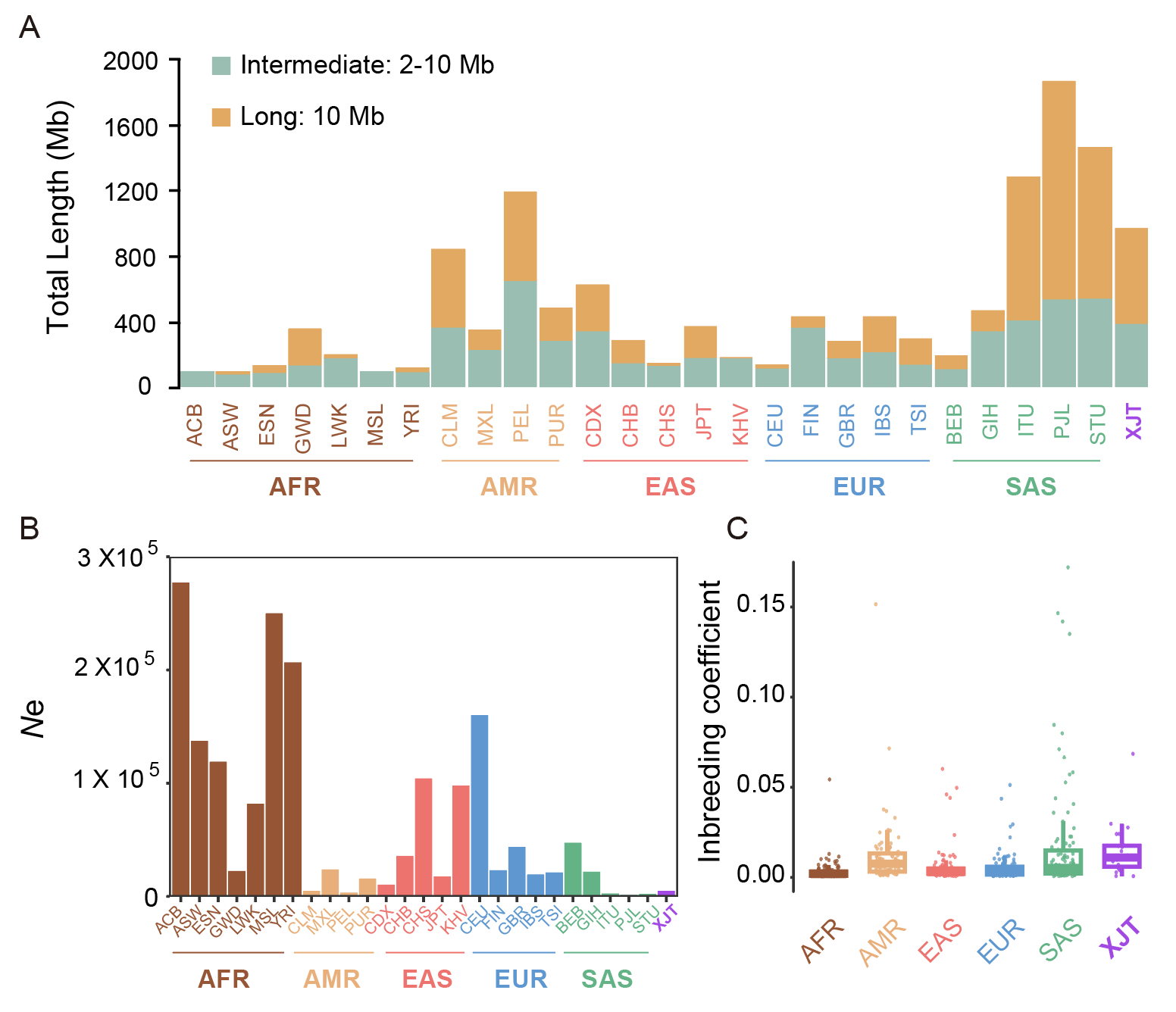


## Fig. S16 | Distribution of intermediate- and long Runs of homozygosity (ROH) across worldwide populations.

**A.** Total length of intermediate- and long ROH for XJT and worldwide populations. **B.** Estimated effective population sizes (*N*e) of XJT and worldwide populations based on ROH distribution. **C.** Estimated inbreeding coefficient of XJT and other populations based on ROH distribution.


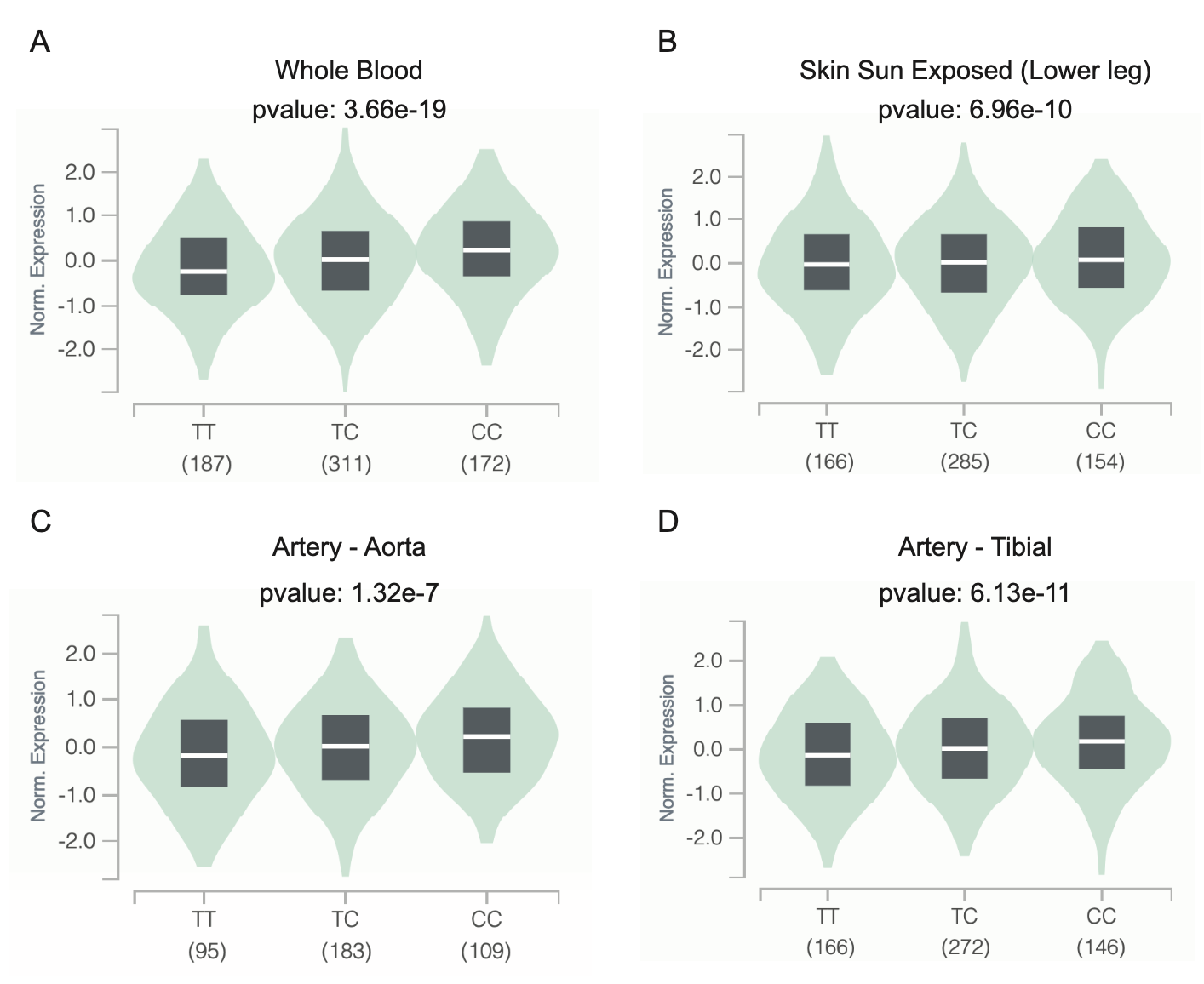


## Fig. S17 | Gene expression analysis of rs1127796 on *MPI* in different tissues.

The figures were conducted using the GTEx eQTL Calculator (<https://gtexportal.org/home/testyourown>).

**
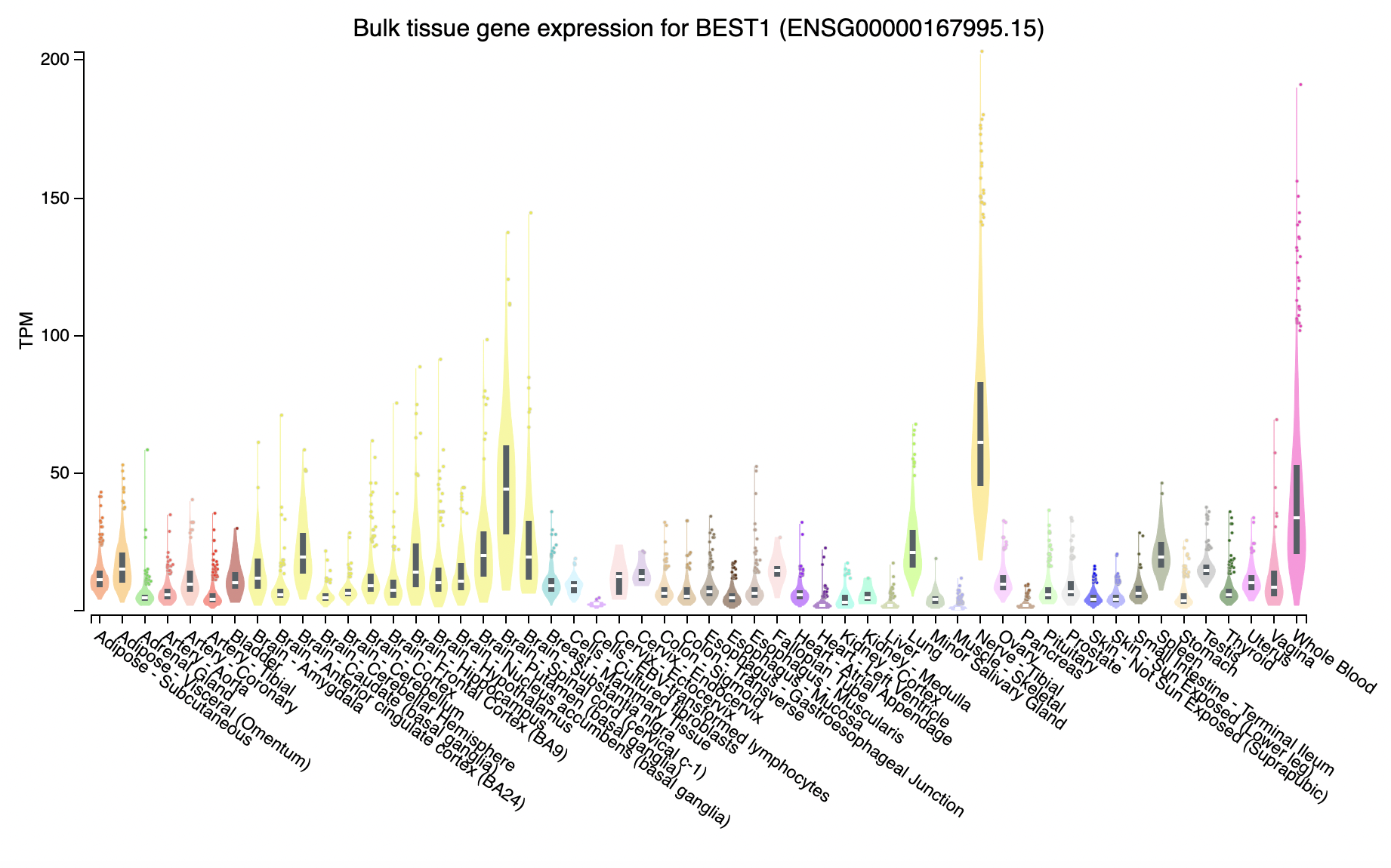
**

Fig. S18 | Bulk tissue gene expression for *BEST1* **(GTEx Analysis Release V8)**.

The x-axis represents different tissues and y-axis represents gene expression level in Transcripts Per Million (TPM). The *BEST1* was most abundantly expressed in the brain, nerve, and whole blood.


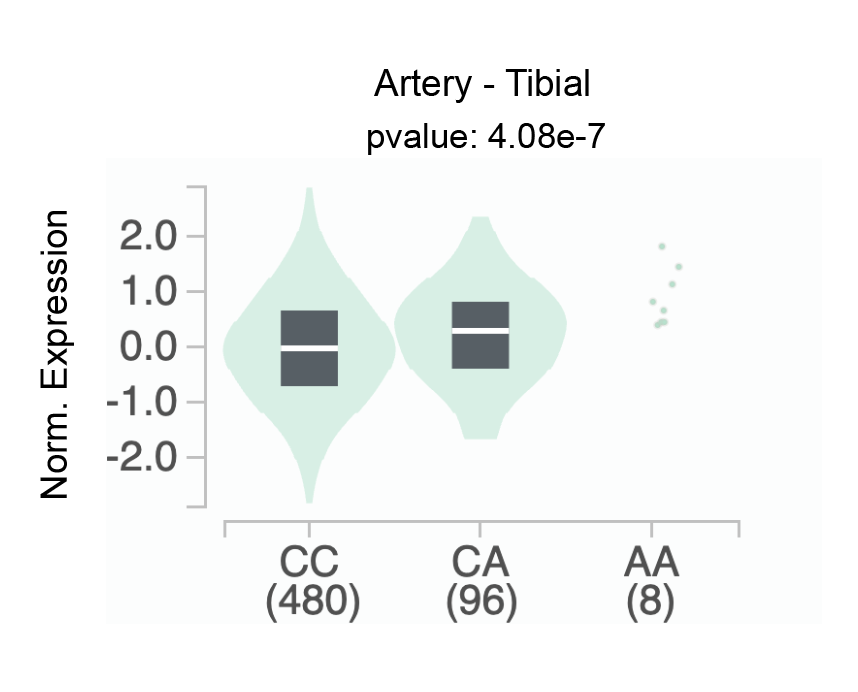


## Fig. S19 | Gene expression analysis of rs1109748 on *BEST1* in different tissues.

The figures were conducted using the GTEx eQTL Calculator (<https://gtexportal.org/home/testyourown>).


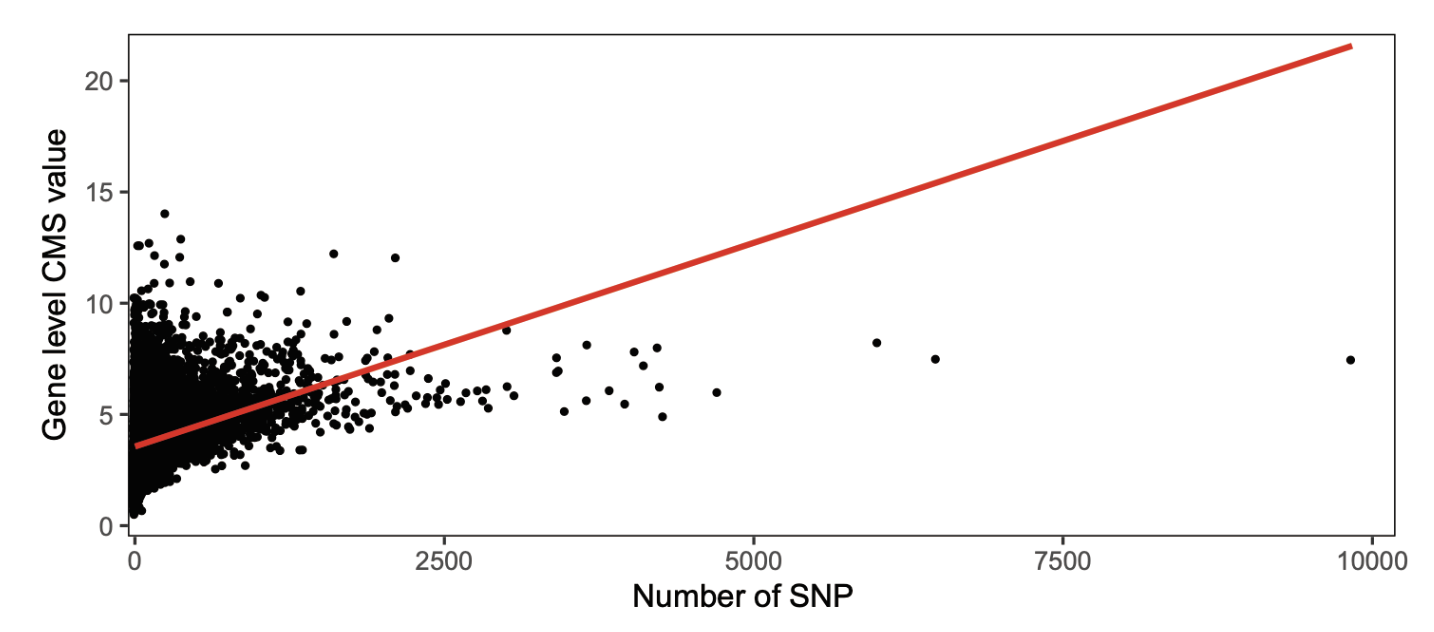


## Fig. S20 | The correlation between gene-level CMS value and SNP number of genes.

Each dot is a protein-coding gene.


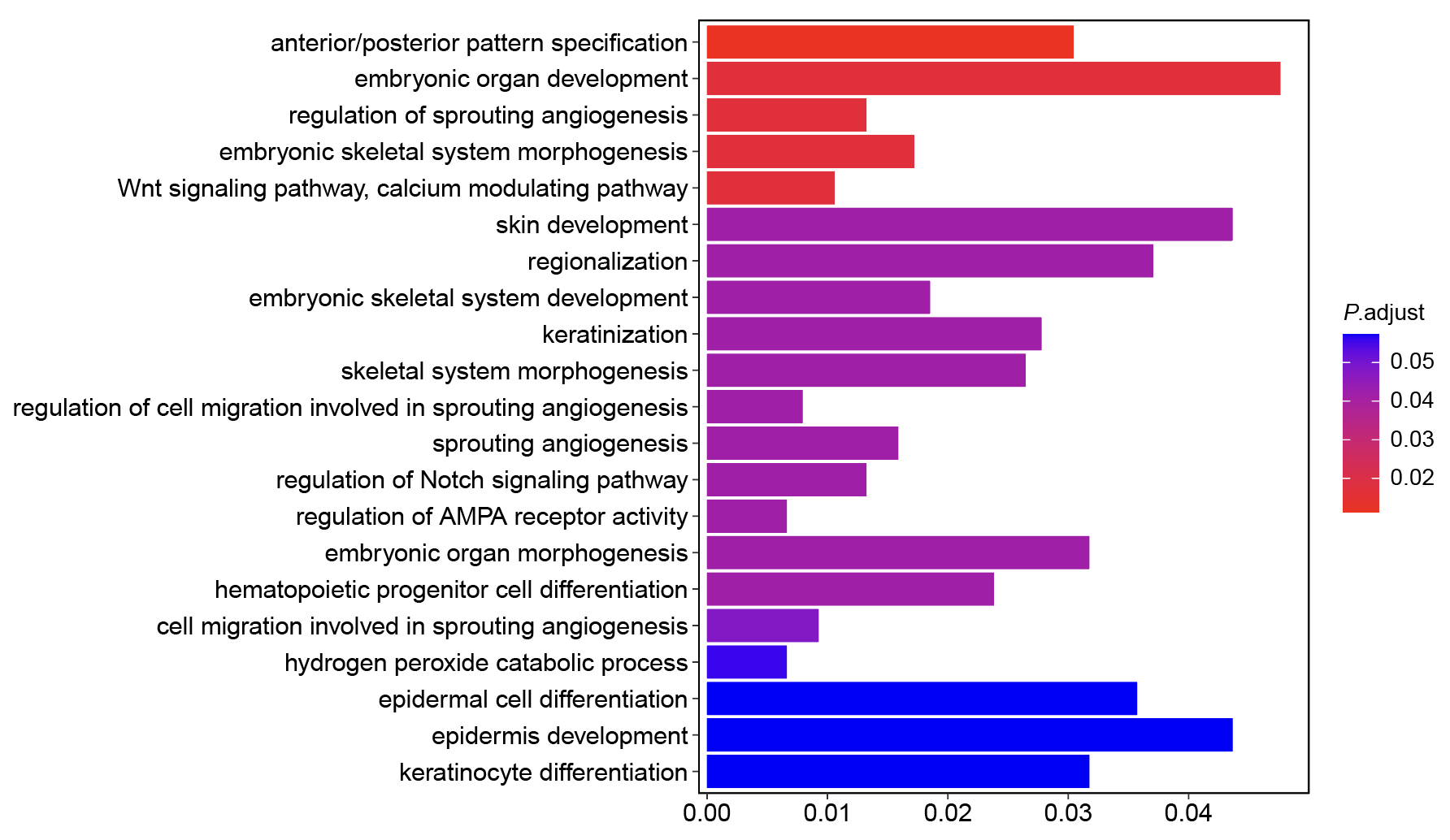


**Fig. S21 | Gene set enrichment analysis in GO BP sub-ontology**.

Adjusted *P* value increased when red became blue gradually.


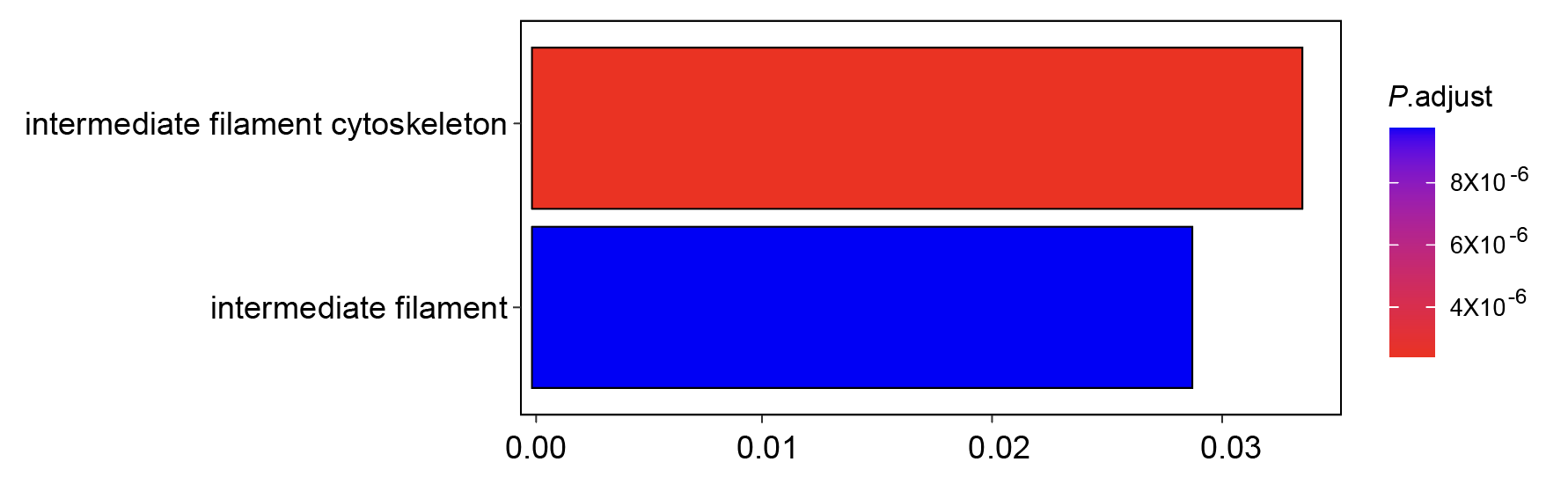


**Fig. S22 | Gene set enrichment analysis in GO CC sub-ontology**.

Adjusted *P* value increased when red became blue gradually.


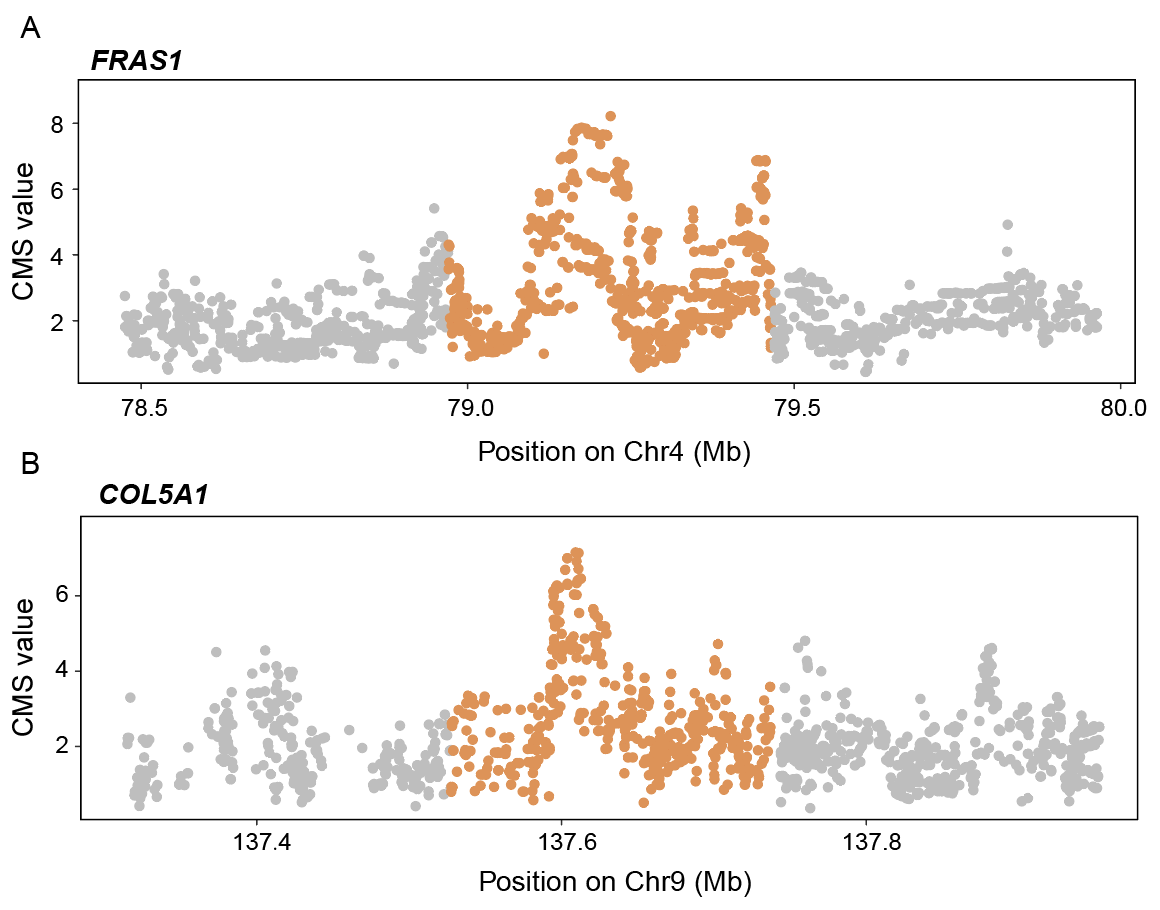


**Fig. S23 | The candidate region (*FRAS1* and *COL5A1*) associated with skin protection**.


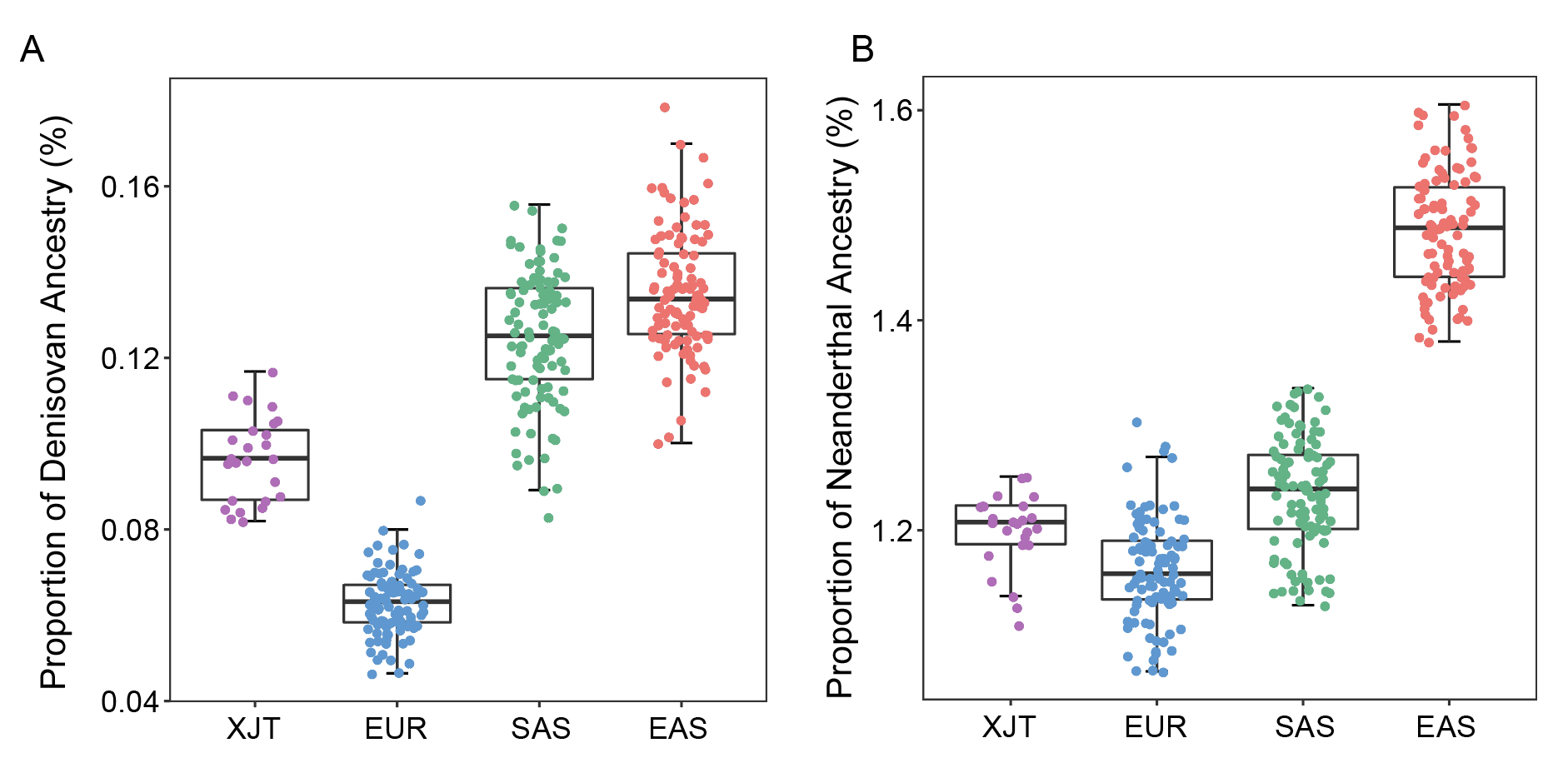


**Fig. S24 | Proportion of archaic introgression**.

**A.** The Denisovan introgression into XJT and the ancestral populations. **B.** The Neanderthal introgression into XJT and the ancestral populations.


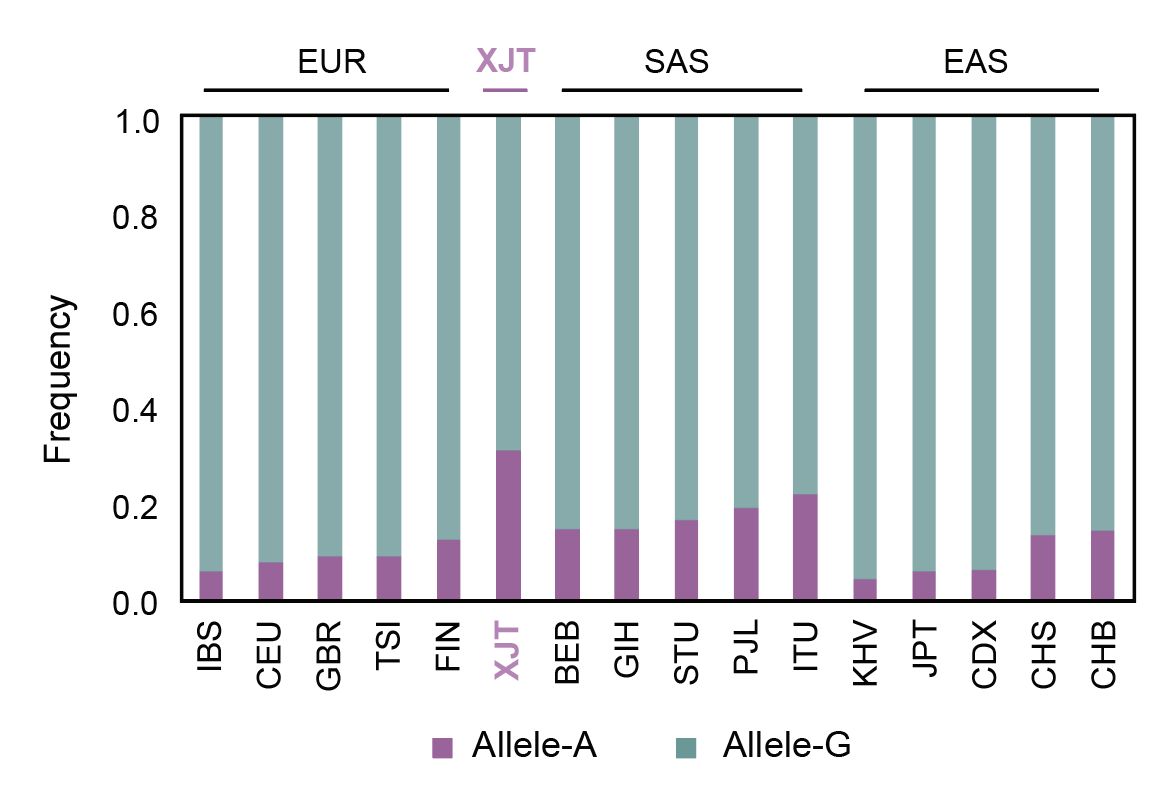


**Fig. S25 | The allele frequency of** **rs79556692 (*LRRC2*) in XJT and KGP dataset**.

**Fig. S26 | Annotation of genes previously reported in high-altitude adaptation studies and also identified in XJT**.


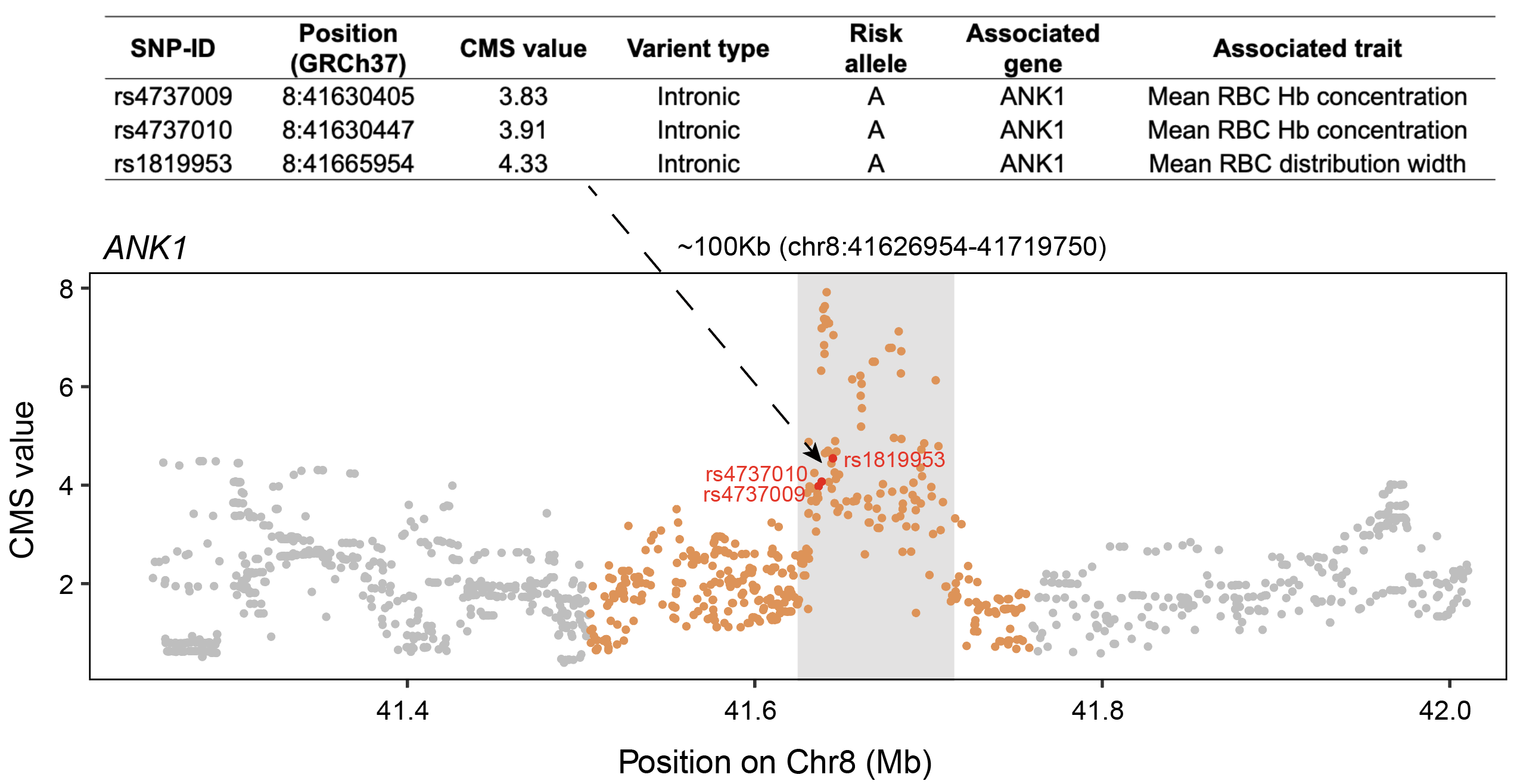


**Fig. S27 | A candidate region associated with mean corpuscular-hemoglobin concentration (MCHC) or mean red cell distribution width (MRDW) in *ANK1***.


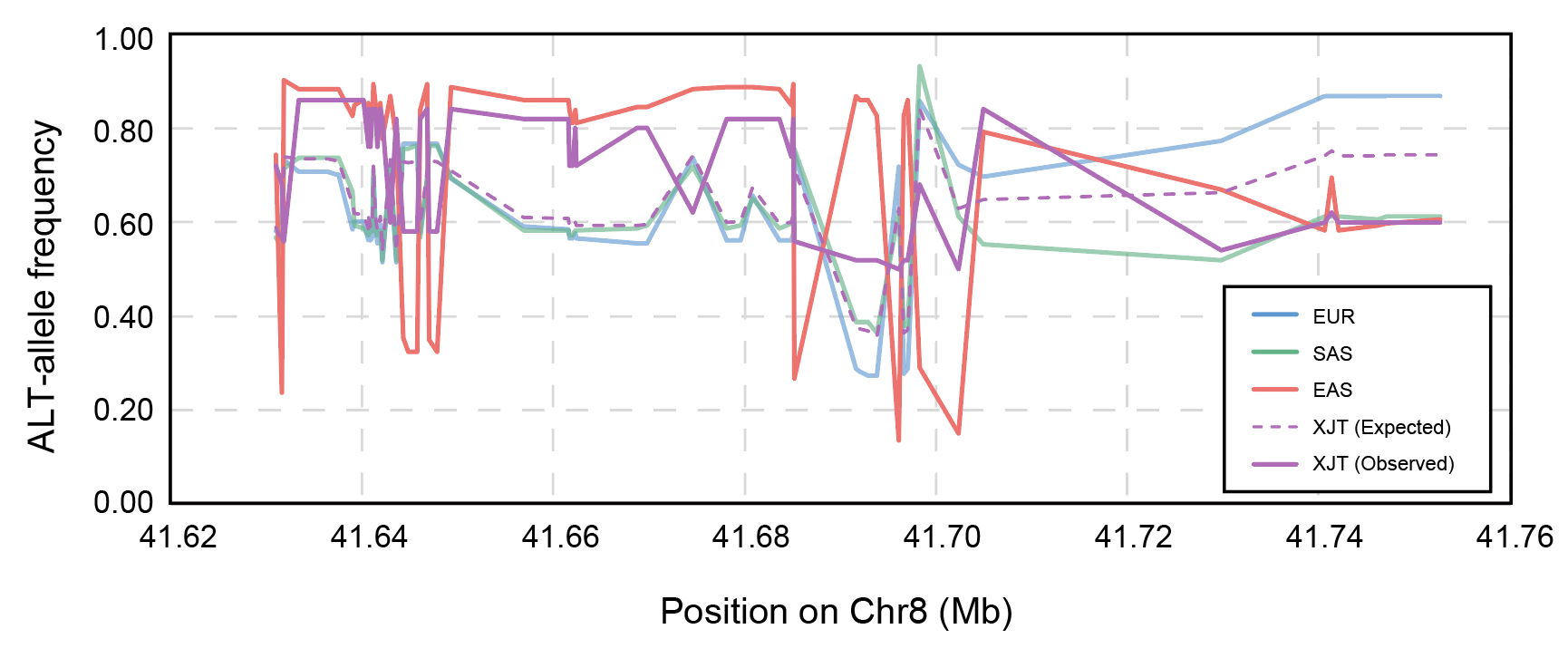


**Fig. S28 | The** **frequency of ALT alleles in SNPs located within the candidate region of the *ANK1****.*


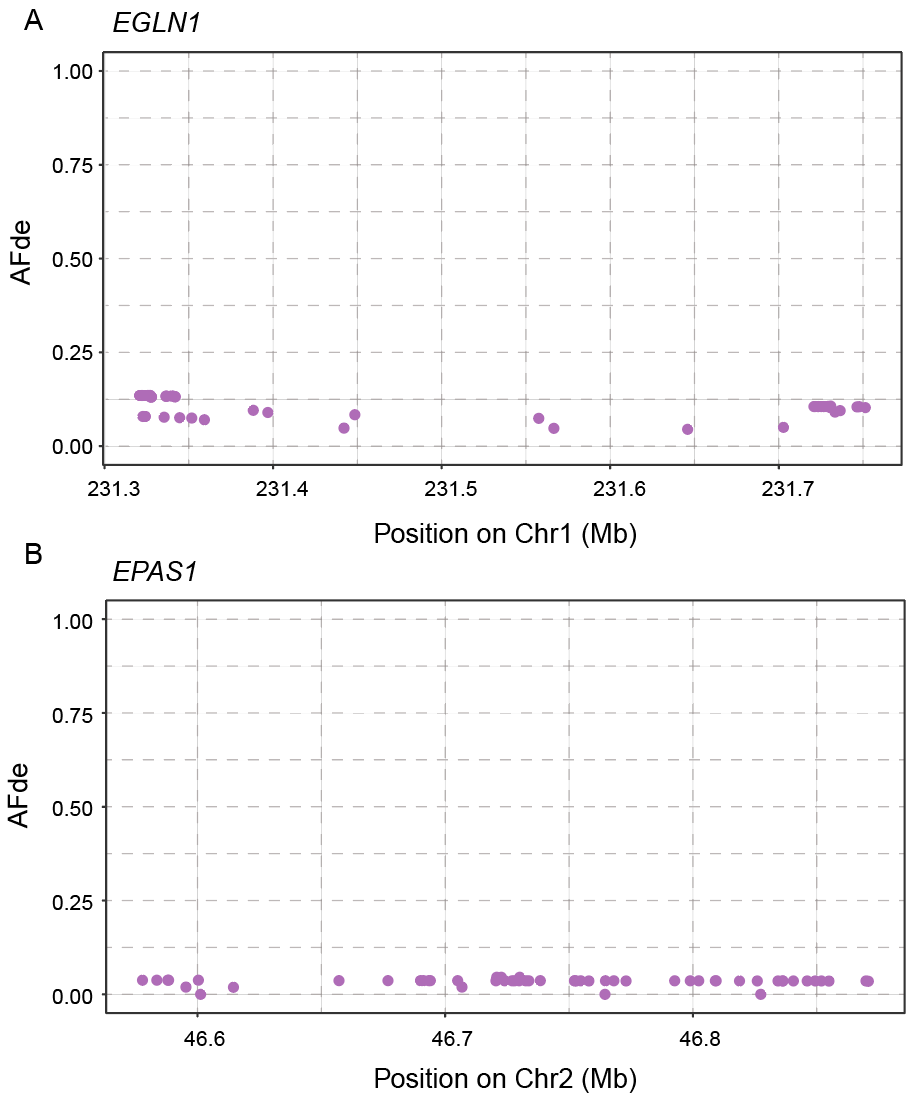


**Fig. S29 | Allele frequency deviation of tag SNPs in *EPAS1*, *EGLN1* for XJT.**


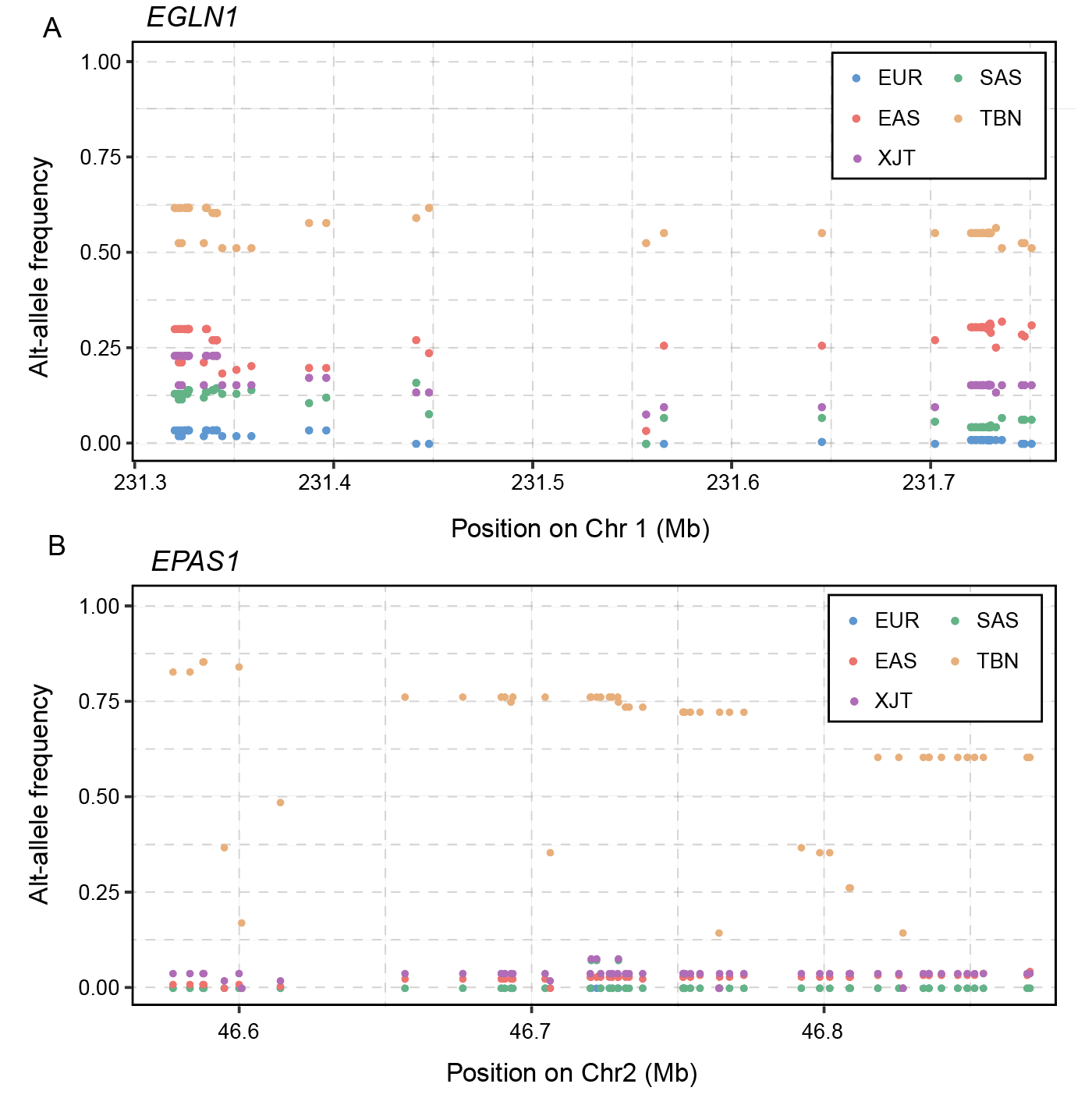


## Fig. S30 | Allele frequency of tag SNPs in *EPAS1*, *EGLN1* for XJT, Tibetans (TBN), and other reference populations.


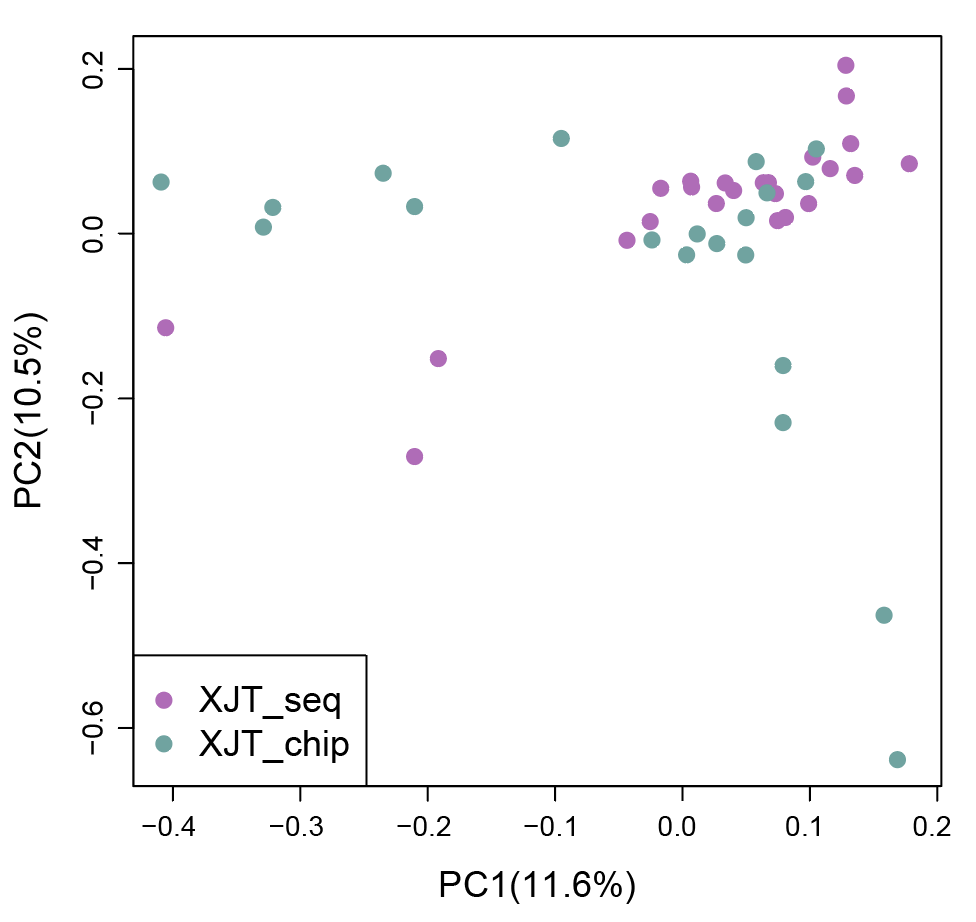


**Fig. S31 | PCA of sequencing and microarray data of XJT**.

# Supplementary Tables

## Table. S**1** | Clinic-protective variants enriched in XJT.

| **Chr** | | **Position (GRCh37)** | | **Ref** | **Alt** | **Protective allele** | **rs_ID** | **CADD_PHRED score** | **GERP ++ score** | **dbSNP155** | **Protective AF** | **Clinic significance (ClinVar)** | **Triat/disease** | **Study** |
| --- | --- | --- | --- | --- | --- | --- | --- | --- | --- | --- | --- | --- | --- | --- |
| 5 | 33951693 | | C | | G | C | rs16891982 | 25.1 | 4.32 | known | 0.375 | benign, association, protective | Pigmentation, melanoma | [29-34] |
| 4 | 100260789 | | T | | C | C | rs698 | 14.23 | 3.94 | known | 0.333 | protective | alcohol metabolism, alcoholism | [35-39] |
| 19 | 49206674 | | G | | A | G | rs601338 | 52 | 4.81 | known | 0.292 | benign, association, protective | plasma vitamin B12, otitis media | [40, 41] |
| 4 | 187004074 | | C | | T | T | rs3775291 | 24 | 4.95 | known | 0.271 | protective | HIV infection | [42] |
| 5 | 169810796 | | C | | T | T | rs11739136 | 20.8 | 3.59 | known | 0.229 | protective | diastolic hypertension | [43-45] |
| 3 | 39307162 | | G | | A | A | rs3732378 | 17.41 | 3.98 | known | 0.229 | pathogenic, risk factor, protective | coronary artery disease | [46] |
| 13 | 108863591 | | G | | A | A | rs1805388 | 18.73 | 4.09 | known | 0.229 | benign, protective | multiple myeloma | [47] |
| 4 | 100504664 | | T | | C | C | rs3816873 | 15.77 | 4.1 | known | 0.146 | protective | metabolic syndrome | [48] |

## Table. S2 | The admixture time of Western ancestries of XJT.

| EUR | English | | | French | | | Estonian | | |
| --- | --- | --- | --- | --- | --- | --- | --- | --- | --- |
| SAS | Vishwabrahmin | Mala | Kharia | Vishwabrahmin | Mala | Kharia | Vishwabrahmin | Mala | Kharia |
| Time | 45.78  +/- 10.19 | 57.62  +/-12.18 | 34.78 +/- 5.26 | 30.47  +/- 7.72 | 37.82  +/- 14.24 | 28.01  +/- 4.11 | 49.04  +/- 22.82 | 64.91  +/- 17.20 | 38.89  +/- 6.19 |
| Z score | 3.70 | 3.70 | 5.74 | 3.95 | 2.54 | 6.13 | 2.15 | 3.37 | 5.29 |
| *P* value | 0.00022 | 0.00021 | 9.7e-09 | 7.9e-05 | 0.011 | 8.6e-10 | 0.032 | 0.00076 | 1.2e-07 |

**Note:** English, French, and Estonian represent ancestries related to European. Vishwabrahmin, Mala, and Kharia represent ancestries related to Southasian. Data of these populations is from HumanOrigins data set with #SNP of 145,569 after combination with micro array SNP data of XJT. 25 years/generation.

## Table. S3 | The admixture time of Eastern and Western ancestries of XJT.

| EUR | English | | | French | | | Estonian | | |
| --- | --- | --- | --- | --- | --- | --- | --- | --- | --- |
| EAS | Han_North | Han_South | Japanese | Han_North | Han_South | Japanese | Han_North | Han_South | Japanese |
| Time | 23.83  +/- 3.88 | 24.01  +/- 3.74 | 23.14 +/- 3.06 | 20.91  +/- 3.04 | 21.48  +/- 3.20 | 20.94 +/- 2.67 | 26.15  +/- 4.35 | 26.23  +/- 4.37 | 25.47  +/- 3.70 |
| Z score | 6.14 | 6.43 | 7.56 | 6.89 | 6.71 | 7.83 | 6.01 | 6.00 | 6.89 |
| *P* value | 8.3e-10 | 1.3e-10 | 3.9e-14 | 5.7e-12 | 2e-11 | 4.9e-15 | 1.9e-09 | 2e-09 | 5.5e-12 |

**Note:** English, French, and Estonian represent ancestries related to European. Han_North, Han_South, and Japanese represent ancestries related to Eastasian. Data of these populations is from HumanOrigins dataset with #SNP of 145,569 after combination with micro array SNP data of XJT. 25 years/generation.

##

## Table. S4 | Pathways enriched for genes of high AFd_e_ in analysis of mGSEA.

| **Pathway** | **# Genes** | **# leading-edge genes** | ***P*-value** |
| --- | --- | --- | --- |
| Cell motility | 199 | 55 | 9.75E-05 |
| Olfactory transduction | 339 | 323 | 9.87E-05 |
| Transport and catabolism | 671 | 159 | 9.91E-05 |
| Rap1 signaling pathway | 199 | 50 | 9.91E-05 |
| Regulation of actin cytoskeleton | 199 | 55 | 9.95E-05 |
| Signal transduction | 1662 | 446 | 9.95E-05 |
| Organismal Systems | 2803 | 629 | 9.98E-05 |
| Human Diseases | 2638 | 605 | 1.00E-04 |
| cAMP signaling pathway | 205 | 60 | 1.00E-04 |
| AMPK signaling pathway | 117 | 40 | 1.00E-04 |
| Signaling molecules and interaction | 770 | 181 | 1.00E-04 |
| Metabolism | 1480 | 334 | 1.00E-04 |
| Nervous system | 502 | 151 | 1.00E-04 |
| Retrograde endocannabinoid signaling | 133 | 41 | 1.01E-04 |
| Oxytocin signaling pathway | 149 | 54 | 1.01E-04 |
| Endocrine system | 876 | 227 | 1.01E-04 |
| Pathways in cancer | 505 | 109 | 1.01E-04 |
| Cell growth and death | 538 | 109 | 1.01E-04 |
| Metabolic pathways | 1291 | 281 | 1.01E-04 |
| Global and overview maps | 1296 | 282 | 1.01E-04 |
| Environmental Information Processing | 2111 | 558 | 1.01E-04 |
| PI3K-Akt signaling pathway | 337 | 83 | 1.01E-04 |
| Endocrine and metabolic disease | 468 | 104 | 1.01E-04 |
| Cellular Processes | 1557 | 363 | 1.01E-04 |
| Digestive system | 390 | 103 | 1.01E-04 |
| Carbohydrate metabolism | 326 | 84 | 1.01E-04 |
| Lipid metabolism | 359 | 77 | 1.01E-04 |
| Cholinergic synapse | 111 | 44 | 1.01E-04 |
| Parathyroid hormone synthesis, secretion and action | 104 | 31 | 1.01E-04 |
| Development and regeneration | 273 | 85 | 1.01E-04 |
| Wnt signaling pathway | 152 | 40 | 1.01E-04 |
| Insulin secretion | 83 | 33 | 1.01E-04 |
| Cellular community - eukaryotes | 526 | 132 | 1.01E-04 |
| Circulatory system | 260 | 76 | 1.01E-04 |
| Cell adhesion molecules (CAMs) | 124 | 38 | 1.01E-04 |
| Vascular smooth muscle contraction | 128 | 36 | 1.01E-04 |
| Adrenergic signaling in cardiomyocytes | 142 | 48 | 1.01E-04 |
| Inflammatory mediator regulation of TRP channels | 99 | 32 | 1.01E-04 |
| Cardiovascular disease | 273 | 68 | 1.01E-04 |
| MAPK signaling pathway | 279 | 72 | 1.01E-04 |
| GABAergic synapse | 84 | 33 | 1.01E-04 |
| Phagosome | 135 | 34 | 1.01E-04 |
| Ras signaling pathway | 220 | 59 | 1.02E-04 |
| Longevity regulating pathway | 87 | 26 | 1.02E-04 |
| Human papillomavirus infection | 316 | 70 | 1.02E-04 |
| Axon guidance | 173 | 61 | 1.02E-04 |
| Immune system | 939 | 211 | 1.02E-04 |
| mTOR signaling pathway | 146 | 39 | 1.02E-04 |
| Glycan biosynthesis and metabolism | 233 | 67 | 1.02E-04 |
| Aldosterone synthesis and secretion | 94 | 30 | 1.02E-04 |
| Long-term potentiation | 64 | 23 | 1.02E-04 |
| Environmental adaptation | 290 | 75 | 1.02E-04 |
| Glutamatergic synapse | 111 | 45 | 1.02E-04 |
| Calcium signaling pathway | 182 | 59 | 1.02E-04 |
| ECM-receptor interaction | 83 | 32 | 1.02E-04 |
| Proteoglycans in cancer | 193 | 49 | 1.02E-04 |
| ABC transporters | 43 | 16 | 1.02E-04 |
| Phospholipase D signaling pathway | 140 | 45 | 1.03E-04 |
| Platelet activation | 121 | 33 | 1.03E-04 |
| Transcriptional misregulation in cancer | 167 | 43 | 1.03E-04 |
| Dopaminergic synapse | 126 | 39 | 1.03E-04 |
| Purine metabolism | 120 | 33 | 1.03E-04 |
| Long-term depression | 56 | 22 | 1.03E-04 |
| Focal adhesion | 189 | 54 | 1.03E-04 |
| Serotonergic synapse | 107 | 38 | 1.03E-04 |
| Synaptic vesicle cycle | 78 | 26 | 1.03E-04 |
| Circadian entrainment | 94 | 34 | 1.03E-04 |
| Insulin resistance | 104 | 32 | 1.03E-04 |
| Choline metabolism in cancer | 93 | 30 | 1.03E-04 |
| ErbB signaling pathway | 79 | 27 | 1.03E-04 |
| Arrhythmogenic right ventricular cardiomyopathy (ARVC) | 71 | 28 | 1.03E-04 |
| Thyroid hormone synthesis | 72 | 25 | 1.03E-04 |
| Apelin signaling pathway | 134 | 44 | 1.04E-04 |
| Morphine addiction | 86 | 35 | 1.05E-04 |
| Nicotine addiction | 36 | 17 | 1.05E-04 |
| MicroRNAs in cancer | 162 | 39 | 2.01E-04 |
| Aging | 99 | 27 | 2.02E-04 |
| Hypertrophic cardiomyopathy (HCM) | 84 | 26 | 2.03E-04 |
| Relaxin signaling pathway | 127 | 33 | 2.04E-04 |
| Gastric acid secretion | 74 | 24 | 2.04E-04 |
| Fc gamma R-mediated phagocytosis | 84 | 25 | 2.05E-04 |
| Salivary secretion | 81 | 25 | 2.05E-04 |
| Gap junction | 83 | 25 | 2.06E-04 |
| Cushing syndrome | 153 | 40 | 2.07E-04 |
| Glycosaminoglycan biosynthesis - heparan sulfate / heparin | 22 | 11 | 2.12E-04 |
| Endocytosis | 225 | 53 | 3.05E-04 |
| Protein digestion and absorption | 80 | 25 | 3.08E-04 |
| Neuroactive ligand-receptor interaction | 310 | 67 | 3.08E-04 |
| Membrane transport | 43 | 16 | 3.09E-04 |
| Dilated cardiomyopathy (DCM) | 89 | 27 | 3.10E-04 |
| Phosphatidylinositol signaling system | 92 | 27 | 4.13E-04 |
| Bile secretion | 71 | 22 | 4.13E-04 |
| Mucin type O-glycan biosynthesis | 29 | 12 | 4.19E-04 |
| Nucleotide metabolism | 143 | 36 | 5.09E-04 |
| PPAR signaling pathway | 74 | 22 | 5.16E-04 |
| Kaposi sarcoma-associated herpesvirus infection | 180 | 42 | 6.12E-04 |
| Inositol phosphate metabolism | 70 | 21 | 9.20E-04 |
| Tight junction | 157 | 37 | 1.10E-03 |
| Pancreatic secretion | 91 | 25 | 1.12E-03 |
| Human cytomegalovirus infection | 213 | 47 | 1.41E-03 |
| Thermogenesis | 194 | 43 | 1.42E-03 |
| Peroxisome | 80 | 22 | 2.15E-03 |
| Glycosphingolipid biosynthesis - ganglio series | 15 | 7 | 2.25E-03 |
| Thyroid hormone signaling pathway | 115 | 28 | 2.75E-03 |
| Human T-cell leukemia virus 1 infection | 207 | 45 | 2.97E-03 |
| Fc epsilon RI signaling pathway | 66 | 19 | 3.04E-03 |
| Glycerolipid metabolism | 49 | 15 | 3.31E-03 |
| Glycerophospholipid metabolism | 85 | 22 | 4.31E-03 |
| Sphingolipid signaling pathway | 118 | 29 | 4.36E-03 |
| Valine, leucine and isoleucine degradation | 46 | 13 | 4.56E-03 |
| Excretory system | 146 | 33 | 4.59E-03 |
| Lysine degradation | 52 | 15 | 5.04E-03 |
| Amino acid metabolism | 263 | 46 | 5.09E-03 |
| Natural killer cell mediated cytotoxicity | 112 | 27 | 5.13E-03 |
| Renin secretion | 66 | 18 | 5.29E-03 |
| Butanoate metabolism | 26 | 9 | 5.35E-03 |
| Taste transduction | 75 | 20 | 5.35E-03 |
| Neurodegenerative disease | 315 | 62 | 5.35E-03 |
| Collecting duct acid secretion | 27 | 9 | 5.86E-03 |
| Ribosome biogenesis in eukaryotes | 64 | 16 | 5.86E-03 |
| Progesterone-mediated oocyte maturation | 85 | 22 | 6.00E-03 |
| Chronic myeloid leukemia | 74 | 19 | 6.09E-03 |
| Chemokine signaling pathway | 178 | 38 | 6.29E-03 |
| Adherens junction | 66 | 18 | 6.59E-03 |
| RNA transport | 135 | 25 | 6.79E-03 |
| Hematopoietic cell lineage | 87 | 22 | 7.03E-03 |
| Inflammatory bowel disease (IBD) | 59 | 15 | 7.06E-03 |
| Lysosome | 117 | 24 | 7.47E-03 |
| Cortisol synthesis and secretion | 63 | 17 | 7.67E-03 |
| Insulin signaling pathway | 131 | 30 | 8.28E-03 |
| Melanogenesis | 100 | 24 | 8.61E-03 |
| Amoebiasis | 90 | 22 | 8.92E-03 |
| Translation | 407 | 71 | 1.01E-02 |
| Human immunodeficiency virus 1 infection | 196 | 24 | 1.07E-02 |
| Th17 cell differentiation | 100 | 41 | 1.09E-02 |
| Cellular senescence | 148 | 32 | 1.10E-02 |
| Rheumatoid arthritis | 82 | 18 | 1.13E-02 |
| Osteoclast differentiation | 117 | 27 | 1.18E-02 |
| Glycosphingolipid biosynthesis - lacto and neolacto series | 27 | 9 | 1.24E-02 |
| Metabolism of other amino acids | 104 | 22 | 1.30E-02 |
| Cardiac muscle contraction | 75 | 19 | 1.36E-02 |
| Linoleic acid metabolism | 29 | 6 | 1.43E-02 |
| Glycosphingolipid biosynthesis - globo and isoglobo series | 14 | 9 | 1.45E-02 |
| GnRH signaling pathway | 91 | 22 | 1.47E-02 |
| Genetic Information Processing | 1129 | 155 | 1.48E-02 |
| alpha-Linolenic acid metabolism | 25 | 8 | 1.66E-02 |
| Sphingolipid metabolism | 42 | 12 | 1.71E-02 |
| HIF-1 signaling pathway | 94 | 22 | 1.73E-02 |
| Salmonella infection | 74 | 22 | 1.77E-02 |
| Glucagon signaling pathway | 95 | 19 | 1.80E-02 |
| EGFR tyrosine kinase inhibitor resistance | 78 | 13 | 1.82E-02 |
| Adipocytokine signaling pathway | 66 | 73 | 1.87E-02 |
| Viral myocarditis | 50 | 17 | 1.88E-02 |
| Endocrine resistance | 94 | 22 | 2.01E-02 |
| Notch signaling pathway | 47 | 13 | 2.30E-02 |
| B cell receptor signaling pathway | 74 | 11 | 2.31E-02 |
| Drug metabolism - other enzymes | 71 | 18 | 2.33E-02 |
| Fatty acid degradation | 42 | 16 | 2.34E-02 |
| Galactose metabolism | 29 | 9 | 2.46E-02 |
| Epstein-Barr virus infection | 189 | 3 | 2.48E-02 |
| Longevity regulating pathway - multiple species | 60 | 15 | 2.52E-02 |
| Metabolism of cofactors and vitamins | 204 | 32 | 2.63E-02 |
| Hippo signaling pathway | 148 | 37 | 2.69E-02 |
| Vitamin B6 metabolism | 6 | 30 | 2.71E-02 |
| VEGF signaling pathway | 59 | 15 | 2.74E-02 |
| Biosynthesis of other secondary metabolites | 9 | 4 | 2.89E-02 |
| Renal cell carcinoma | 62 | 8 | 3.01E-02 |
| Immune disease | 266 | 15 | 3.04E-02 |
| Fructose and mannose metabolism | 30 | 9 | 3.09E-02 |
| Circadian rhythm | 30 | 43 | 3.14E-02 |
| Neurotrophin signaling pathway | 114 | 25 | 3.23E-02 |
| Pentose phosphate pathway | 25 | 8 | 3.44E-02 |
| Viral carcinogenesis | 182 | 35 | 3.61E-02 |
| Toxoplasmosis | 104 | 23 | 3.77E-02 |
| Fatty acid metabolism | 51 | 12 | 3.83E-02 |
| Amino sugar and nucleotide sugar metabolism | 47 | 10 | 4.40E-02 |
| mRNA surveillance pathway | 78 | 18 | 4.50E-02 |
| Autophagy - other | 28 | 16 | 4.66E-02 |
| Epithelial cell signaling in Helicobacter pylori infection | 67 | 8 | 4.72E-02 |
| Endocrine and other factor-regulated calcium reabsorption | 47 | 12 | 4.76E-02 |
| Leukocyte transendothelial migration | 103 | 7 | 4.89E-02 |
| Biosynthesis of unsaturated fatty acids | 22 | 20 | 4.91E-02 |

## Table. S5 | Assignment of genes to bins.

Genes were divided into 17 bins according to SNP numbers (#SNPs_per_gene)

| **bin** | **#SNPs_per_gene** | **#genes_in_bin** | **mean (CMS)** | **sd (CMS)** | **median (CMS)** |
| --- | --- | --- | --- | --- | --- |
| 1 | 1-8 | 1122 | 2.93 | 1.28 | 2.69 |
| 2 | 9-13 | 991 | 3.15 | 1.12 | 2.99 |
| 3 | 14-18 | 1194 | 3.37 | 1.21 | 3.19 |
| 4 | 19-23 | 1179 | 3.39 | 1.05 | 3.24 |
| 5 | 24-28 | 1128 | 3.53 | 1.16 | 3.35 |
| 6 | 29-34 | 1163 | 3.55 | 1.17 | 3.36 |
| 7 | 35-41 | 1089 | 3.62 | 1.07 | 3.43 |
| 8 | 42-49 | 1107 | 3.69 | 1.15 | 3.54 |
| 9 | 50-59 | 1114 | 3.68 | 1.06 | 3.56 |
| 10 | 60-71 | 1096 | 3.83 | 1.13 | 3.66 |
| 11 | 72-88 | 1074 | 3.93 | 1.10 | 3.76 |
| 12 | 89-110 | 1085 | 4.01 | 1.10 | 3.84 |
| 13 | 111-140 | 1074 | 4.12 | 1.19 | 3.93 |
| 14 | 141-188 | 1082 | 4.25 | 1.22 | 4.01 |
| 15 | 189-275 | 1076 | 4.52 | 1.31 | 4.31 |
| 16 | 276-538 | 1080 | 4.81 | 1.26 | 4.61 |
| 17 | 539-9836 | 702 | 5.70 | 1.36 | 5.53 |

## Table. S6 | Significant genes involved in epidermis development.

| **#** | **Gene** | **Chr** | **Start_up5k** | **End_down5k** | **Length(kbp)** | **Max_CMS** | **#SNP** | **Bin** |
| --- | --- | --- | --- | --- | --- | --- | --- | --- |
| 1 | *HES5* | 1 | 2455184 | 2466684 | 11.5 | 6.750188245 | 10 | 2 |
| 2 | *SYNC* | 1 | 33140507 | 33174197 | 33.69 | 6.677448544 | 48 | 8 |
| 3 | *NES* | 1 | 156633555 | 156652189 | 18.634 | 5.873456898 | 26 | 5 |
| 4 | *EDAR* | 2 | 109505927 | 109610828 | 104.901 | 7.57421828 | 157 | 14 |
| 5 | *ADCY5* | 3 | 122996143 | 123173605 | 177.462 | 7.424978855 | 303 | 16 |
| 6 | *FRAS1* | 4 | 78973724 | 79470423 | 496.699 | 8.253118401 | 1027 | 17 |
| 7 | *MSX2* | 5 | 174146536 | 174162896 | 16.36 | 5.85894061 | 8 | 1 |
| 8 | *SLC44A4* | 6 | 31825969 | 31851823 | 25.854 | 5.992090922 | 33 | 6 |
| 9 | *DLL1* | 6 | 170586294 | 170604561 | 18.267 | 7.506076973 | 17 | 3 |
| 10 | *SMARCA2* | 9 | 2010342 | 2198624 | 188.282 | 9.125414291 | 423 | 16 |
| 11 | *COL5A1* | 9 | 137528620 | 137741686 | 213.066 | 7.188511573 | 503 | 16 |
| 12 | *NOTCH1* | 9 | 139383896 | 139445314 | 61.418 | 7.625366728 | 101 | 12 |
| 13 | *DKK1* | 10 | 54069056 | 54082802 | 13.746 | 7.01482173 | 20 | 4 |
| 14 | *KRTAP5-1* | 11 | 1600572 | 1611513 | 10.941 | 8.605627382 | 19 | 4 |
| 15 | *KRTAP5-2* | 11 | 1613409 | 1624524 | 11.115 | 10.05559709 | 22 | 4 |
| 16 | *KRTAP5-3* | 11 | 1623795 | 1634693 | 10.898 | 8.917285516 | 29 | 6 |
| 17 | *KRTAP5-4* | 11 | 1637188 | 1648368 | 11.18 | 8.829711141 | 36 | 7 |
| 18 | *KRTAP5-5* | 11 | 1646033 | 1657160 | 11.127 | 6.934010315 | 22 | 4 |
| 19 | *ALX4* | 11 | 44276994 | 44336716 | 59.722 | 10.7451738 | 118 | 13 |
| 20 | *GAL* | 11 | 68446247 | 68463643 | 17.396 | 5.680310593 | 21 | 4 |
| 21 | *KRT75* | 12 | 52812854 | 52833309 | 20.455 | 7.946568586 | 45 | 8 |
| 22 | *KRT6B* | 12 | 52835435 | 52850910 | 15.475 | 6.104449694 | 32 | 6 |
| 23 | *HOXC13* | 12 | 54327535 | 54345328 | 17.793 | 6.764651425 | 34 | 6 |
| 24 | *MMP14* | 14 | 23300766 | 23323236 | 22.47 | 6.042471298 | 33 | 6 |
| 25 | *PRKCH* | 14 | 61649277 | 62022694 | 373.417 | 7.128205567 | 468 | 16 |
| 26 | *FUZ* | 19 | 50305126 | 50325633 | 20.507 | 5.839627448 | 16 | 3 |
| 27 | *KLK12* | 19 | 51527348 | 51543486 | 16.138 | 8.251351255 | 29 | 6 |
| 28 | *KLK14* | 19 | 51575752 | 51592502 | 16.75 | 5.923954672 | 27 | 5 |
| 29 | *CCT8* | 21 | 30423126 | 30451118 | 27.992 | 5.562921725 | 23 | 4 |
| 30 | *KRTAP13-4* | 21 | 31797572 | 31808216 | 10.644 | 5.302480442 | 6 | 1 |
| 31 | *KRTAP15-1* | 21 | 31807597 | 31818070 | 10.473 | 5.640987794 | 18 | 3 |
| 32 | *KRTAP19-1* | 21 | 31847018 | 31857663 | 10.645 | 5.594671014 | 12 | 2 |
| 33 | *KRTAP19-3* | 21 | 31858782 | 31869275 | 10.493 | 6.084984516 | 8 | 1 |
| 34 | *KRTAP19-4* | 21 | 31864142 | 31874451 | 10.309 | 6.463100184 | 14 | 3 |
| 35 | *KRTAP19-5* | 21 | 31868975 | 31879435 | 10.46 | 6.463100184 | 13 | 2 |
| 36 | *KRTAP19-7* | 21 | 31928194 | 31938633 | 10.439 | 5.770771579 | 14 | 3 |
| 37 | *KRTAP22-2* | 21 | 31957424 | 31967716 | 10.292 | 6.12498236 | 8 | 1 |
| 38 | *KRTAP6-3* | 21 | 31959759 | 31970394 | 10.635 | 6.12498236 | 6 | 1 |
| 39 | *KRTAP6-2* | 21 | 31965909 | 31976219 | 10.31 | 6.12498236 | 14 | 3 |
| 40 | *KRTAP6-1* | 21 | 31980750 | 31991249 | 10.499 | 5.286877703 | 12 | 2 |
| 41 | *KRTAP20-1* | 21 | 31983750 | 31994003 | 10.253 | 5.286877703 | 10 | 2 |
| 42 | *KRTAP20-3* | 21 | 32010183 | 32020455 | 10.272 | 5.26917951 | 6 | 1 |
| 43 | *KRTAP19-8* | 21 | 32405478 | 32415795 | 10.317 | 5.69978727 | 3 | 1 |

##

## Table. S7 | Pathways enriched for genes of high CMS value in analysis of mGSEA.

| Pathway | #Genes | # Leading-edge genes | *P*-value |
| --- | --- | --- | --- |
| Olfactory transduction | 345 | 51 | 0.00179982 |
| Porphyrin and chlorophyll metabolism | 37 | 29 | 0.00389961 |
| Herpes simplex virus 1 infection | 430 | 209 | 0.00469953 |
| Non-homologous end-joining | 12 | 9 | 0.01039896 |
| MAPK signaling pathway | 279 | 224 | 0.0159984 |
| MicroRNAs in cancer | 145 | 114 | 0.01669833 |
| Sensory system | 506 | 60 | 0.01749825 |
| Xenobiotics biodegradation and metabolism | 107 | 42 | 0.01829817 |
| Environmental Information Processing | 2094 | 1517 | 0.01979802 |
| African trypanosomiasis | 36 | 23 | 0.02389761 |
| Gastric acid secretion | 71 | 60 | 0.02469753 |
| Acute myeloid leukemia | 62 | 34 | 0.02519748 |
| Protein export | 21 | 10 | 0.02709729 |
| Drug metabolism - other enzymes | 70 | 34 | 0.02929707 |
| Organismal Systems | 2770 | 2046 | 0.03569643 |
| Fatty acid biosynthesis | 16 | 13 | 0.0359964 |
| Systemic lupus erythematosus | 111 | 16 | 0.04529547 |
| Alzheimer disease | 141 | 104 | 0.04859514 |
| Retrograde endocannabinoid signaling | 133 | 71 | 0.04919508 |

## Table. S8 | The Denisovan introgressed haplotypes significantly enriched in XJT.

| **Archaic_Seg** | **Length(bp)** | **Associated_Gene** | **Chr** | **Pos (GRCh37)** | **rs_ID** | **Archaic_Allele** | **EUR_freq** | **SAS_freq** | **EAS_freq** | **Expected_AlleleFreq** | **Observed_AlleleFreq** | **Diff** |
| --- | --- | --- | --- | --- | --- | --- | --- | --- | --- | --- | --- | --- |
| chr3:46560744- 46593320 | *32576* | *LRRC2* | 3 | 46560744 | rs13099992 | G | 0.076 | 0.146 | 0.141 | 0.109 | 0.32 | 0.211 |
|  |  |  | 3 | 46563822 | rs66627456 | T | 0.076 | 0.146 | 0.141 | 0.109 | 0.32 | 0.211 |
|  |  |  | 3 | 46565341 | rs13093282 | T | 0.076 | 0.146 | 0.141 | 0.109 | 0.32 | 0.211 |
|  |  |  | 3 | 46566242 | rs35056755 | C | 0.076 | 0.146 | 0.141 | 0.109 | 0.32 | 0.211 |
|  |  |  | 3 | 46576916 | rs13083022 | C | 0.076 | 0.146 | 0.141 | 0.109 | 0.32 | 0.211 |
|  |  |  | 3 | 46582367 | rs73069952 | C | 0.076 | 0.146 | 0.141 | 0.109 | 0.32 | 0.211 |
|  |  |  | 3 | 46585769 | rs79556692 | A | 0.076 | 0.146 | 0.141 | 0.109 | 0.32 | 0.211 |

**Note：**All values of allele frequency are AF of archaic alleles.

## Table. S9 | The Neanderthal introgressed haplotypes significantly enriched in XJT.

| **Archaic_Seg** | **Length(bp)** | | **Associated_Gene** | **Chr** | **Pos (GRCh37)** | **rs_ID** | **Archaic_Allele** | **EUR_freq** | **SAS_freq** | **EAS_freq** | **Expected_AlleleFreq** | **Observed_AlleleFreq** | **Diff** |
| --- | --- | --- | --- | --- | --- | --- | --- | --- | --- | --- | --- | --- | --- |
| chr3:22068197: 22074727 | | 6530 | *ZNF385D* | 3 | 22070355 | rs13086174 | A | 0.167 | 0.325 | 0.228 | 0.235 | 0.42 | 0.185 |
|  |  |  |  | 3 | 22071688 | rs1824979 | A | 0.167 | 0.325 | 0.228 | 0.235 | 0.42 | 0.185 |
|  |  |  |  | 3 | 22073133 | rs13061262 | C | 0.167 | 0.282 | 0.194 | 0.215 | 0.42 | 0.185 |
|  |  |  |  | 3 | 22074691 | rs13067918 | T | 0.167 | 0.325 | 0.228 | 0.235 | 0.42 | 0.185 |
| chr6:8400802: 8436445 | | 35643 | *SLC35B3* | 6 | 8402265 | rs2876137 | G | 0.253 | 0.209 | 0.150 | 0.227 | 0.44 | 0.214 |
|  |  |  |  | 6 | 8404757 | rs4959486 | A | 0.253 | 0.209 | 0.150 | 0.227 | 0.44 | 0.214 |
|  |  |  |  | 6 | 8405447 | rs1770820 | T | 0.253 | 0.209 | 0.150 | 0.227 | 0.44 | 0.214 |
|  |  |  |  | 6 | 8406713 | rs1615996 | C | 0.253 | 0.209 | 0.150 | 0.227 | 0.44 | 0.214 |
|  |  |  |  | 6 | 8408059 | rs6905245 | A | 0.253 | 0.209 | 0.150 | 0.227 | 0.44 | 0.214 |
|  |  |  |  | 6 | 8408127 | rs10223544 | T | 0.253 | 0.209 | 0.150 | 0.227 | 0.44 | 0.214 |
|  |  |  |  | 6 | 8410536 | rs2327085 | A | 0.253 | 0.209 | 0.150 | 0.227 | 0.44 | 0.214 |
|  |  |  |  | 6 | 8417521 | rs6905235 | G | 0.253 | 0.209 | 0.150 | 0.227 | 0.44 | 0.214 |
|  |  |  |  | 6 | 8419561 | rs6905235 | T | 0.253 | 0.209 | 0.150 | 0.227 | 0.44 | 0.214 |
|  |  |  |  | 6 | 8432871 | rs1335631 | C | 0.253 | 0.209 | 0.150 | 0.227 | 0.44 | 0.214 |
|  |  |  |  | 6 | 8435830 | rs4142207 | T | 0.227 | 0.194 | 0.150 | 0.208 | 0.44 | 0.214 |
|  |  |  |  | 6 | 8435901 | rs4959488 | T | 0.227 | 0.194 | 0.150 | 0.208 | 0.44 | 0.214 |
|  |  |  |  | 6 | 8436194 | rs915355 | G | 0.227 | 0.194 | 0.150 | 0.208 | 0.44 | 0.214 |

**Note:** All values of allele frequency are AF of archaic alleles

## Table. S10 | The candidate genes involved in high-altitude adaptation previously reported in Ethiopians, Andeans, Tibetans, and Tibetan animals.

| AATF | BNIP3 | DUOXA2 | GRIK2 | MAFG | PIK3C2A | SLC8A1 |
| --- | --- | --- | --- | --- | --- | --- |
| ABL1 | BVES | E2F1 | GRIN2B | MAGI2 | PIK3C2G | SLITRK6 |
| ACACB | C12orf54 | ECE1 | GRM4 | MALT1 | PIK3CA | SMARCD3 |
| ACADSB | C18orf55 | EDAR | GRM5 | MAP2K5 | PIK3CB | SNAI3 |
| ACE | C1orf124 | EDN1 | HBB | MAP2K6 | PIK3CG | SNRNP40 |
| ACVR1B | C4orf7 | EDNRA | HBG2 | MAPP | PIK3R1 | SOCS2 |
| ACVR2A | C9orf3 | EDNRB | HCAR2 | MBNL1 | PIK3R3 | SOD1 |
| ACVRL1 | CACNA1G | EDNRB | HDAC4 | MDH1B | PKLR | SOD3 |
| ADAM17 | CACNA2D1 | EDNRB2 | HDGFRP3 | MITF | PLA2G12A | SPINK2 |
| ADCY1 | CAMK2B | EFEMP1 | HFE | MMP2 | PLCB1 | SPP1 |
| ADCY10 | CAMK2D | EGFR | HFM1-ANF644 | MMP3 | PLEK | SPRTN |
| ADH1A | CASR | EGLN1 | HGF | MSRB3 | PLXNA4 | SPRY2 |
| ADH1B | CBARA1 | EGLN1 (PHD2) | HHAT | MTHFR | POLR2A | SPTA1 |
| ADH1C | CBS | EGLN2 | HIF1A | MT-ND1 | PPARA | SPTLC2 |
| ADH4 | CCL2 | EGLN3 | HIF3A | MT-ND2 | PPARG | SREBF2 |
| ADH5 | CCS | EIF4E1B | HIST1H2BE | MTOR | PPARG | STATA5B |
| ADH6 | CDH13 | EIF4E2 | HIST1H3C | MUT | PPP3CB | STC2 |
| ADH7 | CDH17 | ELF2 | HIST1H4B | MYC | PRDM5 | SUCLG2 |
| ADM | CDH19 | ELTD1 | HLA-DQB1 | MYLK2 | PRDX5 | SYNDIG1 |
| ADORA1 | CDH7 | ENDRA | HLA-DR | MYOF | PRIM2 | SYT1 |
| ADORA2A | CDK2 | ENG | HMBS | NADH6 | PRKAA1 | TAOK2 |
| ADRA1B | CELSR1 | EP300 | HMOX2 | NAGLU | PRKAA2 | TCF7L2 |
| ADRA2A | CHD7 | EP300 | HOMER2 | NARFL | PRKG1 | Tensin3 |
| ADRBK1 | CHRNB2 | EPAS1 | HOXB6 | NCOA1 | PSMC3 | TEX14 |
| AEBP2 | CIC | EPAS1 (HIF2A) | HPH | NEK7 | PSME2 | TGFA |
| AGTPBP1 | CITED2 | EPO | HSD17B12 | NF1 | PTEN | TGFB1 |
| AK9 | C-KIT | ERBB4 | Hsp70Aa | NLK | PTGIS | TGFBR3 |
| ALB | CLEC3B | ERCC4 | Hsp70Ab | NOS1 | PTPRB | TH |
| ALDH2 | CLN5 | ERCC6 | HTR2A | NOS2A | PYGM | THRB |
| ANGPT1 | CNFN | FABP3 | HYOU1 | NOS3 | QP7 | TMEM206 |
| ANGPT2 | CNTN1 | FAIM2 | ID2 | NOTCH1 | RBMS3 | TMEM219 |
| ANGPTL4 | CNTNAP2 | FAM213A | IFI27L1 | NOX4 | RBPJ | TMEM247 |
| ANK1 | COL10A1 | FAM9C | IGF1 | NPAS3 | RBX1 | TMSB4X |
| ANKH | COL6A1 | FANCA | IGFBP1 | NR2E1 | REV1 | TNC |
| ANO1 | COMT | FEN1 | IGFBP2 | NRP1 | RFX3 | TNF |
| ANP32D | COPS5 | FGF10 | IKBKG | NRP2 | RGCC | TNNC1 |
| APOB | CORO1B | FH | IL18BP | NRXN1 | RICTOR | TP53 |
| ARG2 | CRYAA | FIGF | IL1A | NRXN3 | RNF216 | TRIM67 |
| ARHGAP15 | CS | FLT1 | IL1B | OR10X1 | RORA | TSNAX |
| ARNT2 | CSF1R | FOLR1 | IL6 | OR6Y1 | RPA1 | TTLL3 |
| ARPL10L | CST3 | FOLR2 | IMPAD1 | OS9 | RPS6KA5 | TWIST1 |
| ARVCF | CUL3 | FOXM1 | ING2 | OTX1 | RPS6KB2 | USF1 |
| ASB8 | CXCL17 | FOXO1 | ITGA2 | PAFAH1B3 | RUNX1 | VAV3 |
| ATF6 | CXCR4 | FOXS1 | ITGA2 | PAPSS2 | RUNX3 | VDAC1 |
| ATP12A | CYBB | FSHR | ITPR1 | PARD3 | RYR1 | VDR |
| ATP12A | CYLC2 | FXYD6 | ITPR2 | PARVA | RYR2 | VEGFA |
| ATP1A1 | CYP17A1 | GAA | KCNMA1 | PCCA | SATB1 | VEGFB |
| ATP1A2 | CYP26A1/C1 | GATA3 | KCNN2 | PCDH15 | SDHA | VEGFC |
| ATP1B1 | CYP2E1 | GATA6 | KCTD12 | PDE1C | SDHD | VHL |
| ATP6 | DAXX | GCH1 | KCTD13 | PDE2A | SENP1 | WARS2 |
| ATP6V1E2 | DCC | Gcnt3 | KDM1A | PDE4D | SEZ6 | WDPCP |
| ATP8 | DCLK1 | GLDC | KLHL1 | PDE5A | SF3B1 | WHSC1 |
| B4GALT1 | DDIT4 | GLI3 | KRTAP21-2 | PDGF2 | SFRP1 | WWOX |
| BAD | DISC1 | GLUL | LAMB3_ | PDGFC | SFTPD | ZCCHC17 |
| BCDO2 | DNAJC8 | GNAT2 | LEPR | PDGFRA | SIFT | ZDHHC3 |
| BCL3 | DNMT3B | GNB1 | LIPE | PDGFRB | SIRT7 | ZDHHC7 |
| BCO2 | DPP4 | GNG2 | LIPE | PDZD2 | SLC16A14 | ZEB2 |
| BHLHE41 | DPYS | GNPAT | LONP1 | PFKM | SLC1A4 | ZNF638 |
| BMP2 | DPYSL3 | GRIA1 | LRRC3B | PGF | SLC30A9 | ZRANB3 |
| BMPR1B | DST | GRIA4 | LRRC4C | PGR | SLC35F1 |  |
| BMPRII | DUOXA1 | GRID1 | LTF | PHD3 | SLC52A3 |  |

**Note:** Genes marked in red indicated that they are also have selection signals in XJT.

##

## Table. S11 | Significant genes in AFd_e_ analysis that are reported previously.

| **#** | **Gene** | **Chr** | **Start_up5k** | **End_down5k** | **Length(kb)** | ***P*-value** |
| --- | --- | --- | --- | --- | --- | --- |
| 1 | *PRKAA2* | 1 | 57105995 | 57186008 | 80.013 | 0.031 |
| 2 | *LEPR* | 1 | 65881248 | 65891248 | 10.000 | 0.004 |
| 3 | *VAV3* | 1 | 108108782 | 108512766 | 403.984 | 0.009 |
| 4 | *HHAT* | 1 | 210496596 | 210854638 | 358.042 | 0.016 |
| 5 | *TRIM67* | 1 | 231292858 | 231362302 | 69.444 | 0.014 |
| 6 | *GNPAT* | 1 | 231371953 | 231418719 | 46.766 | 0.034 |
| 7 | *DISC1* | 1 | 231757561 | 232182018 | 424.457 | 0.002 |
| 8 | *SLC8A1* | 2 | 40319410 | 40843193 | 523.783 | 0.022 |
| 9 | *WDPCP* | 2 | 63343518 | 64059977 | 716.459 | 0.035 |
| 10 | *PLEK* | 2 | 68587305 | 68629585 | 42.280 | 0.014 |
| 11 | *ZNF638* | 2 | 71498691 | 71667199 | 168.508 | 0.047 |
| 12 | *ERBB4* | 2 | 212235446 | 213408565 | 1173.119 | 0.029 |
| 13 | *EIF4E2* | 2 | 233409762 | 233453354 | 43.592 | 0.007 |
| 14 | *HDAC4* | 2 | 239964864 | 240328348 | 363.484 | 0.006 |
| 15 | *ITPR1* | 3 | 4530032 | 4894524 | 364.492 | 0.047 |
| 16 | *VHL* | 3 | 10177692 | 10198904 | 21.212 | 0.023 |
| 17 | *RBMS3* | 3 | 29317473 | 30056886 | 739.413 | 0.033 |
| 18 | *ZDHHC3* | 3 | 44951749 | 45022677 | 70.928 | 0.026 |
| 19 | *LTF* | 3 | 46472136 | 46531724 | 59.588 | 0.018 |
| 20 | *MITF* | 3 | 69783586 | 70022488 | 238.902 | 0.000 |
| 21 | *CASR* | 3 | 121897530 | 122010342 | 112.812 | 0.044 |
| 22 | *PIK3CB* | 3 | 138367860 | 138558780 | 190.920 | 0.024 |
| 23 | *ALB* | 4 | 74257831 | 74292129 | 34.298 | 0.030 |
| 24 | *PRDM5* | 4 | 121601074 | 121849025 | 247.951 | 0.012 |
| 25 | *ING2* | 4 | 184421147 | 184437249 | 16.102 | 0.046 |
| 26 | *SDHA* | 5 | 213356 | 261815 | 48.459 | 0.000 |
| 27 | *PDE4D* | 5 | 58259865 | 59822947 | 1563.082 | 0.013 |
| 28 | *DPYSL3* | 5 | 146765374 | 146894619 | 129.245 | 0.021 |
| 29 | *STC2* | 5 | 172736716 | 172761506 | 24.790 | 0.049 |
| 30 | *PRIM2* | 6 | 57174603 | 57518375 | 343.772 | 0.010 |
| 31 | *BVES* | 6 | 105539697 | 105590049 | 50.352 | 0.027 |
| 32 | *MAGI2* | 7 | 77641393 | 79087890 | 1446.497 | 0.017 |
| 33 | *CACNA2D1* | 7 | 81570760 | 82078114 | 507.354 | 0.002 |
| 34 | *CNTNAP2* | 7 | 145808453 | 148123090 | 2314.637 | 0.047 |
| 35 | *ANGPT1* | 8 | 108256721 | 108515283 | 258.562 | 0.044 |
| 36 | *B4GALT1* | 9 | 33099080 | 33172354 | 73.274 | 0.045 |
| 37 | *ABL1* | 9 | 133584333 | 133768062 | 183.729 | 0.047 |
| 38 | *PRKG1* | 10 | 52745945 | 54063110 | 1317.165 | 0.043 |
| 39 | *PCDH15* | 10 | 55557531 | 57392702 | 1835.171 | 0.002 |
| 40 | *KCNMA1* | 10 | 78624359 | 79403353 | 778.994 | 0.044 |
| 41 | *GRID1* | 10 | 87354312 | 88131250 | 776.938 | 0.048 |
| 42 | *NOX4* | 11 | 89052524 | 89327779 | 275.255 | 0.010 |
| 43 | *HYOU1* | 11 | 118909900 | 118932913 | 23.013 | 0.006 |
| 44 | *HMBS* | 11 | 118950576 | 118969259 | 18.683 | 0.006 |
| 45 | *FOXM1* | 12 | 2961847 | 2991206 | 29.359 | 0.005 |
| 46 | *AEBP2* | 12 | 19551979 | 19878735 | 326.756 | 0.031 |
| 47 | *SENP1* | 12 | 48431681 | 48505091 | 73.410 | 0.045 |
| 48 | *PFKM* | 12 | 48493922 | 48545187 | 51.265 | 0.045 |
| 49 | *SYT1* | 12 | 79252773 | 79850788 | 598.015 | 0.001 |
| 50 | *DCLK1* | 13 | 36340478 | 36710443 | 369.965 | 0.044 |
| 51 | *CLN5* | 13 | 77559795 | 77581652 | 21.857 | 0.009 |
| 52 | *SPRY2* | 13 | 80905111 | 80920086 | 14.975 | 0.012 |
| 53 | *PCCA* | 13 | 100736269 | 101187686 | 451.417 | 0.009 |
| 54 | *EGLN3* | 14 | 34388437 | 34936980 | 548.543 | 0.025 |
| 55 | *GNG2* | 14 | 52287913 | 52451060 | 163.147 | 0.022 |
| 56 | *ARG2* | 14 | 68081515 | 68123437 | 41.922 | 0.006 |
| 57 | *SPTLC2* | 14 | 77967340 | 78088116 | 120.776 | 0.021 |
| 58 | *NRXN3* | 14 | 78703734 | 80335762 | 1632.028 | 0.013 |
| 59 | *GCNT3* | 15 | 59882074 | 59937438 | 55.364 | 0.030 |
| 60 | *WWOX* | 16 | 78128310 | 79251564 | 1123.254 | 0.047 |
| 61 | *DCC* | 18 | 49861542 | 51062784 | 1201.242 | 0.023 |
| 62 | *CDH7* | 18 | 63412488 | 63553638 | 141.150 | 0.009 |
| 63 | *LONP1* | 19 | 5686845 | 5696845 | 10.000 | 0.015 |
| 64 | *PLCB1* | 20 | 8107824 | 8954003 | 846.179 | 0.012 |

## Table. S12 | Significant genes in CMS analysis that are reported previously.

| **#** | **Symbol** | **Chr** | **Start_up5k** | **End_down5k** | **Length(kbp)** | **Max_CMS** | **#SNP** | **Bin** |
| --- | --- | --- | --- | --- | --- | --- | --- | --- |
| 1 | *EDAR* | 2 | 109505927 | 109610828 | 104.901 | 7.574 | 157 | 14 |
| 2 | *ZDHHC3* | 3 | 44951749 | 45022677 | 70.928 | 6.052 | 50 | 9 |
| 3 | *PIK3CA* | 3 | 178860902 | 178962881 | 101.979 | 7.092 | 82 | 11 |
| 4 | *ALB* | 4 | 74257831 | 74292129 | 34.298 | 5.657 | 39 | 7 |
| 5 | *ELF2* | 4 | 139944266 | 140103372 | 159.106 | 6.128 | 93 | 12 |
| 6 | *HLA-DQB1* | 6 | 32622244 | 32641160 | 18.916 | 7.694 | 446 | 16 |
| 7 | *VEGFA* | 6 | 43732921 | 43759224 | 26.303 | 5.675 | 52 | 9 |
| 8 | *ANK1* | 8 | 41505739 | 41759280 | 253.541 | 7.938 | 427 | 16 |
| 9 | *DPYS* | 8 | 105337552 | 105484281 | 146.729 | 9.013 | 254 | 15 |
| 10 | *TNC* | 9 | 117777806 | 117885536 | 107.73 | 8.840 | 193 | 15 |
| 11 | *NOTCH1* | 9 | 139383896 | 139445314 | 61.418 | 7.625 | 101 | 12 |
| 12 | *PARVA* | 11 | 12393732 | 12557348 | 163.616 | 7.401 | 289 | 16 |
| 13 | *HMBS* | 11 | 118950576 | 118969259 | 18.683 | 6.005 | 31 | 6 |
| 14 | *FAIM2* | 12 | 50255679 | 50303000 | 47.321 | 6.515 | 66 | 10 |
| 15 | *SPRY2* | 13 | 80905111 | 80920086 | 14.975 | 7.286 | 11 | 2 |
| 16 | *PCCA* | 13 | 100736269 | 101187686 | 451.417 | 7.562 | 529 | 16 |
| 17 | *NLK* | 17 | 26363763 | 26528407 | 164.644 | 5.574 | 20 | 4 |
| 18 | *SEZ6* | 17 | 27276919 | 27338458 | 61.539 | 6.537 | 40 | 7 |

# Reference

1. Patterson N, Price AL, Reich D. Population structure and eigenanalysis. *PLoS Genet*. 2006; **2**(12): e190. doi: 10.1371/journal.pgen.0020190

2. Price AL, Patterson NJ, Plenge RM *et al.* Principal components analysis corrects for stratification in genome-wide association studies. *Nat Genet*. 2006; **38**(8): 904-909. doi: 10.1038/ng1847

3. Howie BN, Donnelly P, Marchini J. A flexible and accurate genotype imputation method for the next generation of genome-wide association studies. *PLoS Genet*. 2009; **5**(6): e1000529. doi: 10.1371/journal.pgen.1000529

4. Genomes Project C, Auton A, Brooks LD *et al.* A global reference for human genetic variation. *Nature*. 2015; **526**(7571): 68-74. doi: 10.1038/nature15393

5. Delaneau O, Zagury JF, Robinson MR *et al.* Accurate, scalable and integrative haplotype estimation. *Nat Commun*. 2019; **10**(1): 5436. doi: 10.1038/s41467-019-13225-y

6. Lazaridis I, Patterson N, Mittnik A *et al.* Ancient human genomes suggest three ancestral populations for present-day Europeans. *Nature*. 2014; **513**(7518): 409-413. doi: 10.1038/nature13673

7. Mallick S, Li H, Lipson M *et al.* The Simons Genome Diversity Project: 300 genomes from 142 diverse populations. *Nature*. 2016; **538**(7624): 201-206. doi: 10.1038/nature18964

8. Weir BS, Cockerham CC. Estimating F-Statistics for the Analysis of Population Structure. *Evolution*. 1984; **38**(6): 1358-1370. doi: 10.1111/j.1558-5646.1984.tb05657.x

9. Danecek P, Auton A, Abecasis G *et al.* The variant call format and VCFtools. *Bioinformatics*. 2011; **27**(15): 2156-2158. doi: 10.1093/bioinformatics/btr330

10. Purcell S, Neale B, Todd-Brown K *et al.* PLINK: a tool set for whole-genome association and population-based linkage analyses. *Am J Hum Genet*. 2007; **81**(3): 559-575. doi: 10.1086/519795

11. Yu G, Smith DK, Zhu H *et al.* ggtree: an r package for visualization and annotation of phylogenetic trees with their covariates and other associated data. *Methods in Ecology and Evolution*. 2016; **8**(1): 28-36. doi: 10.1111/2041-210x.12628

12. Alexander DH, Novembre J, Lange K. Fast model-based estimation of ancestry in unrelated individuals. *Genome Res*. 2009; **19**(9): 1655-1664. doi: 10.1101/gr.094052.109

13. Jakobsson M, Rosenberg NA. CLUMPP: a cluster matching and permutation program for dealing with label switching and multimodality in analysis of population structure. *Bioinformatics*. 2007; **23**(14): 1801-1806. doi: 10.1093/bioinformatics/btm233

14. Price AL, Tandon A, Patterson N *et al.* Sensitive detection of chromosomal segments of distinct ancestry in admixed populations. *PLoS Genet*. 2009; **5**(6): e1000519. doi: 10.1371/journal.pgen.1000519

15. Ni X, Yuan K, Liu C *et al.* MultiWaver 2.0: modeling discrete and continuous gene flow to reconstruct complex population admixtures. *Eur J Hum Genet*. 2019; **27**(1): 133-139. doi: 10.1038/s41431-018-0259-3

16. Pan Y, Zhang C, Lu Y *et al.* Genomic diversity and post-admixture adaptation in the Uyghurs. *Natl Sci Rev*. 2022; **9**(3): nwab124. doi: 10.1093/nsr/nwab124

17. Durinck S, Spellman PT, Birney E *et al.* Mapping identifiers for the integration of genomic datasets with the R/Bioconductor package biomaRt. *Nat Protoc*. 2009; **4**(8): 1184-1191. doi: 10.1038/nprot.2009.97

18. Subramanian A, Tamayo P, Mootha VK *et al.* Gene set enrichment analysis: a knowledge-based approach for interpreting genome-wide expression profiles. *Proc Natl Acad Sci U S A*. 2005; **102**(43): 15545-15550. doi: 10.1073/pnas.0506580102

19. Szpiech ZA, Hernandez RD. selscan: an efficient multithreaded program to perform EHH-based scans for positive selection. *Mol Biol Evol*. 2014; **31**(10): 2824-2827. doi: 10.1093/molbev/msu211

20. Grossman SR, Shlyakhter I, Karlsson EK *et al.* A composite of multiple signals distinguishes causal variants in regions of positive selection. *Science*. 2010; **327**(5967): 883-886. doi: 10.1126/science.1183863

21. Kircher M, Witten DM, Jain P *et al.* A general framework for estimating the relative pathogenicity of human genetic variants. *Nat Genet*. 2014; **46**(3): 310-315. doi: 10.1038/ng.2892

22. Siepel A, Bejerano G, Pedersen JS *et al.* Evolutionarily conserved elements in vertebrate, insect, worm, and yeast genomes. *Genome Res*. 2005; **15**(8): 1034-1050. doi: 10.1101/gr.3715005

23. Davydov EV, Goode DL, Sirota M *et al.* Identifying a high fraction of the human genome to be under selective constraint using GERP++. *PLoS Comput Biol*. 2010; **6**(12): e1001025. doi: 10.1371/journal.pcbi.1001025

24. Yu G, Wang LG, Han Y *et al.* clusterProfiler: an R package for comparing biological themes among gene clusters. *OMICS*. 2012; **16**(5): 284-287. doi: 10.1089/omi.2011.0118

25. Yu G, Wang LG, Yan GR *et al.* DOSE: an R/Bioconductor package for disease ontology semantic and enrichment analysis. *Bioinformatics*. 2015; **31**(4): 608-609. doi: 10.1093/bioinformatics/btu684

26. Yuan K, Ni X, Liu C *et al.* Refining models of archaic admixture in Eurasia with ArchaicSeeker 2.0. *Nat Commun*. 2021; **12**(1): 6232. doi: 10.1038/s41467-021-26503-5

27. Browning SR, Browning BL, Zhou Y *et al.* Analysis of Human Sequence Data Reveals Two Pulses of Archaic Denisovan Admixture. *Cell*. 2018; **173**(1): 53-61 e59. doi: 10.1016/j.cell.2018.02.031

28. Racimo F, Marnetto D, Huerta-Sanchez E. Signatures of Archaic Adaptive Introgression in Present-Day Human Populations. *Mol Biol Evol*. 2017; **34**(2): 296-317. doi: 10.1093/molbev/msw216

29. Graf J, Hodgson R, van Daal A. Single nucleotide polymorphisms in the MATP gene are associated with normal human pigmentation variation. *Hum Mutat*. 2005; **25**(3): 278-284. doi: 10.1002/humu.20143

30. Yuasa I, Umetsu K, Harihara S *et al.* Distribution of the F374 allele of the SLC45A2 (MATP) gene and founder-haplotype analysis. *Ann Hum Genet*. 2006; **70**(Pt 6): 802-811. doi: 10.1111/j.1469-1809.2006.00261.x

31. Stokowski RP, Pant PV, Dadd T *et al.* A genomewide association study of skin pigmentation in a South Asian population. *Am J Hum Genet*. 2007; **81**(6): 1119-1132. doi: 10.1086/522235

32. Fernandez LP, Milne RL, Pita G *et al.* SLC45A2: a novel malignant melanoma-associated gene. *Hum Mutat*. 2008; **29**(9): 1161-1167. doi: 10.1002/humu.20804

33. Guedj M, Bourillon A, Combadieres C *et al.* Variants of the MATP/SLC45A2 gene are protective for melanoma in the French population. *Hum Mutat*. 2008; **29**(9): 1154-1160. doi: 10.1002/humu.20823

34. Stacey SN, Sulem P, Masson G *et al.* New common variants affecting susceptibility to basal cell carcinoma. *Nat Genet*. 2009; **41**(8): 909-914. doi: 10.1038/ng.412

35. Hoog JO, Heden LO, Larsson K *et al.* The gamma 1 and gamma 2 subunits of human liver alcohol dehydrogenase. cDNA structures, two amino acid replacements, and compatibility with changes in the enzymatic properties. *Eur J Biochem*. 1986; **159**(2): 215-218. doi: 10.1111/j.1432-1033.1986.tb09855.x

36. Xu YL, Carr LG, Bosron WF *et al.* Genotyping of human alcohol dehydrogenases at the ADH2 and ADH3 loci following DNA sequence amplification. *Genomics*. 1988; **2**: 209-214. doi: 10.1016/0888-7543(88)90004-3

37. Osier M, Pakstis AJ, Kidd JR *et al.* Linkage Disequilibrium at the ADH2 and ADH3 Loci and Risk of Alcoholism. *American Journal of Human Genetics*. 1999; **64**(4): 1147-1157. doi: Doi 10.1086/302317

38. Chai YG, Oh DY, Chung EK *et al.* Alcohol and aldehyde dehydrogenase polymorphisms in men with type I and Type II alcoholism. *Am J Psychiatry*. 2005; **162**(5): 1003-1005. doi: 10.1176/appi.ajp.162.5.1003

39. Edenberg HJ. The genetics of alcohol metabolism: role of alcohol dehydrogenase and aldehyde dehydrogenase variants. *Alcohol Res Health*. 2007; **30**(1): 5-13.

40. Hazra A, Kraft P, Selhub J *et al.* Common variants of FUT2 are associated with plasma vitamin B12 levels. *Nat Genet*. 2008; **40**(10): 1160-1162. doi: 10.1038/ng.210

41. Santos-Cortez RLP, Chiong CM, Frank DN *et al.* FUT2 Variants Confer Susceptibility to Familial Otitis Media. *Am J Hum Genet*. 2018; **103**(5): 679-690. doi: 10.1016/j.ajhg.2018.09.010

42. Sironi M, Biasin M, Cagliani R *et al.* A common polymorphism in TLR3 confers natural resistance to HIV-1 infection. *J Immunol*. 2012; **188**(2): 818-823. doi: 10.4049/jimmunol.1102179

43. Fernandez-Fernandez JM, Tomas M, Vazquez E *et al.* Gain-of-function mutation in the KCNMB1 potassium channel subunit is associated with low prevalence of diastolic hypertension. *J Clin Invest*. 2004; **113**(7): 1032-1039. doi: 10.1172/JCI20347

44. Senti M, Fernandez-Fernandez JM, Tomas M *et al.* Protective effect of the KCNMB1 E65K genetic polymorphism against diastolic hypertension in aging women and its relevance to cardiovascular risk. *Circ Res*. 2005; **97**(12): 1360-1365. doi: 10.1161/01.RES.0000196557.93717.95

45. Nielsen T, Burgdorf KS, Grarup N *et al.* The KCNMB1 Glu65Lys polymorphism associates with reduced systolic and diastolic blood pressure in the Inter99 study of 5729 Danes. *J Hypertens*. 2008; **26**(11): 2142-2146. doi: 10.1097/HJH.0b013e32830b894a

46. Moatti D, Faure S, Fumeron F *et al.* Polymorphism in the fractalkine receptor CX3CR1 as a genetic risk factor for coronary artery disease. *Blood*. 2001; **97**(7): 1925-1928. doi: DOI 10.1182/blood.V97.7.1925

47. Roddam PL, Rollinson S, O'Driscoll M *et al.* Genetic variants of NHEJ DNA ligase IV can affect the risk of developing multiple myeloma, a tumour characterised by aberrant class switch recombination. *J Med Genet*. 2002; **39**(12): 900-905. doi: DOI 10.1136/jmg.39.12.900

48. Rubin D, Helwig U, Pfeuffer M *et al.* A common functional exon polymorphism in the microsomal triglyceride transfer protein gene is associated with type 2 diabetes, impaired glucose metabolism and insulin levels. *J Hum Genet*. 2006; **51**(6): 567-574. doi: 10.1007/s10038-006-0400-y
